# Light spectrum modifies the utilization pattern of energy sources in *Pseudomonas* sp. DR 5-09

**DOI:** 10.1371/journal.pone.0189862

**Published:** 2017-12-21

**Authors:** Samareh Gharaie, Lea A. I. Vaas, Anna Karin Rosberg, Sofia T. Windstam, Maria E. Karlsson, Karl-Johan Bergstrand, Sammar Khalil, Walter Wohanka, Beatrix W. Alsanius

**Affiliations:** 1 Swedish University of Agricultural Sciences, Department of Biosystems and Technology, Microbial Horticulture Unit, Alnarp, Sweden; 2 Fraunhofer IME-ScreeningPort, Hamburg, Germany; 3 State University of New York, Department of Biological Sciences, Oswego, New York, United States of America; 4 Geisenheim University, Department of Phytomedicine, Geisenheim, Germany; Universite Paris-Sud, FRANCE

## Abstract

Despite the overruling impact of light in the phyllosphere, little is known regarding the influence of light spectra on non-phototrophic bacteria colonizing the leaf surface. We developed an *in vitro* method to study phenotypic profile responses of bacterial pure cultures to different bands of the visible light spectrum using monochromatic (blue: 460 nm; red: 660 nm) and polychromatic (white: 350–990 nm) LEDs, by modification and optimization of a protocol for the Phenotype MicroArray^™^ technique (Biolog Inc., CA, USA). The new protocol revealed high reproducibility of substrate utilization under all conditions tested. Challenging the non-phototrophic bacterium *Pseudomonas* sp. DR 5–09 with white, blue, and red light demonstrated that all light treatments affected the respiratory profile differently, with blue LED having the most decisive impact on substrate utilization by impairing respiration of 140 substrates. The respiratory activity was decreased on 23 and 42 substrates under red and white LEDs, respectively, while utilization of one, 16, and 20 substrates increased in the presence of red, blue, and white LEDs, respectively. Interestingly, on four substrates contrasting utilization patterns were found when the bacterium was exposed to different light spectra. Although non-phototrophic bacteria do not rely directly on light as an energy source, *Pseudomonas* sp. DR 5–09 changed its respiratory activity on various substrates differently when exposed to different lights. Thus, ability to sense and distinguish between different wavelengths even within the visible light spectrum must exist, and leads to differential regulation of substrate usage. With these results, we hypothesize that different light spectra might be a hitherto neglected key stimulus for changes in microbial lifestyle and habits of substrate usage by non-phototrophic phyllospheric microbiota, and thus might essentially stratify leaf microbiota composition and diversity.

## Introduction

Epiphytic phyllosphere colonization is a function of environmental conditions and their dynamics, such as water and nutrient availability, temperature, irradiation including UV irradiation, and plant properties (plant species, leaf morphology and topography, composition of cuticle waxes, leaf exudate quantity and composition) [1–4]. Within this context, multiple phyllosphere interactions are regulated by light. Alsanius et al. [5] presented a concept for biotic and abiotic interactivities between light, the plant and plant leaf, abiotic factors, and the phyllosphere microbiota. From the perspective of the plant and plant leaf, the function of the light factor must be separated with respect to diurnal dynamics and day length, light intensity, and light spectrum, but also with respect to temperature. For plants, the short-wave blue (425–475 nm) and long-wave red (625–675 nm) parts of the visible light spectrum are important for conversion of light energy into low- and high-molecular organic compounds, i.e. photosynthesis [6]. For epiphytic leaf colonizers, photosynthesis as a process has two decisive functions. First, it is the motor for growth and development of the matrix for epiphytic phyllosphere colonizers, a process which is dependent on plant water and nutrient availability and uptake [6, 7]. It thereby also interacts with other abiotic/microclimatic factors prevailing in the phyllosphere (humidity, temperature, shade). Second, photosynthesis is the essential process for formation of organic nutrient sources, some of which are exuded through the cuticle to the leaf surface becoming readily available to heterotrophic bacteria. Leaf exudation is dependent on plant leaf properties (plant species, plant nutrient and water status, natural and artificial exudation sites, composition and thickness of the waxy layer) [2, 8, 9] and environmental factors, such as humidity and temperature [10, 11]. Readily available nutrients are not evenly distributed on the leaf surface or over time [12], leading to a patchy distribution of bacterial aggregates on the leaf surface.

Apart from indirect effects of light mediated by the plant and plant leaf, light is highly likely to affect the microbial leaf colonizers directly, as many phototrophic and chemotrophic bacteria are able to sense light [13–17]. Photosensory systems include six receptor protein families, namely cryptochrome, blue light-sensing proteins using FAD (BLUF), light oxygen voltage receptor domain (LOV), photoactive yellow protein (PYP), rhodopsin, and phytochromes [18, 19]. Although Propst-Ricutti and Lubin [20] showed that sporulation of *Bacillus subtilis* is inhibited by short light wavelengths and stimulates the formation of fruiting bodies in *Stigmatella aurantiaca* [21], recent studies indicate that the light spectrum influences major lifestyle processes of non-phototrophic bacteria, including motility, surface attachment, formation and inhibition of biofilm, and response to oxidative stress [15, 22–24].

In contrast to the impact of visible light, the deleterious features of ultraviolet light and its impact on microbial cells are well established. The three UV classes, UVA (315–400 nm), UVB (280–315 nm), and UVC (100–280 nm), contribute differently to cell death. For example, the generation of reactive oxygen species kills microbial cells during exposure to UVA, whereas the lethal effect of UVB is caused by direct DNA damage. Photoprotection is exhibited by light-sensitive pigments, e.g., carotenoids, quenching toxic oxygen species. DNA damage can be repaired by different repair systems. One direct repair system is photoreactivation using photolyase and blue light for energy generation [25]. On studying culturable bacteria colonizing peanut leaves, Sundin and Jacobs [26] concluded that UVR tolerance is an important characteristic of phyllosphere bacteria. They also noted seasonal variations, with an increase in UVB-tolerant isolates in late season compared with early season and a change in community structure as a result of leaf exposure to UVB [27]. Similar findings have been reported by Kadivar et al. [28] for corn leaves exposed to UVB using culture-independent analysis. Pigmentation has been identified as an important feature in withstanding UV radiation in the phyllosphere [29]. Thus, for microbial leaf colonizers, light spectrum, intensity, and heat adaptation are important assets, but equally essential is the ability of the microorganism to cope with oscillating levels of water and nutrient availability, temperature, humidity, oxidative stress, and changing light spectra.

This complexity of light-bacteria interactions in the phyllosphere illustrates the challenges when studying the phenotypic response of phyllosphere colonizers in the presence of different wavelengths of light. This might have contributed to the relatively small number of studies examining direct interactions between phyllosphere-colonizing bacteria and light, despite the overruling impact of light on all organisms.

In order to untangle this complexity, we established a simplistic, mid-throughput method facilitating nutrient usage profiling of single strain phyllosphere colonizers when facing light spectra decisive for plant photosynthesis. We mimicked the nutritionally fluctuating leaf environment using the Phenotype MicroArray^™^ (PM) technique (Biolog Inc., Hayward CA, USA). Our proof of principle is based on the phenotypic plasticity of a non-fluorescent *Pseudomonas* sp. DR 5–09 strain isolated from greenhouse-grown *Impatiens walleriana*. Proteobacteria, in particular the class Gammaproteobacteria and the genus *Pseudomonas*, have been found to be ubiquitous in the phyllosphere [5, 30]. So far, no information is available regarding their response to different light spectra.

In this study, we investigated the utilization of different sole energy sources by the phyllosphere-colonizing bacterium *Pseudomonas* sp. DR 5–09 in the presence of different light conditions (darkness and white, red, and blue light emitting diodes (LED)). We modified the procedure of the PM technique to enable assessment of respiratory phenotypic response of bacteria to different light spectra and of the chosen *Pseudomonas* sp. DR 5–09 strain. We also examined how substrate utilization patterns depend on light spectrum exposure. Utilization of carbon, nitrogen, and phosphorus substrates by *Pseudomonas* sp. DR 5–09 was determined in darkness and in the presence of different colors of light, by determining maximum curve height.

## Material and methods

Due to their importance for photosynthesis in plants and greenhouse crop production, monochromatic red and blue wavelengths and also polychromatic white light spectra were chosen for this study. Utilization of sole nutrient sources was monitored as color changes in a tetrazolium blue-based redox dye, which is colorless in reduced and purple in oxidized state, reflecting respiratory activity [31, 32]. This technique, which was employed for acquisition of respiratory phenotypes, involved the Phenotype MicroArray^™^ (PM) technique (Biolog Inc., Hayward CA, USA), which offers up to 949 different nutritional and environmental conditions under dark incubation. The technique was customized for application in light conditions.

### Transmittance of light at different wavelengths through selected cover materials

To assure optimal transmission of light through the cover material, light transmittance of the lids provided with the PM plates was compared with that of seven other covering materials, as listed in S1 Table.

**Fig 1:**
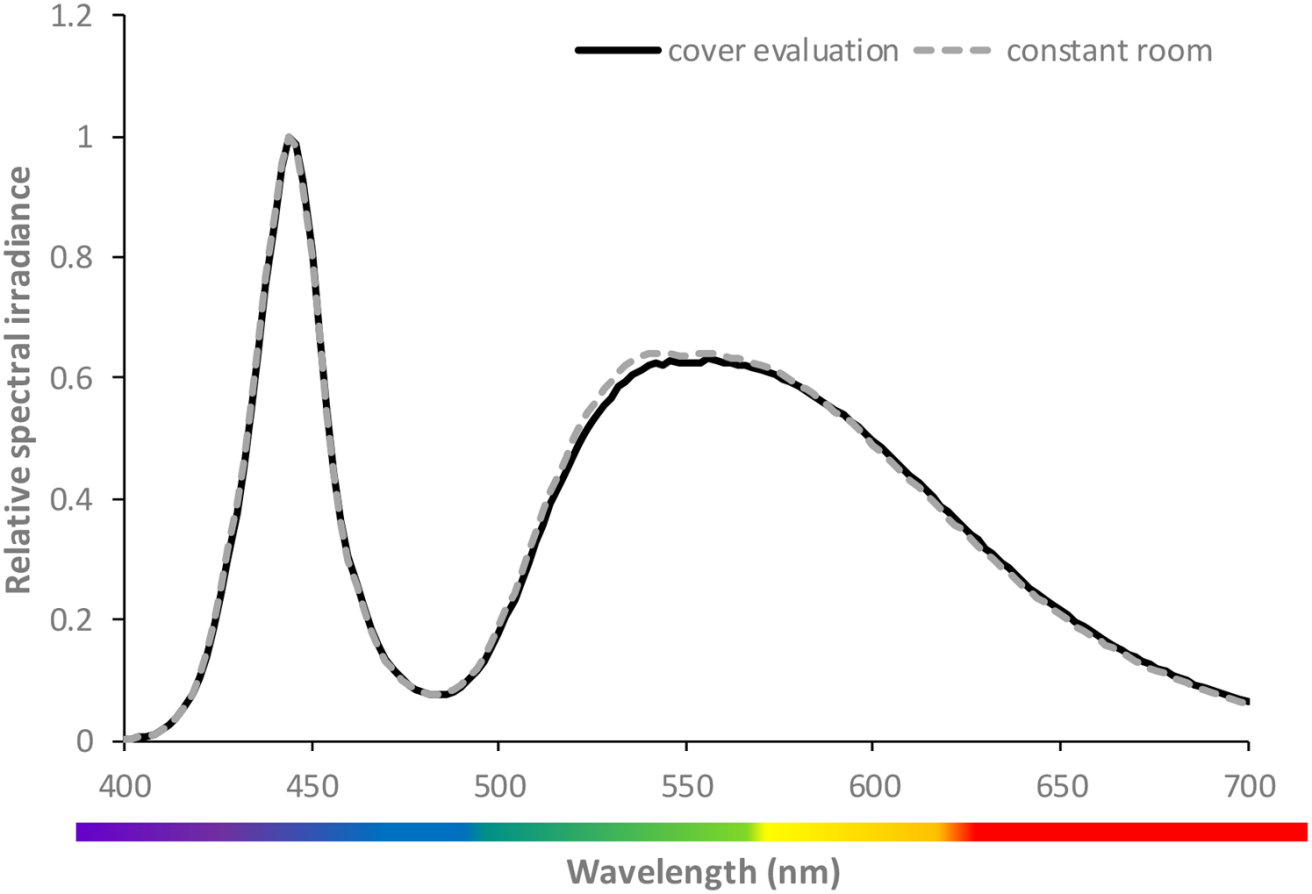
Relative spectral irradiance emitted by white LEDs for transmittance assessment (black line) and for irradiation during incubation in the climate chamber (grey broken line). The curves represent the mean of three individual replicates.

Lid material was exposed to three LED light sources (white; Fig 1), red (660 nm), and a combination of red (660 nm) and blue (460 nm) (80/20). Transmittance of each material was measured on three independent replicates using a spectroradiometer (Li-Cor Li-1800, Li-Cor, Lincoln, NE USA). Measurements without any material between the light source and the spectroradiometer served as a positive control.

### Microbial strain and its propagation

The non-fluorescent *Pseudomonas* sp. strain IO5, isolated from greenhouse-grown *Impatiens walleriana*, was used as a model strain, representative of phyllosphere-inhabiting bacteria. This strain is a producer of protease, chitinase, and biosurfactants. IO5 was *de novo* sequenced and a BLASTn analysis using the NCBI gene bank demonstrated that the strain shares 93% similarity with *Pseudomonas* sp. DR 5–09 (Query cover: 63%; E-value: 0: Identification: 93%) and the strain will from here-on be referred to as strain D 5–09. *Pseudomonas* sp. DR 5–09 was propagated on Tryptic Soy Agar (TSA, DF 0369-17-6; Difco Laboratories Inc., Detroit, MI, USA) and incubated overnight at 30°C, before being prepared for phenotypic microarray measurements. DR 5–09 was grown in Tryptic Soy Broth (TSB, Difco Laboratories Inc, Detroit, MI, USA) for 18 h at 25°C to generate cells for growth curve analysis.

### Phenotypic profiling assay of *P*. sp. DR 5–09 under different light conditions

The PM assays applied here involved panel PM01 through PM04 (Biolog Inc., USA, Catalog number 12111, 12112, 12121, and 12131, respectively). These four panels (S2 Table) consist of 190 sole carbon (C), 95 nitrogen (N), 59 phosphorus (P), and 35 sulfur (S) sources. Substrate concentrations differ between the four panels: C (2 to 20 mM), N (1 to 5 mM), P (0.1 to 1 mM), and S (0.1 to 1 mM) (B. Bochner, pers. comm. 2016). Carbon sources (PM01 and PM02) were incubated as sole substrate, whereas N, P, and S sources (PM03 and PM04) were supplemented with 2 mM sodium succinate and 2 μM ferric citrate as additional carbon sources (enrichment). In addition to C utilization, 37 compounds served as model substrates to study their impact as N (L-alanine, L-arginine, L-asparagine, L-aspartic acid, L-cysteine, L-glutamic acid, L-glutamine, glycine, L-histidine, L-isoleucine, L-leucine, L-lysine, L-methionine, L-phenylalanine, L-proline, L-serine, L-threonine, L-valine, D-alanine, D-aspartic acid, D-serine, L-homoserine, L-ornithine, N-acetyl-L-glutamic acid, L-pyroglutamic acid, putrescine, tyramine, acetamide, glucuronamide, D-glucosamine, N-acetyl-D-glucosamine, N-acetyl-D-galactosamine, adenosine, thymidine, uridine, inosine; P (D-glucose-1-phosphate, D-glucose-6-phosphate); and S (L-cysteine, L-methionine) sources. PM assays were performed according to the standard protocols recommended by the manufacturer for gram-negative bacteria. In brief, colony swabs were used to harvest *Pseudomonas* sp. DR 5–09 cells from overnight cultures, which were then suspended in IF-0a GN medium (Biolog Inc., Hayward, CA, USA). The turbidity of the bacterial suspension was adjusted turbidimetrically (Biolog Inc., USA, Catalog number 3587) to 85% transmittance, before the redox dye (Dye mix A; catalog no. 74221; Biolog Inc., Haywood, USA) was added. A volume of 100 μL of the suspension was pipetted into each plate well. Thereafter, plates were sealed with Greiner ViewSeal (Greiner Bio-one, 676070; Sigma Aldrich, Z617571-100EA, St Louis, MO, USA), selected on the basis of the transmittance test for 96-well plates, and subjected either to darkness or to white (350–990 nm), red (660 nm), or blue (460 nm) LEDs. Six independent replicates were collected under each light regime. Panels exposed to darkness were kept in the OmniLog reader (OmniLog, catalog number 93182, Biolog Inc., USA) during the entire incubation period at 20°C with 15 min measurement intervals over a 96-h period. Light exposure took place in lined cabinets (500 mm x 500 mm x 1000 mm), which were arranged in a climate room (constant temperature 20°C) that allowed eight plates to be run simultaneously. Each cabinet was equipped with a LED lamp (90 W, Trädgårdsteknik AB, Ängelholm, Sweden) with peak wavelengths at 460 nm (blue), 660 nm (red), or a continuous spectrum between350 to 990 nm (white). The spectral output of the lamps was measured using a spectroradiometer (Li-Cor Li-1800, Li-Cor, Lincoln, NE USA) and light intensity was adjusted to 100 μmol m^-2^ s^-1^ by arrangement of suitable distances between the light source and the PM plates. Color change in LED-exposed panels was measured by repeated short readings using the Omnilog reader at distinct time points over one hour (0 h, 7 h, 14 h, 21 h, 24 h, 28 h, 36 h, 42 h, 48 h, 54 h, 60 h, 66 h, 72 h, 78 h, 84 h, 90 h, and 96 h).

In order the verify that selected light spectra and intensities did not cause a significant growth impairment of *Pseudomonas* sp DR 5–09, growth curves under the same LED lights and light intensities were collected. Ten μl of bacterial overnight culture in TSB was used to inoculate 150 μl TSB in each well of 96-well microtiter plates which were then covered with Greiner ViewSeal film (Greiner Bio-one, Sigma Aldrich, St Louis, MO, USA) and incubated in darkness, or under white, red, or blue LED lights. Four replicate wells were harvested at 30–60 min intervals and the absorbance at 620 nm was measured using a spectrophotometer (ASYS Hitech Expert 96, Biochrom, Cambourne, UK). A subset of samples were serially diluted in 0.085% NaCl and spot plated in triplicate on TSA to collect viable population sizes after recording absorbance. Four replicates per light treatment were plated and viable counts were assessed during lag, log, and beginning of stationary phase. To examine if *Pseudomonas* sp DR 5–09 cells were negatively impacted by light spectra and intensities when confronted with only one nutrient source, as opposed to a complex medium like TSB, the bacterial viability was established over a 96 h time period. Based on nutrient utilization data, D-mannose was selected as the sole carbon source, as Biolog data indicated a similar utilization level of this carbon source by *Pseudomonas* kept in darkness compared to when cells were exposed to blue light (S2 File). Cultures of *Pseudomonas* sp. DR 5–09 were generated in TSB as described above and cells were harvested by centrifugation at 3,000 × *g* at 4°C for 15 min and then washed twice using M9 minimal medium. Cells were then resuspended in M9 medium amended with 10 mM D-mannose to generate a viable starting inoculum of 6.7 log 10 cfu/ml, which was verified by serial dilution and spot plating of dilutions in triplicate onto TSA. Cell suspension aliquots of 160 μl per well were placed into 96-well plates which were sealed with Greiner ViewSeal film and then incubated at 20°C under continuous light treatments (dark, white, red, and blue LED) at the same intensities as Biolog assays. At 24, 30, 48, 54, 72, 78, and 96 hpi 150 μl from four replicate treatment wells were transferred to a microtiter plate and the optical density at 620 nm was measured. At 24, 48, 72, and 96 hpi, 50 μl aliquots from four replicate wells were serially diluted and dilutions were spot plated in triplicate to assess viability. This experiment was carried out in two independent trials.

### Calculation and bioinformatics

Analysis of light transmission through plate lid and covering materials was performed on the basis of spectral output data. Percentage transmission through the covering material at decisive wavelengths (444 nm, 454 nm, 556 nm, and 664 nm) was analyzed using Anova followed by Tukey test (p<0.05) using Minitab vers 16.2.4 (Minitab Inc., State College Pennsylvania). Viable population densities of bacteria exposed to darkness, white, red, and blue LED lights were compared using one-way Anova with light source as the predictor and viable counts as the response factor. Within individual time points, Tukey’s comparison of means (p<0.05) was conducted.

After data recording and export to.csv-files using OmniLog^®^ PM kinetic analysis software (Product Number UA24331—PMM, version 1.6), all further data management steps, graphical representations, and statistical analyses of PM data were performed using R [33] and functionality from the dedicated R package opm [34]. Raw data were arranged and the parameters length of lag phase, maximum curve height, area under the curve, and slope were calculated as previously described by Vaas et al. [35], see S1 Fig for more details. Raw kinetic read data, including calculated curve parameters, are available in S1 File. Impaired substrate utilization patterns were compared with biochemical pathways using the Kyoto Encyclopedia of Genes and Genomes (KEGG; http://www.genome.jp/kegg/) to identify the probable metabolic impact of light spectra.

## Results

### Film transmittance

**Fig 2:**
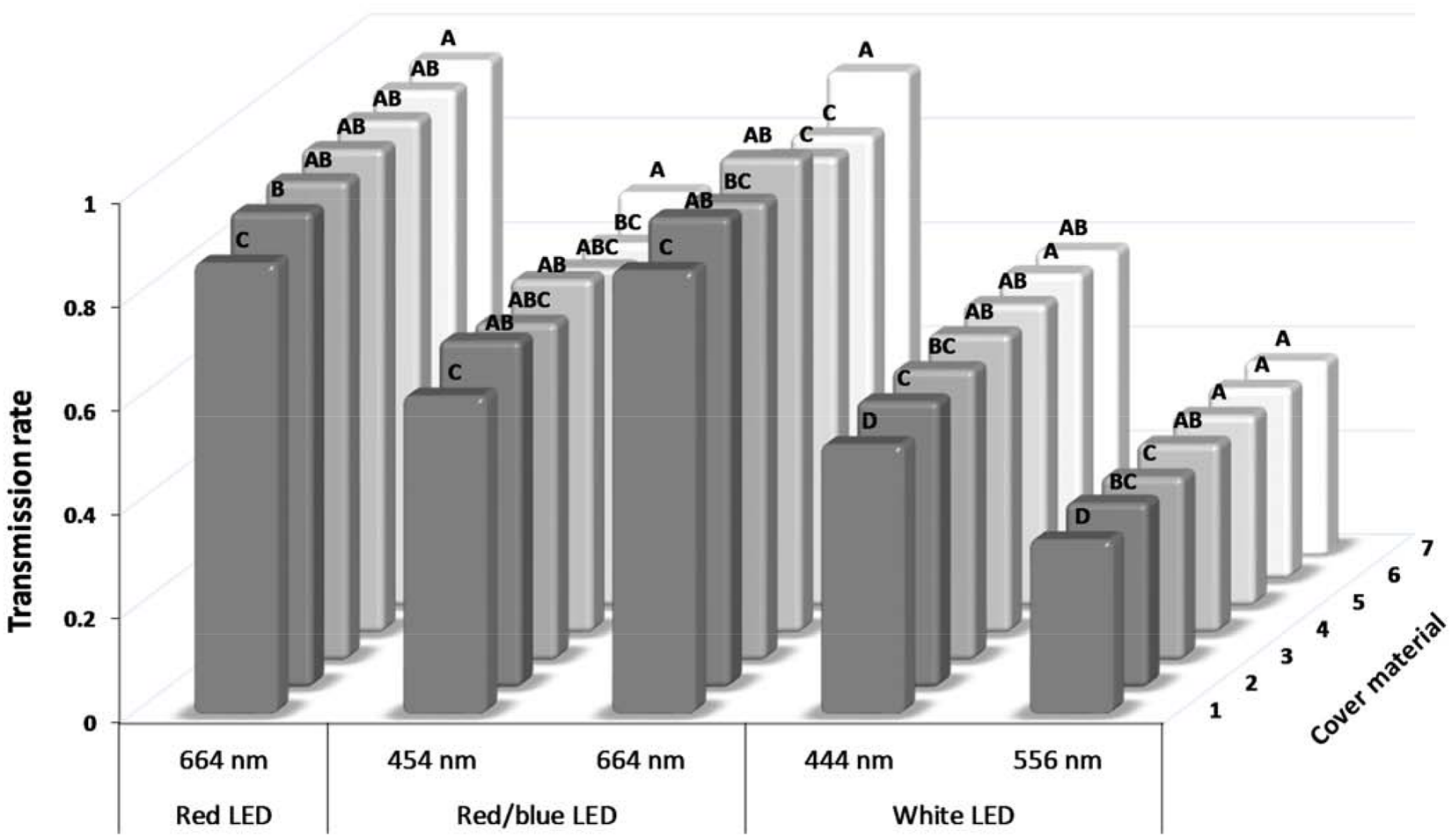
Transmittance [% of control] of LED light through different cover materials for microtiter plates. The cover materials are presented in S1 Table. Bars within wavelengths labeled with different letters are significantly different (Tukey-test; p<0.05 with n = 3).

Unfiltered white LED displayed two peaks, at wavelengths 444 and 556 nm (Fig 1), whereas the red LED showed a peak at 664 nm. The main wavelengths emitted from the combined red and blue LED source occurred at 454 nm and 664 nm. These wavelength peaks corresponded well with the narrow wavelength spectra of the blue and red LEDs utilized in this study. These LEDs exhibited peak wavelength emissions as 460 and 660 nm, respectively. In general, the covering lids of the microtiter plates transmitted the test wavelengths at a lower level than the other cover materials screened (Fig 2). Best transmittance was found for the transparent cover film 7, i.e. the self-adhering Greiner ViewSeal for 96-well plates (Fig 2), and it was therefore chosen as the most appropriate film for all subsequent assays.

### Substrate utilization patterns of *Pseudomonas* sp. DR 5–09 exposed to different light spectra

In total, 379 substrates and conditions on four pre-fabricated panels were included in assays and each panel was repeated independently six times under all four light regimes. The xy-plots depicting the raw kinetics of all replicates comparing each light condition to dark treatment displayed high reproducibility (S2 File).

**Fig 3:**
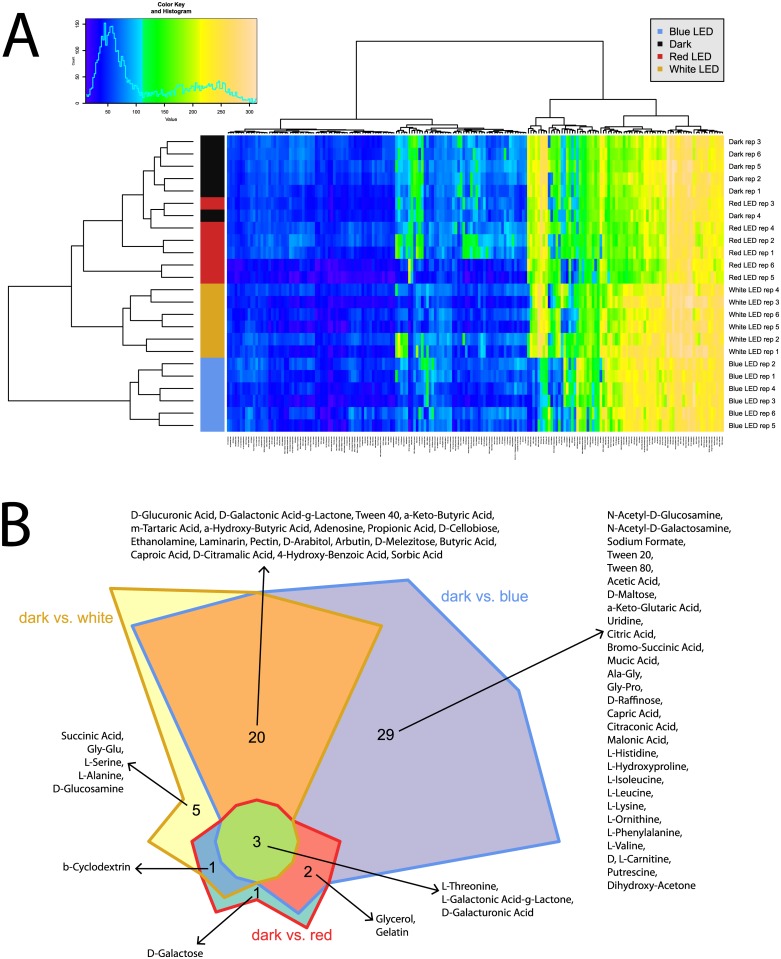
Overview of respiration behavior of *Pseudomonas* sp. DR 5–09 on 190 carbon (C) sources tested. (A) Heatmap of maximum height values of 190 C sources when exposed to blue, red, and white LEDs or darkness, expressed as maximum curve height monitored during 96 h of incubation. The legend (upper corner to the left) explains the color code from blue to green, while yellow shades indicate low, moderate, and high utilization of C sources, assessed as arbitrary Omnilog values. The histogram describes the frequency of maximum height reached for C sources. (B) Chow Rusky diagram of sole C utilization patterns in which respiration of *Pseudomonas* sp. DR 5–09 was affected (significantly different to dark incubation) by exposure to blue, red, and white LEDs.

**Fig 4:**
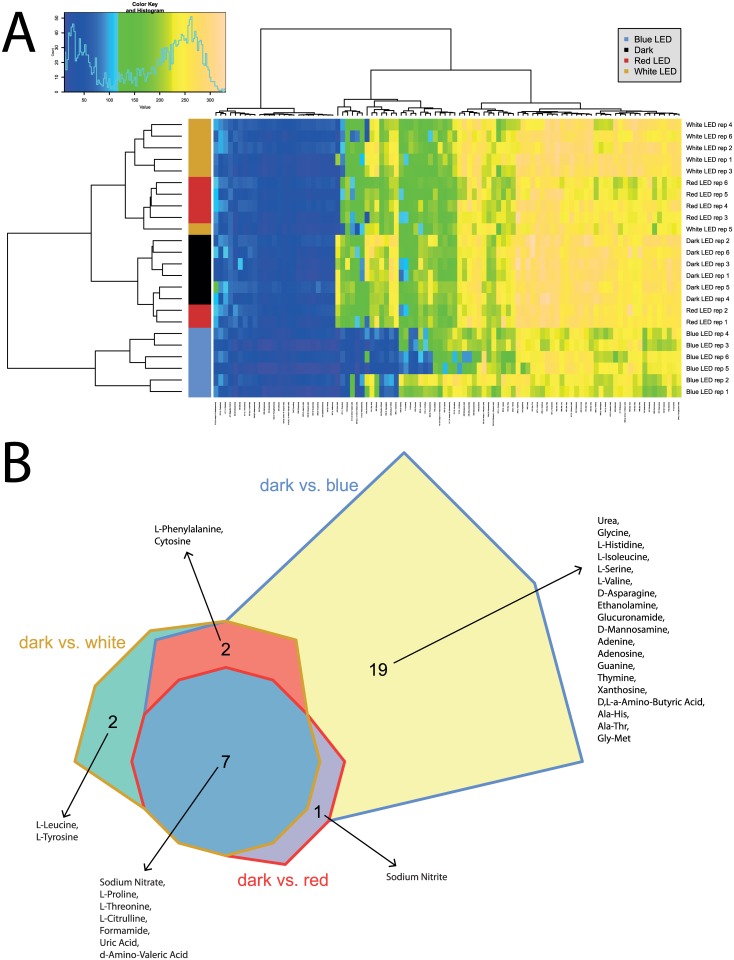
Overview of respiration behavior of *Pseudomonas* sp. DR 5–09 on 95 nitrogen (N) sources tested. (A) Heatmap of utilization of 95 nitrogen (N) sources by *Pseudomonas* sp. DR5-09 when exposed to blue, red, and white LEDs or darkness, expressed as maximum curve height monitored during 96 h of incubation. The legend (upper corner to the left) explains the color code from blue to green, while yellow shades indicate low, moderate, and high utilization of N sources, assessed as extinction (Omnilog values). The histogram describes the frequency of utilization of different N sources. (B) Chow Rusky diagram of N substrate utilization patterns by *Pseudomonas* sp. DR 5–09 incubated in darkness compared with incubation under blue, red, or white LEDs (significantly different to dark incubation).

**Fig 5:**
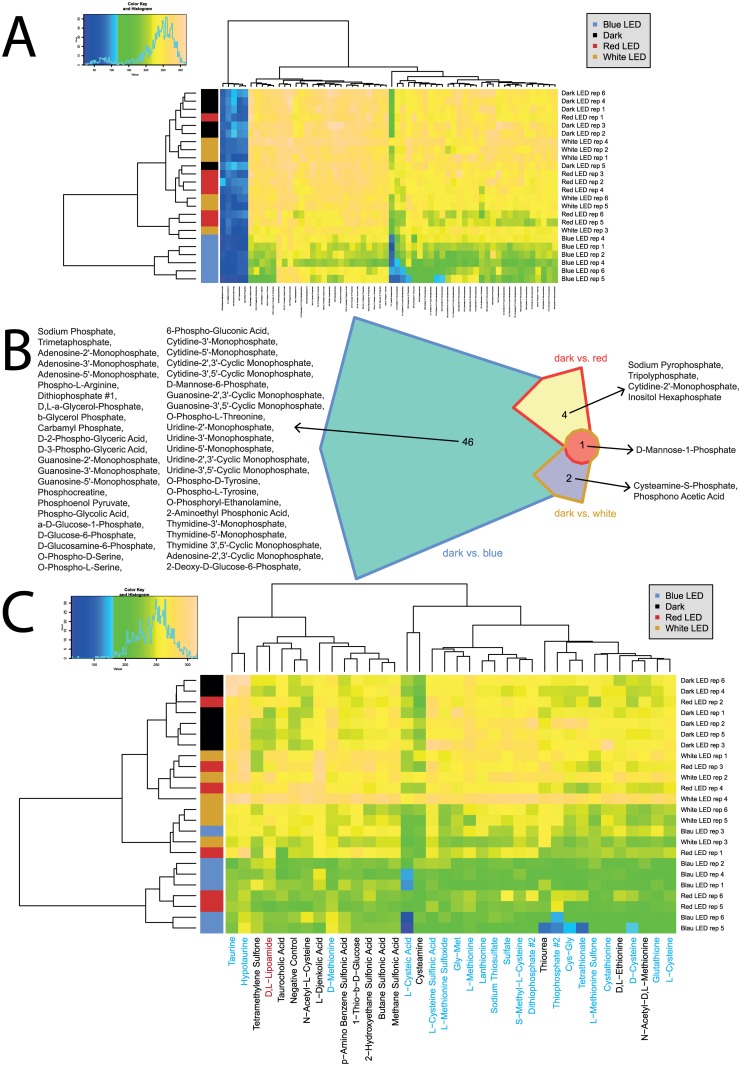
Overview of respiration behavior of Pseudomonas sp. DR 5–09 on 59 phosphorous (P) sources and 35 sulfur (S) sources tested. (A) Heatmap of utilization of 59 phosphorus (P) sources by *Pseudomonas* sp. DR 5–09 when exposed to blue, red, and white LEDs or darkness, expressed as maximum curve height monitored during 96 h of incubation. The legend (upper corner to the left) explains the color code from blue to green, while yellow shades indicate low, moderate, and high utilization of P sources, assessed as extinction (Omnilog values). The histogram describes the frequency of utilization of different P sources. (B) Chow Rusky diagram of P substrate utilization patterns by *Pseudomonas* sp. DR 5–09 incubated in darkness compared with incubation under blue, red, or white LEDs (significantly different from dark incubation). (C) Heatmap of utilization of 35 sulfur (S) sources by *Pseudomonas* sp. DR 5–09 when exposed to blue, red, and white LEDs or darkness, expressed as maximum curve height monitored during 96 h of incubation. Substrate names in blue and red denote substrates significantly affected by blue and red light treatment, respectively.

The parameter maximum curve height (A) was chosen for detailed analysis of respiratory profiles, but all parameters were investigated using heatmaps (S2 Fig). To gain a general overview of the utilization patterns exhibited, heatmaps were compiled (Figs 3–5). Interestingly, use of C, N, and P sources discriminated well between the light treatments when considering the maximum curve height (A), while grouping of C and P sources occurred when considering the area under the kinetic curve (AUC), see S2 Fig. The utilization of S-sources by *Pseudomonas*. sp. DR 5–09 was not affected by any of the light regimes applied (Fig 5C).

As an overview of general utilization, this section will only discuss broad changes in utilization of substrates under different light regimes. In the next subsection, we will provide detailed information about specific changes. In general, for a substantial number of sole carbon sources offered in PM01 and PM02, no or only minor respiration could be observed. These reactions were not affected by any of the light treatments or the dark treatment tested. However, on the basis of responses to the 190 C sources tested (panels PM01 and PM02), a distinct response pattern was detected for each light regimen and thus allowed separation of all four light regimes (Fig 3A). Utilization of C sources under dark conditions and under red LED during incubation clustered together, indicating only a minor influence of red LED on the maximum curve height reached. The cluster of these two treatments could be distinguished from the treatments using white and blue LED, respectively. A similar, but less distinct, trend was found for AUC, where blue light incubation of sole C sources deviated from the other three treatments (see S2 Fig). Compared with utilization in darkness, the blue, red, and white LED treatments exclusively affected 29, five, and one substrates, respectively (Fig 3B). The utilization of 20 specific C sources was affected by both blue and white LEDs compared with the control. Likewise, blue and red LEDs and white and red LEDs affected the respiration of two and one specific C-based substrates. Only three distinct substrates were utilized under all three light regimes, namely L-threonine, L-galactonic acid-g-lactone, and D-galacturonic acid.

Utilization of the 95 N sources included in the panel (PM03) was in general considerably higher than for C sources, both with respect to frequencies and maximum color change. Analysis of N utilization confirmed the deviating pattern by *P*. sp. DR 5–09 in the presence of blue light regarding the maximum curve height (A) (Fig 4A). The strong impact of blue light on respiration was reflected by the number of substrates affected by blue LED only (19 substrates), whereas no (red) or few (white: 2; L-leucine, L-tyrosine) substrates were affected by red and white LEDs only (Fig 4B). The bacterium utilized the amino acids L-threonine, D-asparagine, and L-isoleucine, as well as cytosine, D,L-α-amino-N-butyric acid, D-mannosamine, nitrate, and nitrite, when exposed to red and white LEDs and to darkness, but no respiratory activity was detected when incubated under blue LED. Utilization of uric acid was supported during dark incubation, but counteracted by all three light regimes. In the presence of blue light, N-source utilization was always below the maximum height level of dark conditions.

Similar analysis of utilization patterns for 59 P sources included in the PM panel (subset of plate PM04) showed that substrate utilization patterns of *P*. *sp*. DR 5–09 in the presence of red and white LEDs and in darkness clustered well together, while the utilization pattern on exposure to blue LED deviated (Fig 5A). Only six of 59 P sources did not differ significantly when comparing the blue LED treatment with dark treatment. These were triethyl phosphate, hypophosphite, adenosine 3’,5’-cyclic monophosphate, thiophosphate, phosphorylcholine, and methylene diphosphonic acid (Fig 5B). In contrast, most P-based substrates were metabolized to the same extent under red and white light as during dark incubation. Significantly lower respiration was found for sodium pyrophosphate (p = 0.010), tripolyphosphate (p = 0.046), cytidine-2’-monophosphate (p = 0.002), and inositol hexaphosphate (p = 0.003) in the presence of red LED, and for cysteamine-S-phosphate (p = 0.004) and phosphonic acid (p<0.001) when exposed to white LEDs. Affinity to D-mannose-1-phosphate was impaired under both red (p<0.001) and white (p = 0.019) LEDs compared with dark conditions.

### Impact of selected wavelengths on the energy source utilization pattern by *P*. sp. DR 5–09

Reduced substrate utilization was the most pronounced impact of light exposure imposing restrictions on major pathways. The strongest response was found for blue LED compared with dark incubation, with a total of 140 substrates negatively affected. In the presence of polychromatic LEDs, utilization of 42 compounds was also restricted. Except for sorbic acid, all of these were among those limited by blue light. Likewise, red LED negatively affected the metabolism of 21 substrates. Of these, respiration of 11 substrates was reduced by all three light regimes compared with dark incubation. Blue and red LED exposure lowered the utilization of eight substrates compared with the control. Interestingly, Tween 80 was only restricted in the presence of blue, but not white LEDs.

Considering the 379 compounds or conditions, there was no consensus on directionality in compounds’ utilization under the various light regimes. In other words, no individual light regiment was consistent in increasing or decreasing utilization of all compounds that were differentially regulated (either displaying increased or decreased use compared to dark conditions). *Pseudomonas* sp. DR 5–09 responded to blue and white LEDs by increased utilization of adenosine, propionic acid, D-citramalic acid, pectin, p-hydroxy-phenylacetic acid, D-arabitol, D-galactonic acid-γ-lactone, uridine, m-tartaric acid, and parabanic acid. Meanwhile, sorbic acid and D-galactose were metabolized to a larger extent in the presence of red and white LEDs. As opposed to the dark incubation, monochromatic blue LED supported the respiration of α-keto-glutaric acid, acetic acid, putrescine, sodium formate, dihydroxy-acetone, alanine-glycine, and bromo-succinic acid, while polychromatic white LEDs promoted the metabolism of succinic acid, D-glucosamine, D,L-lipoamide, glycine-glutamine, succinamic acid, and N-phthaloyl-L-glutamic acid.

Interestingly, four compounds showed contrasting utilization patterns when the bacterium was exposed to different light spectra. In the presence of blue and white LEDs, utilization of inositol hexaphosphate was reduced, but during red LED incubation it was promoted. Likewise, respiration decreased in the presence of capric acid, malonic acid, and cysteamine-S-phosphate upon exposure to blue light, but increased in response to red LEDs.

An overlay of involvement of the test substrates in microbial KEGG-pathway maps with affecting light conditions (see S3 Fig) revealed that substrates mainly affected by blue light only were highly represented in maps reflecting energy metabolism (01200 –Carbon metabolism, 01120—Microbial metabolism in diverse environments), but also more specific pathways like 00240 (Pyrimidine metabolism) and 00270 (Cysteine and methionine metabolism). Furthermore, we detected a cluster of maps affected only by blue LED representing ‘Biosynthesis of alkaloids derived from terpenoid and polyketide’ and ‘Biosynthesis of terpenoids and steroids’ (01066 and 01062). Closely related, but comprising phenylalanine influenced by non-blue LED treatments, was a cluster of ‘Biosynthesis of alkaloids derived from the shikimate pathway’ (01063), ‘Biosynthesis of phenylpropanoids’ (01061), ‘Biosynthesis of plant hormones’ (01070), ‘Biosynthesis of alkaloids derived from ornithine, lysine, and nicotinic acid’ (01064), ‘Biosynthesis of alkaloids derived from histidine and purine’ (01065), and ‘Glyoxylate and dicarboxylate metabolism’ (00630).

**Fig 6:**
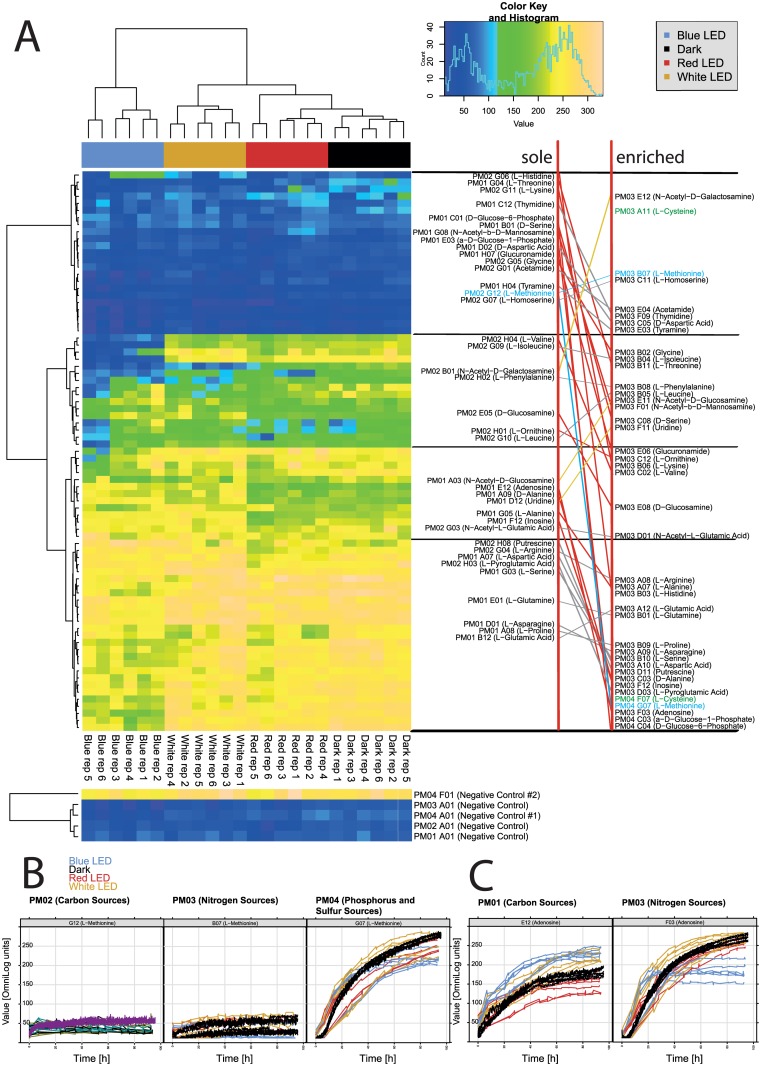
Respiration patterns of *Pseudomonas* sp DR 5–09 when compounds were provided as sole nutrient sources or enriched with sodium succinate and ferric citrate. (A) Heatmap of utilization of selected sole and enriched substrates by *Pseudomonas* sp. DR 5–09. The legend (upper corner to the left) explains the color code from blue to green, while yellow shades indicate low, moderate, and high substrate utilization, assessed as extinction (Omnilog values). The histogram describes the frequency of utilization. Grey and red links show compounds with non- significant and significant differences, respectively, when offered as a sole or enriched compound. Yellow links show compounds that decreased respiratory activity when offered as enriched compound compared with sole nutrient source. (B and C) Comparison of respiration curves under all four treatments with dark (black line), blue, red, and white LED treatments (blue, red, and yellow lines, respectively) on (B) methionine and (C) adenosine.

A direct comparison of respiration patterns of *Pseudomonas* sp. DR 5–09 for substrates provided both as sole compounds and enriched with carbon sources (sodium succinate, ferric citrate) in the presence of different light regimes is shown in Fig 6. There was no general pattern of substrate utilization when *Pseudomonas* sp. DR 5–09 was incubated in enriched suspensions on N, P, and S panels. However, for all light treatments, respiration in the S-deprived negative control wells was consistently higher than in the negative controls of the C, N, and P panels.

The utilization of thymidine, D-aspartic acid, acetamide, tyramine, and L-homoserine was not affected, irrespective of the nutritional conditions and light regime (Fig 6A, upper part, grey lines). Compounds that yielded low respiratory rates when provided as a sole substrate, such as the amino acids histidine, L-threonine, L-lysine, D-serine, L-methionine, and glycine, the amide glucuronamide, and the phosphorylated sugars D-glucose-6-phosphate and α-D-glucose-1-phosphate, were utilized substantially under all light regimes when enriched. In contrast, utilization of N-acetyl-D-galactosamine was lower in the enriched cultures than when provided as the sole source, and significant differences in utilization between exposure to darkness and blue LED were detected (Fig 6A, upper part, yellow line). Apart from histidine, blue LED exposure lowered the respiration of all those substrates.

Certain compounds were resistant to both carbon source enrichment and light regime (N-acetyl-L-glutamic acid, L-aspartic acid, L-pyroglutamic acid, L-glutamine, L-asparagine) (grey lines in Fig 6A). Respiration of L-alanine in the presence of blue and white LEDs was high when it was provided as the sole substrate and utilization was not significantly affected by enrichment. However, when exposed to red LED and darkness, L-alanine provided as a sole substrate was poorly used, while when enriched it was utilized at a high rate. The same pattern was found for L-arginine, inosine, and putrescine. L-glutamic acid as a sole source showed higher utilization under blue and dark conditions whereas respiration was diminished under both white and red LED. When *Pseudomonas* sp. DR 5–09 was incubated with enriched substrate, respiration was generally high and no effect of light treatment was detectable.

Two substrates were of specific interest, namely L-methionine and L-cysteine. When provided as a sole carbon source or as an enriched source on the N panel, no utilization was observed, irrespective of the light regime (Fig 6A, highlighted in green and blue). However, high utilization was found when L-methionine was tested as an enriched S substrate under white LED (p<0.001), but blue light treatment resulted in slightly diminished respiration (Fig 6A and 6B). L-cysteine only occurred as an enriched substrate in the N and S panels. Interestingly, *Pseudomonas* sp. DR 5–09 utilized L-cysteine when provided as an S substrate, but not when offered as an N substrate. In addition, light regime affected the utilization of L-cysteine by *Pseudomonas* sp. DR 5–09 on the S panel similarly to the utilization of L-methionine. Adenosine showed a similar pattern in response to substrate richness and light regime (Fig 6C).

As Fig 6A illustrates, blue LED also had a detrimental impact on utilization of L-valine and L-isoleucine provided as a sole carbon source. While enrichment generally resulted in higher respiration, exposure to blue light was even more pronounced with L-Isoleucine than L-valine.

Glycine and L-threonine displayed contrasting utilization patterns when enriched. While all three light regimes prevented utilization of glycine when provided as a sole substrate, it was used when provided to *Pseudomonas* sp. DR 5–09 as an N source and incubated under white and red LEDs or dark conditions. For glycine, the detrimental effect of blue LED persisted and white LED incubation favored its utilization under enriched conditions. In contrast, blue and white LED had a detrimental effect on bacterial utilization of L-threonine under enriched conditions.

## Discussion

For microbial leaf colonizers, light spectrum, intensity, and heat adaptation are important assets, but equally essential is the ability of the microorganism to cope with oscillating availability of water and nutrients, temperature, humidity, oxidative stress, and changing light spectra [1–4]. The discussion first covers technical details of modification of the Biolog Phenotype MicroArray^™^ technique and the newly developed protocol, and then positions key findings in the context of current knowledge about photobiology of non-phototrophic bacteria. It then outlines implications for greenhouse plant production and examines the potential for development of novel antimicrobials or adjuvant compounds deploying light-receptor signaling cascades modifying bacterial susceptibility to antimicrobials.

### Modification of the Biolog Phenotype MicroArray^™^ technique

In order to disentangle phyllosphere bacteria × light interactions regarding substrate utilization, we developed a new protocol modifying the Biolog Phenotype MicroArray^™^ technique, which eliminates effects from both host (plant) and surrounding microbiome normally present on plant surfaces. This study contributes to the understanding of interactions between light spectrum and bacterial phyllosphere colonizers by (i) facilitating assessment of direct impacts of light treatments by modification of the Biolog Phenotype MicroArray^™^ technique, (ii) providing an optimized protocol for measuring the impact of light treatment on respiratory profiles of non-phototrophic gram-negative bacteria, and (iii) presenting comprehensive data on the impact of different visible light spectra on respiratory profiles of the leaf colonizer *Pseudomonas* sp. DR 5–09.

Phenotypic assays are commonly single to few-endpoint assessments describing phenotypic implications of changes in growth behavior, cell shape, lifestyle (biofilm formation, swarming activity, colony color, etc.), gene expression, or modified gene products under varying external conditions. Comparisons of different mutants (deletions, aberrations, SNPs, modifications of promotor regions, and others) tested under different stressors often compose the panels. For example, Wu et al. [23] developed an assay to study the impact of different light spectra on swarming motility of the plant pathogenic bacterium *P*. *syringae*, using image analysis. However, few phenotypic assays have been established for studying the impact of light spectra on phyllosphere colonizers. In this study, we established a phenotypic assay based on the respiratory behavior towards 379 substrates and conditions when exposed to different light spectra, which permitted tracking of metabolic pathways that are potentially affected by light. This is the first report of such extended use of the Omnilog platform. In the following, we discuss technical difficulties and pitfalls arising from this extension.

The existing system for PM assays, consisting of pre-fabricated microtiter plates with different nutritional and environmental conditions allowing kinetic analysis through an incubator reader, is designed for dark incubation only. At present, treatments with and manipulations of the light spectrum have to be performed manually, in a constant chamber with lined light cabinets. Gnotobiotic assays can be challenging in such settings. However, by using a self-adhesive covering film to prevent contamination under ambient experimental conditions, we were able to overcome the contamination risk. Under long-term incubation, sealing of the plates also reduces volume losses caused by evaporation. However, sealing may influence the oxygen status negatively and thus might influence substrate utilization by aerobes. Visual assessment of respiration kinetics (xy-plots in S2 Table) revealed high reproducibility of the assay, although the read-outs were performed manually every six hours during a 96-h period.

The set-up designed here should be regarded as an experimental blueprint. However, we are very aware of the fact that manual read-outs every six to eight hours, over three days or longer, impose a critical workload on staff and high demands on instrumentation. Furthermore, the manual read-out mode generates a large set of single data files that have to be carefully integrated, requiring additional sophisticated data management steps before starting the actual data analysis. For routine assessment of light impact, we would thus aim for a semi-automated incubation and monitoring system to manage the light treatments. This would include major structural changes to the currently available Omnilog reader, but in our view such changes are highly desirable, as the panel system is not designed for incubation studies with different light conditions.

### Effect of light on non-phototrophic phyllospheric bacteria

Despite the overruling impact of light on all organisms, only a few studies consider the direct interactions between phyllosphere-colonizing bacteria and non-UV light [15, 23, 36–38]. In this study we showed that (i) different visible light spectra directly change the respiratory profile of non-phototrophic leaf colonizing bacteria, (ii) different light wavelengths have different effects on the metabolic profile of the test strain in single treatments, but also indicate synergistic effects when applied as a mixture in white LED, and (iii) carbohydrate supplements, and thus the nutritional status of organisms, may modify the impact of light regarding ability to respire on certain substrates.

### Impact of light on respiratory profile

Our results provide a strong indication that different visible light spectra may directly change the respiratory profile of non-phototrophic leaf-colonizing bacteria. Although little is known about bacteria × light interactions in plant science, some knowledge is available from the medical field concerning species of the same genus. Responses of various medically important microorganisms to different light spectra, mainly focusing on different bands of blue light (405 nm, 450–470 nm), have been described [36, 39–43].

### Different wavelength revealed different effects

In the presence of blue LED, utilization of certain substrates was impeded. The sensitivity of non-phototrophic bacteria to light and the impact of blue light on bacterial lifestyle has been discussed in various fields [19, 22, 44]. *Pseudomonas* sp. DR 5–09 was first sequenced in 2015 [45] and little is known about its molecular make-up of light sensing receptor proteins. Such information is essential in order to understand in depth how blue light affects metabolic pathways in *Pseudomonas* sp. DR 5–09. However, the sensitivity of the test strain *Pseudomonas* sp. DR 5–09 to blue light in the present study indicates the presence of blue light receptor proteins. Indeed, a putative blue light receptor in *Pseudomonas* sp. DR 5–09 shows high similarity with that reported for *Pseudomonas syringae* (GenBank accession number WP 0592965543), *P*. *fluorescens* (GenBank accession number WP014340143) and *P*. *moraviensis* (GenBank accession number WP065615803) (S4 Fig). In order to further validate the blue light responses detected in this study, future studies should include blue light receptor deletion mutants of *Pseudomonas*.

Glucose, fructose, sucrose, and galactose are dominant carbohydrates on the leaf surface [46, 47]. Interestingly, no deviations between blue light and dark incubation were found for these compounds. However, D-galactose was the only carbon source with impaired utilization under red LED compared with dark incubation. Most striking was the considerable impact of blue LED on utilization of P substrates, which suggests that several crucial pathways are hampered due to blue light exposure (S3 Fig).

As the deleterious impact of blue light on non-photosynthetic bacteria is well established (see above), the considerable clustering of blue light responses was expected. Further analysis of inhibited pathways is needed in forthcoming studies. On the basis of previous studies, however, it is surprising that respiration was favored in the presence of some substrates, especially when incubated under blue LEDs. In particular, this finding deserves more attention in the future when deciphering affected pathways and signaling cascades. We reject the notion that blue light caused a general growth impairment of *Pseudomonas* sp. DR 5–09 and that this would explain the different substrate utilization profile detected for this light treatment. First, some substrates were utilized at a higher rate than the dark control, which would not be expected if the bacterium was suffering from general growth impairment. Second, analysis of growth in batch culture and enumeration of viable population density demonstrated that once cells reach stationary phase (within 11 h) there is no difference in population densities between the four light conditions (S5 Fig). Cells under blue light had significantly lower population densities at the beginning of log phase than cells grown in the dark or under white or red LEDs, but this difference was slight and we do not consider it biologically relevant enough to explain any of the observed difference in substrate utilization profiles (7.38 log cfu × ml^-1^ vs 7.70 to 7.76 log cfu × ml^-1^ growth medium). When considering the growth of *Pseudomonas* under different light regimen it is clear that there is an interaction taking place between light and nutrient source. While *Pseudomonas* sp. DR 5–09 when grown in continuous blue light experienced minimal to no growth impairment while grown in TSB (S5 Fig), it could be observed that blue and white light delayed the onset of log phase when cells were cultured in 10 mM D-mannose (S6 Fig) compared to cells under red LED or in darkness, with bacteria kept under blue light exhibiting the longest lag phase of all treatments. However, it is important to note that blue light did not kill *Pseudomonas* DR 5–09 cells. Furthermore, considering that bacterial utilization of D-mannose was similar under blue LED light (S2 Fig) and the dark control, there is a distinct possibility that cells kept under blue LED light have a higher per cell utilization of this substrate, based on the lower population sizes of DR 5–09 grown in D-mannose in blue light, compare to cells kept dark.

### Nutritional status may affect ability to use certain substrates

In the present study, *Pseudomonas* sp. DR 5–09 displayed normal growth in minimal medium supplemented with succinate. This compound has previously been suggested as a preferred carbon source for *P*. *aeruginosa* on the basis of its position in the citrate cycle. Li and Lu [48] propose that preferred carbon sources (succinate, L-aspartate, glycerol, L-glutamate, L-asparagine, fumarate, α-ketoglutarate, L-glutamine) of *P*. *aeruginosa* are closely related to the TCA cycle. For our test strain, *Pseudomonas* sp. DR 5–09, L-aspartic acid, L-arginine, putrescine, L-pyroglutamic acid, L-serine, L-glutamine, L-asparagine, L-proline, and L-glutamic acid were respired under both sole and enriched conditions to a high extent. This pattern is different from that proposed for *P*. *aeruginosa* and the selection of compounds also contrasted with the previously described TCA cycle relationship.

### Implications for greenhouse plant production

To cope with environmental changes occurring in the phyllosphere, successful colonizers must be sensitive to fluctuations fundamentally affecting their living conditions. This was demonstrated with respect to light and nutritional conditions in the present study with *Pseudomonas* sp. DR 5–09. However, the pattern of nutrient utilization cannot be explained solely as a consequence of a certain electromagnetic spectrum or nutrient availability. As initially mentioned, target substrate concentrations differ between the four panels (C>N>P>S) and the nutritional conditions are in general richer in test panels for N, P, and S than for C panels, where the energy source is provided as a sole source. Low utilization of substrates provided as sole sources may partly be explained by a lack of uptake mechanisms or imbalance of nutrients essential for their uptake. Davidson et al. [49] provide a general overview of prokaryotic transporters. In the case of ABC importers, which are found in prokaryotes only, a substrate-binding protein (SBP) as fifth domain is part of the functional unit. Advances in structural protein biochemistry and resolution have facilitated a new classification of substrates into six clusters, based on features of their three-dimensional structure [50]. With recent findings from comparative genomics, Maqbool et al. [51] illustrated how SBP forms the key determinant of the substrate specificity and high affinity of ABC uptake systems. The presence of ammonia as a sole N source under sole substrate conditions may also repress the uptake of certain compounds [48]. These two reasons may also apply to low utilization under enriched substrate conditions. However, carbon catabolite repression has been reported for various gram-negative bacteria, among these members of the Pseudomonadaceae family, e.g., *P*. *aeruginosa* and *P*. *putida*. Catabolism and repression of sole and enriched substrates are entangled. For example, using the same experimental platform, Li and Lu [48] studied control of C and N utilization by *P*. *aeruginosa* and found that some N compounds could be used by *P*. *aeruginosa* as C sources in an enriched environment, although compromised under sole conditions, which may be supported by our findings. Their study also demonstrated a link to the two-component system CbrAB, which is important for adaptation to environmental changes [52] and thus vital for epiphytic conditions, such as nutrient availability, osmolarity, and osmotic conditions.

Use of artificial lighting and the potential to apply targeted light wavelengths to plants on a long-term or continuous basis is an important tool in intensive plant production systems, especially in greenhouses and controlled environment horticulture, to improve plant biomass formation, plant shape, and formation of plant bioactive compounds. Studies on impact of artificial lighting on plant productivity that include organisms relevant to plant health are rare in the area of plant science. Phyllosphere bacteria can have significant impact on plant productivity, either by bolstering beneficial outcomes or by contributing to detrimental outcomes for the host plant. If light regimes are significantly altered on a large scale in production systems, for example by using monochromatic LEDs, phyllosphere residents may be significantly affected. Unless light as a factor in and of itself is explored regarding its impacts on non-phototrophic bacteria, it is nearly impossible to judge whether those bacteria are directly impacted by light and/or indirectly by effects of host plant responses to the light regime.

As mentioned above, it is not light spectrum alone, but also nutritional factors that affect microbial phenotypic responses [53, 54]. Our results indicate that complex nutrient sources are likely to aggravate the influence of blue light on respiration of *Pseudomonas* sp. DR 5–09. As mentioned earlier, information on the impact of light, especially blue light, on phyllosphere-colonizing bacteria is scarce. Alsanius et al. [55] studied the leaf microbiota of greenhouse-grown sunflowers when exposed to red and a combination of red and blue LEDs compared with high pressure sodium lamps using a metagenomic approach, and found no difference in the bacterial phyllosphere community structure between the three treatments. However, as (i) no treatment with sole blue LED was included and (ii) these data reflect community structures based on 16S rDNA from cells, irrespective of their viability status, i.e. happily living and proliferating, suffering, or even dead, the conclusion on lack of impact of LED light regimes on the phyllosphere microbiota is not adequate. Wu et al. [23] reported that blue light positively regulates the swarming activity of *P*. *syringae*, whereas red and far-red bands repress its motility. In contrast, Rio-Alvarez et al. [15] noted that blue and white light inhibits motility, as well as attachment to tomato leaves. It is tempting to assume that white light reactions comprise effects observed under blue light, as a blue band is part of polychromatic white light. However, the results from the present study indicate that the interactions are more complex and that respiratory behavior under white LED is not always upscaled or downscaled in the same direction in the presence of blue LED. Together with knowledge about different receptors for different light wavelengths present also in non-phototrophic bacteria [18, 19], our experimental approach could form the starting point for systematic discovery of pathways stimulated by different light wavelengths, resulting in changes in lifestyle or display of fine-tuned substrate usage patterns.

### Potential for development of novel antimicrobials or adjuvant compounds

Blue light has been used in medical treatments, for example to kill *Pseudomonas aeruginosa*, *Staphylococcus aureus*, *Proponibacterium acnes*, *Escherichia coli* and *Porphyromonas gingialis* when exposed to various energy fluxes and time intervals, and dose-response curves have been established [36, 39–43]. However, blue light is not yet considered a widely applicable alternative or supportive antimicrobial treatment [56, 57]. As reviewed by Yin et al. [57], many microbial cells are highly sensitive to blue light (400–470 nm). The known mode of action to date comprises photoexcitation of naturally occurring porphyrins, which act as endogenous photosensitizers [39]. Beside these deleterious effects, many studies report indirect impacts on microbial cell survival due to the impact of light on lifestyle and metabolic changes. For *Pseudomonas*, an impact of light on swarming behavior has been shown [23], while reception of light by bacteriophytochromes and light, oxygen, or voltage sensing (LOV) as well as the downstream signaling cascade, has revealed that LOV-containing histidine kinase (LOV-HK) can act as a repressor of the BphP1-mediated blue-light response. Together with findings in [15], quite a detailed picture of light regulation of motility in *Pseudomonas* is emerging. Broadening the scope, general aspects of photoregulation in prokaryotes [13, 14] indicate that light-dependent gene regulation is widely distributed in non-phototrophic bacteria such as *Listeria* [22] and *Acinetobacter* [24]. Considering the importance of being able to monitor changing environmental conditions, ability to perceive and distinguish between different visible wavelengths has to be acknowledged as a major feature driving the biogeography of species and, indirectly by changing lifestyles and metabolic activities, the structure and composition of microbial communities.

In contrast to clinical contexts, where therapeutic interventions seldom take longer than a few minutes, supplementary light in plant production is usually applied as long-range to constant treatments (12–16 h per day) during the entire production phase, which may take several months to compensate for low light conditions. Although the experimental conditions in our study were not directly comparable to those used in medical contexts, we were able to validate certain effects of visible blue light on metabolism of the test strain. Considering the ability of *Pseudomonas* to develop biofilm, one could argue that, due to lack of other strategies such as pigment formation, blue light perception and responses comprising changes in lifestyle are just another method to ensure survival and avoid damage by shortwave light. As shown previously [10], differential survival of solitary and aggregated bacterials promotes aggregate formation on leaf surfaces. We wish to emphasize that the present study addressed the impact of different light spectra relevant for plant growth rather than killing of bacteria. Therefore, photosynthetically usable blue and red light bands with reasonable intensities and duration were employed, considering later application of the findings in terms of supplementary lighting in plant production [58, 59]. However, if the deleterious molecular impact of different light regimes in microbial cells (production of reactive oxygen species (ROS)) is mediated by naturally occurring photosensitizers [56, 57] with regards to both immediate microbial survival and long-term lifestyle changes, including changes in microbial communities, this might pave the way for novel strategies to fight bacterial infections. Treating bacteria with naturally occurring ability to sense light with newly developed photosensitizers could lower their sensitivity to antimicrobials to levels below critical thresholds.

### Outlook

Light spectrum can vary quite rapidly within the plant canopy, within minutes and hours. Upper leaves are exposed to the ambient light under natural conditions or under artificial overhead illumination, whereas lower leaves are subjected to different levels of shading, including green light spectrum, as well as sunfleck. The present findings indicate that phyllosphere microbiome × light interactions are very complex and that light properties need to be taken into account when controlling beneficial, neutral, and deleterious microbial effects. However, the responses portrayed here relate only to one strain of *Pseudomonas* sp. DR 5–09. Other bacterial genera and species need to be studied before general conclusions can be drawn. Analysis of phenotypic responses of bacteria to different light spectra is one step towards understanding abiotic and biotic phyllosphere interactions. The phenotypic array developed here produces data that can be used to identify pathways impacted by light and predict microbial responses to different light spectra.

## Supporting information

S1 Table Cover material tested for transmittance of light with different wavelength. (PDF)

S2 Table Substrate overlay on the four selected PM panels (PM1, PM2, PM3, PM4 according to Biolog Inc., Haywood, USA). Substrates and positions are displayed for each of the PM panels. (Abricot, light green, light purple and light yellow highlighted areas consider C, N, P and S sources, respectively. Negative controls are displayed on a grey (sole nutrient sources) or light blue (enriched nutrient sources) background.
(PDF)

S1 Fig Curve parameters. (TIF)

S2 Fig Heatmaps on maximum curve height (A), area under the curve (AUC), slope (mu) and lag-phase length (lambda) for utilization of carbon sources (PM 1 and 2), nitrogen sources (PM 3) as well as phosphorus and sulfur sources (PM 4) by *Pseudomonas* sp. DR 5–09 (strain 2) when incubated under dark or blue, red and white LED conditions. The curve parameters are explained in S1 Fig. Utilization was monitored during 96 h of incubation. The legend (upper corner to the left) explains the color code from blue to green, while yellow shades indicate low, moderate, and high substrate utilization, assessed as arbitrary Omnilog values. The histogram describes the frequency of maximum curve height, area under the curve, slope and lag-phase length reached for different substrates.
(PDF)

S3 Fig Mapping of substrates on microbial KEGG-pathways. KEGG-pathways are listed on the x-axis whereas directionalities of selected substrates in relation to light quality are shown on the y-axis. White or grey marked combinations show absence of mapping or mappings without effect, respectively. Clear blue marked combinations display mappings and impact by blue and other LED regimes whereas dark blue marked combinations only display mappings with an impact of blue LED exposure. Mangenta marked combinations consider mappings with an impact of white or red LED regimes.
(PDF)

S4 Fig Alignment of two putative blue light receptor of *Pseudomonas* sp. DR 5–09 (position 6 and 7) with *Pseudomonas* sp. DR 05–9 (GenBank accession number WP064593428) (position 5), *Pseudomonas syringae* (GenBank accession number WP 0592965543), *P*. *fluorescens* (GenBank accession number WP014340143) and *P*. *moraviensis* (GenBank accession number WP065615803) as reference sequences. (PDF)

S5 Fig Growth curves (A) and viable population levels (B) of *Pseudomonas* DR 5–09 under all light conditions. Each marker or bar represents the mean of four replicates and error bars denote standard deviation. Means with different letters are significantly different (p<0.05).
(TIF)

S6 Fig Viable population levels of *Pseudomonas* sp. DR 5–09 kept in M9 minimal medium supplemented with 10 mM D-mannose as a sole carbon source, at 20°C under all light conditions, for up to 96 hrs post inoculation. Each marker or bar represents the mean of four replicates and error bars denote standard deviation. Means with different letters are significantly different (p<0.05) and ns means no significant differences between the means.
(TIF)

S1 File Raw kinetic data including estimated curve parameters as.yml-file. (ZIP)

S2 File Curves of all replicates comparing each light condition to dark treatment during 96 h of incubation. The x-axis displays incubation time (h) whereas the y-axis shows the substrates’ utilization in omnilog units. xy-plots depicting the raw kinetics of all replicates comparing each light condition to dark treatment displayed high reproducibility. Results on utilization of C sources are shown on page 1, 5, 9 (PM 1) and page 2, 6, 10 (PM 2), and of N as well as P and S sources on page 3, 7, 11 (PM 3) and page 4, 8, 12 (PM 4). (black line: dark incubation, blue line: blue LED, red line: red LED, yellow line: white LED). Substrate names are above each individual plot (see also S2 Table).
(PDF)
