# Supplementary material for: Light spectrum modifies the utilization pattern of energy sources in Pseudomonas sp. DR 5-09
Source: PLoS One. 2017 Dec 21;12(12):e0189862. doi: 10.1371/journal.pone.0189862 (PMC5739431; doi:10.1371/journal.pone.0189862)
Supplement: S1 Table — (PDF) [file pone.0189862.s001.pdf]

Supplement Table S1: Cover material tested for transmittance of light with different wavelength

#	Material	Product number and provider	Comments
1	Plate lid for 96 well microtitre plate	PM panels, Biolog, Hayward, CA USA	Sterile, provided with PM panels; polyethylene; allows gas exchange; clear
2	Breath-easy sealing membrane	Cat.No. BEM-1, Diversified Biotech, USA; Sigma Aldrich Z380059-1PAK, St Louis, MO, USA	Sterile; polyurethane; adhesive; gas permeable; transparent
3	Titer-tops sealing film for microplates	Cat No T-Tops-100; Diversified Biotech; USA; Sigma Aldrich Z688630-1PAK, St Louis, MO, USA	Sterile; polyethylene; adhesive, clear
4	Sealing tape	T9571-100EA, Sigma Aldrich. St. Louis, MO, USA	Non sterile; acrylic, polyolefin; clear
5	Household plastic film 1	Microwave folie, 7340011372387, Coop Trading A/S, Albertslund, Denmark	Transparent, non sterile
6	Household plastic film 2	Toppits, Aromplastfolie, 4008871202727, Cofresco Frischhalteprodukte GmbH u Co KG, Minden, Germany	Transparent, non sterile
7	Greiner ViewSeal for 96 plates	Greiner Bio-one, 676070; Sigma Aldrich, Z617571-100EA, St Louis, MO, USA	Sterile; polyolefin; clear; adhesive
