# Supplementary figures and images for: Light spectrum modifies the utilization pattern of energy sources in Pseudomonas sp. DR 5-09
Source: PLoS One. 2017 Dec 21;12(12):e0189862. doi: 10.1371/journal.pone.0189862 (PMC5739431; doi:10.1371/journal.pone.0189862)

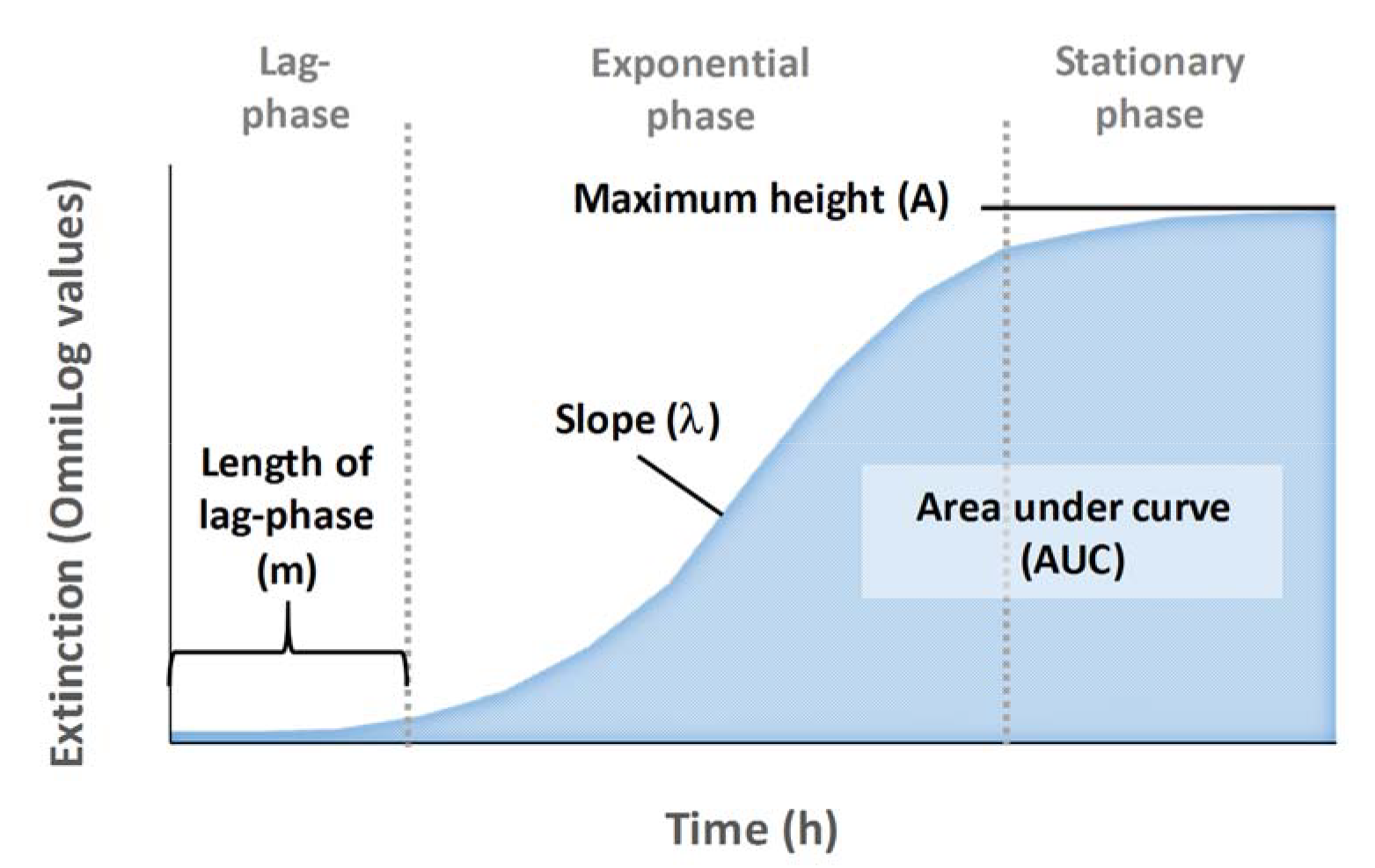

Supplement: S1 Fig — (TIF) [file pone.0189862.s003.tif]

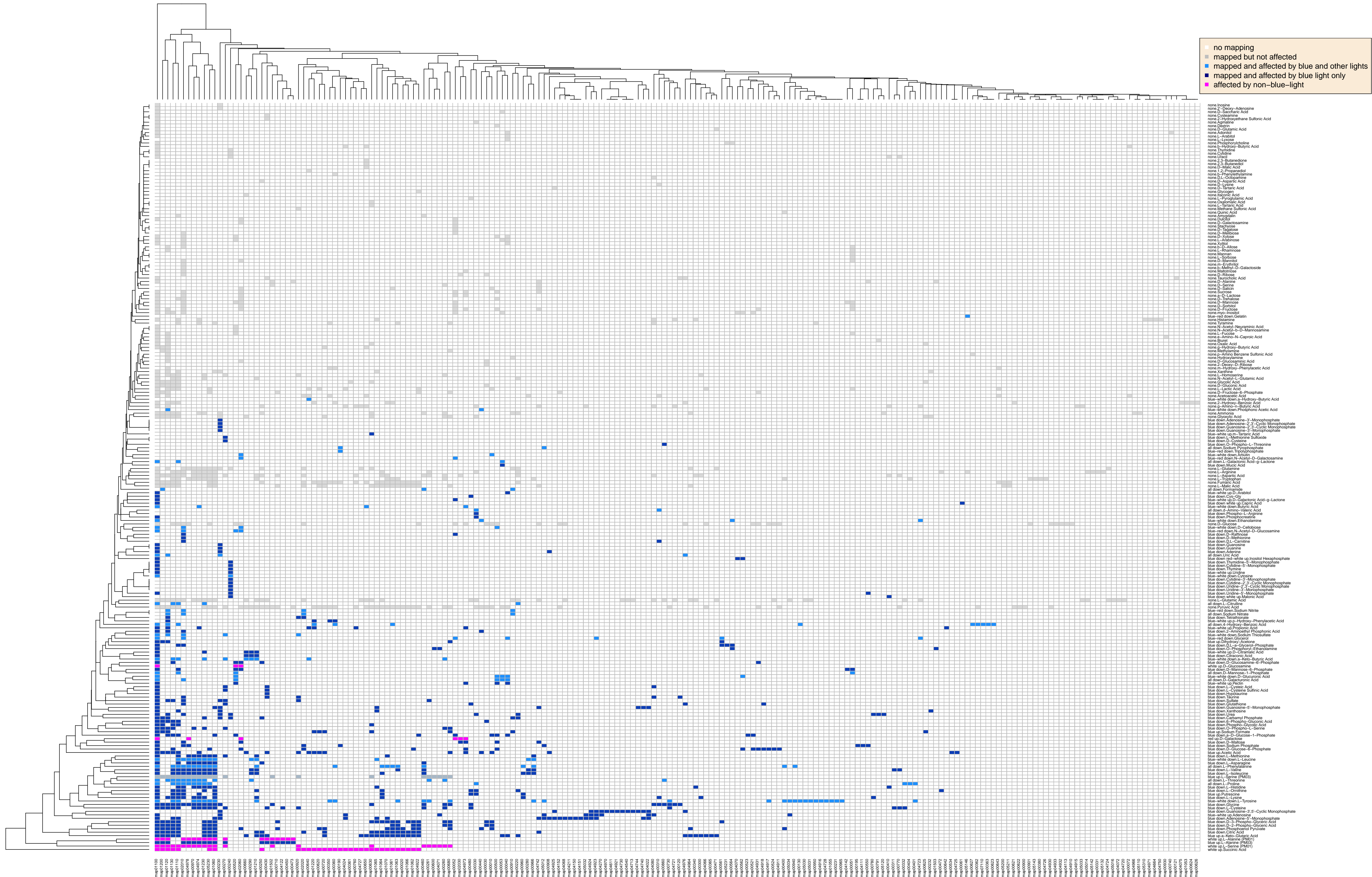

Supplement: S3 Fig — KEGG-pathways are listed on the x-axis whereas directionalities of selected substrates in relation to light quality are shown on the y-axis. White or grey marked combinations show absence of mapping or mappings without effect, respectively. Clear blue marked combinations display mappings and impact by blue and other LED regimes whereas dark blue marked combinations only display mappings with an impact of blue LED exposure. Mangenta marked combinations consider mappings with an impact of white or red LED regimes. (PDF) [file pone.0189862.s005.pdf]

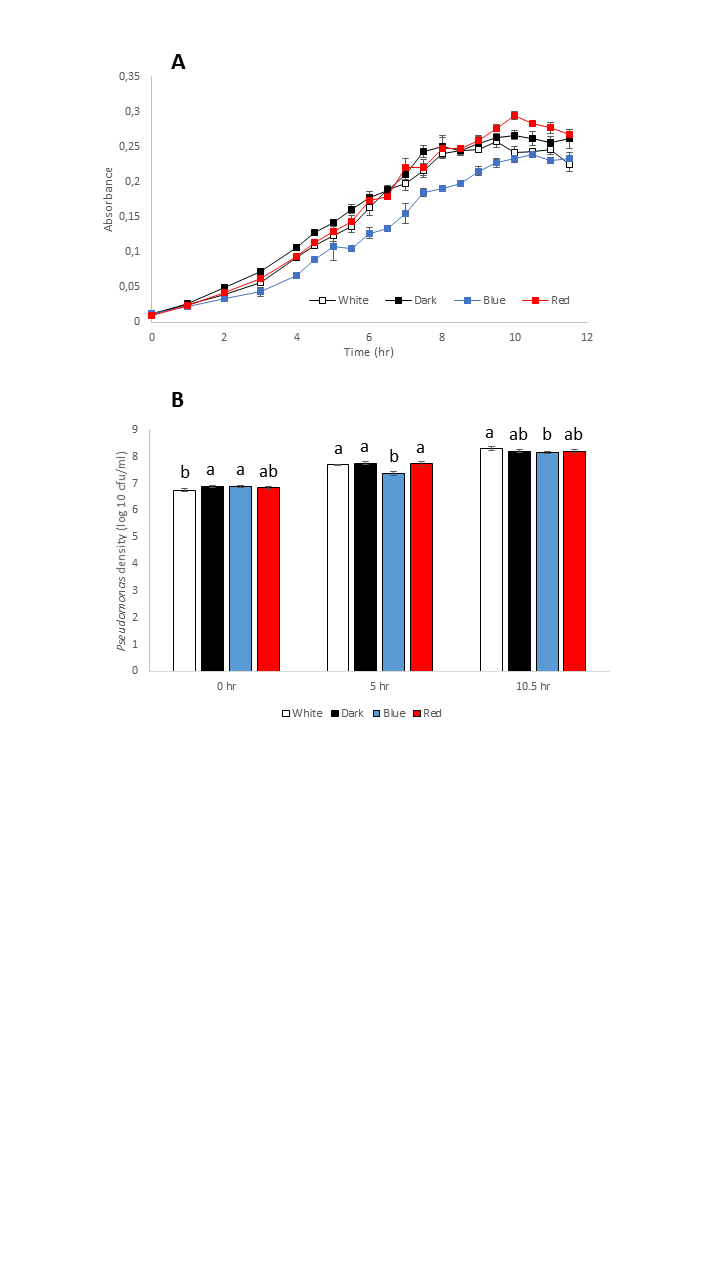

Supplement: S5 Fig — Each marker or bar represents the mean of four replicates and error bars denote standard deviation. Means with different letters are significantly different (p<0.05). (TIF) [file pone.0189862.s007.TIF]

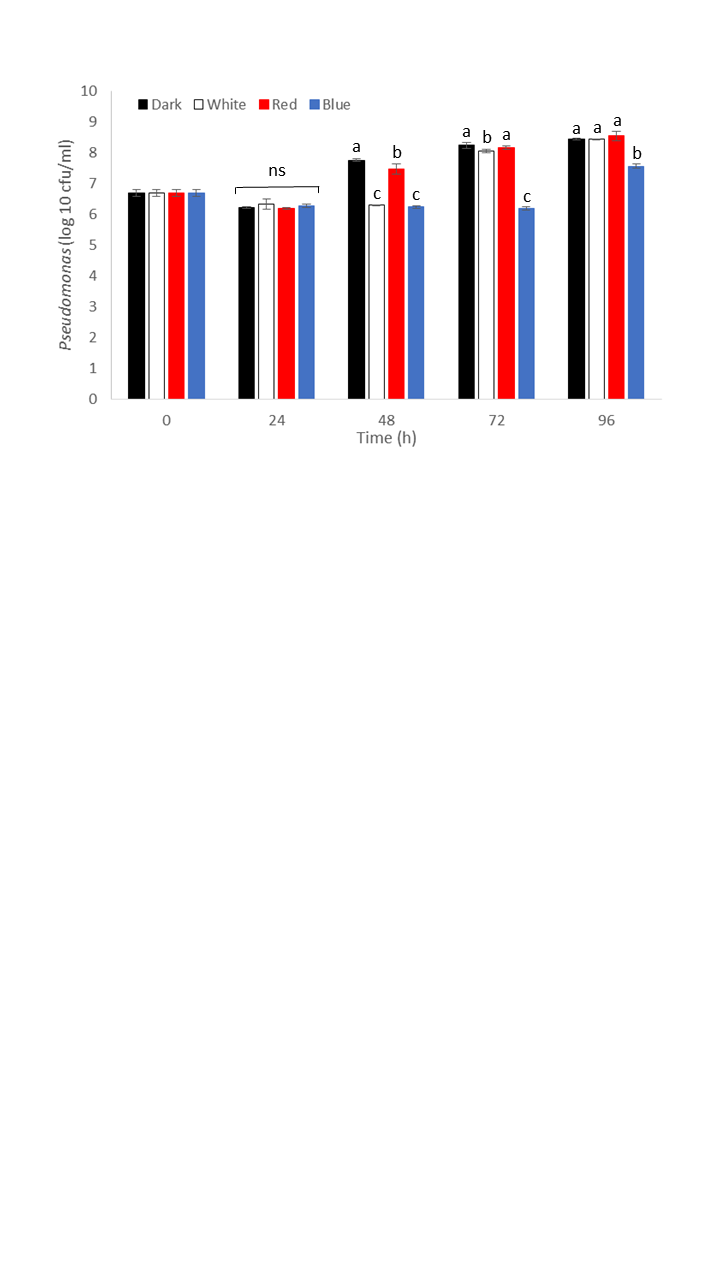

Supplement: S6 Fig — Each marker or bar represents the mean of four replicates and error bars denote standard deviation. Means with different letters are significantly different (p<0.05) and ns means no significant differences between the means. (TIF) [file pone.0189862.s008.TIF]
