# Supplementary material for: Light spectrum modifies the utilization pattern of energy sources in Pseudomonas sp. DR 5-09
Source: PLoS One. 2017 Dec 21;12(12):e0189862. doi: 10.1371/journal.pone.0189862 (PMC5739431; doi:10.1371/journal.pone.0189862)
Supplement: S2 Fig — The curve parameters are explained in S1 Fig. Utilization was monitored during 96 h of incubation. The legend (upper corner to the left) explains the color code from blue to green, while yellow shades indicate low, moderate, and high substrate utilization, assessed as arbitrary Omnilog values. The histogram describes the frequency of maximum curve height, area under the curve, slope and lag-phase length reached for different substrates. (PDF) [file pone.0189862.s004.pdf]

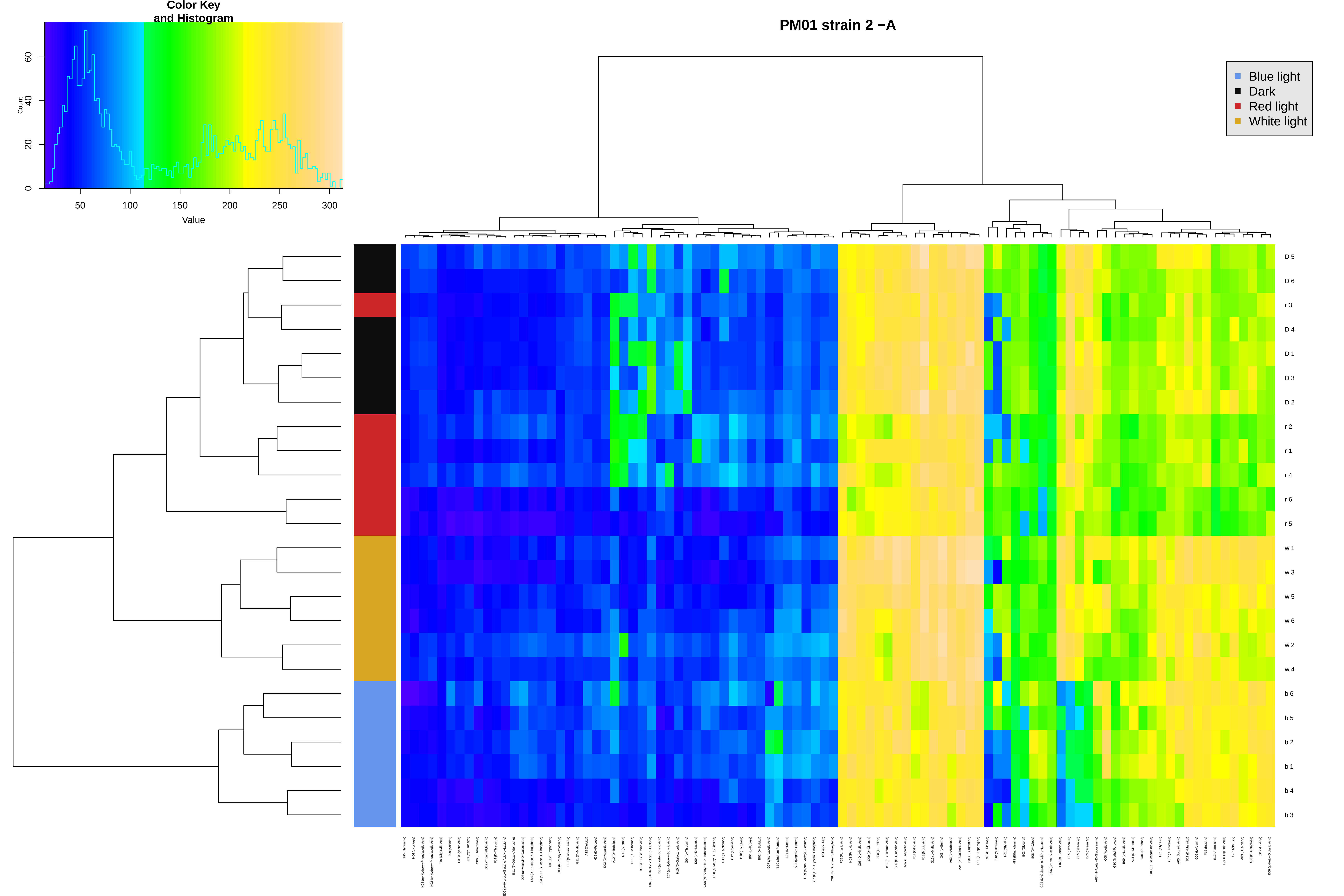
PM01 strain 2 −A
Color Key
and Histogram
Count
Value
0
20
40
60
50
100
150
200
250
300
Blue light
Dark
Red light
White light
D 5
D 6
r 3
D 4
D 1
D 3
D 2
r 2
r 1
r 4
r 6
r 5
w 1
w 3
w 5
w 6
w 2
w 4
b 6
b 5
b 2
b 1
b 4
b 3

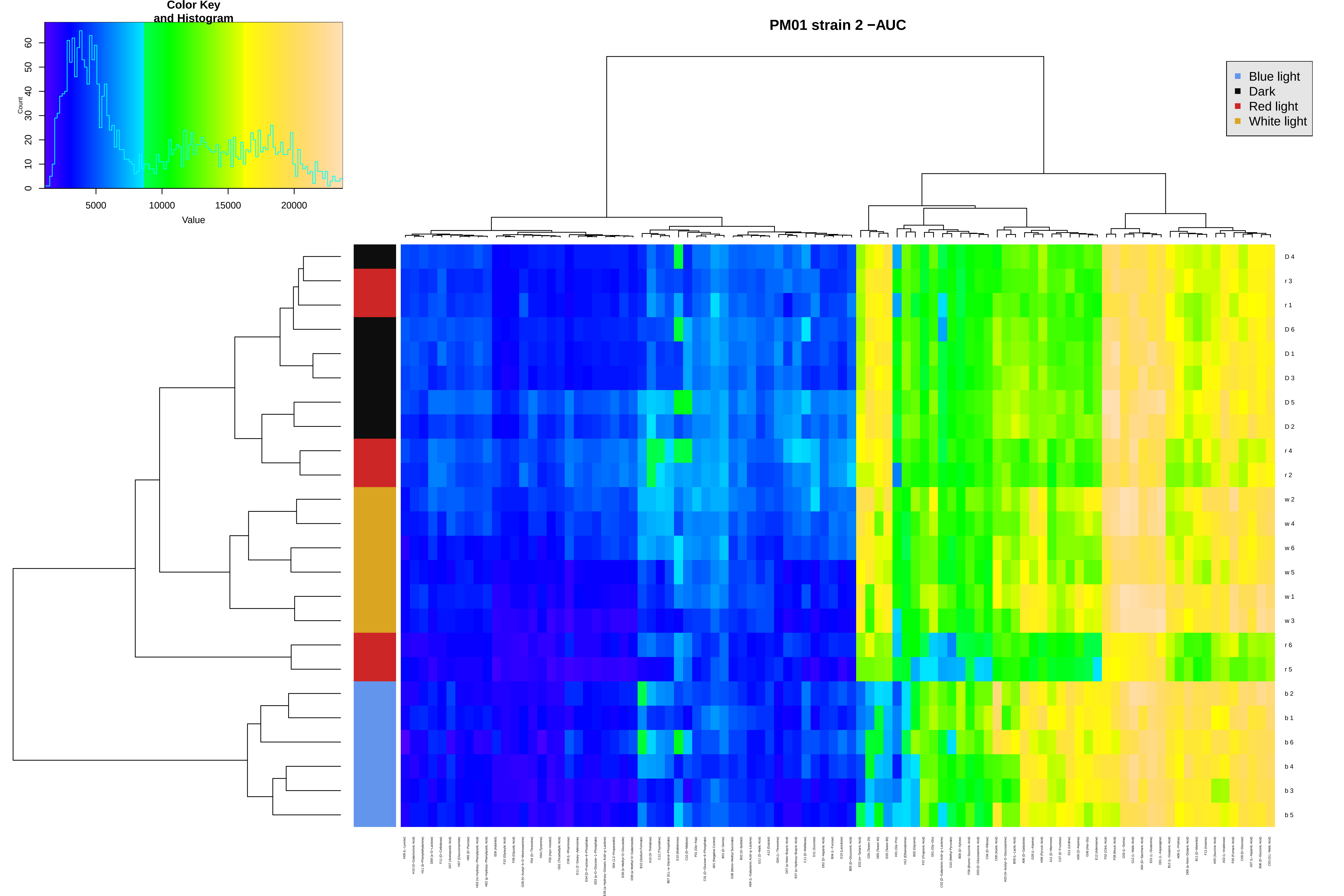

Color Key and Histogram
Count
Value
0
10
20
30
40
50
60
5000
10000
15000
20000
PM01 strain 2 −AUC
Blue light
Dark
Red light
White light
D 4
r 3
r 1
D 6
D 1
D 3
D 5
D 2
r 4
r 2
w 2
w 4
w 6
w 5
w 1
w 3
r 6
r 5
b 2
b 1
b 6
b 4
b 3
b 5

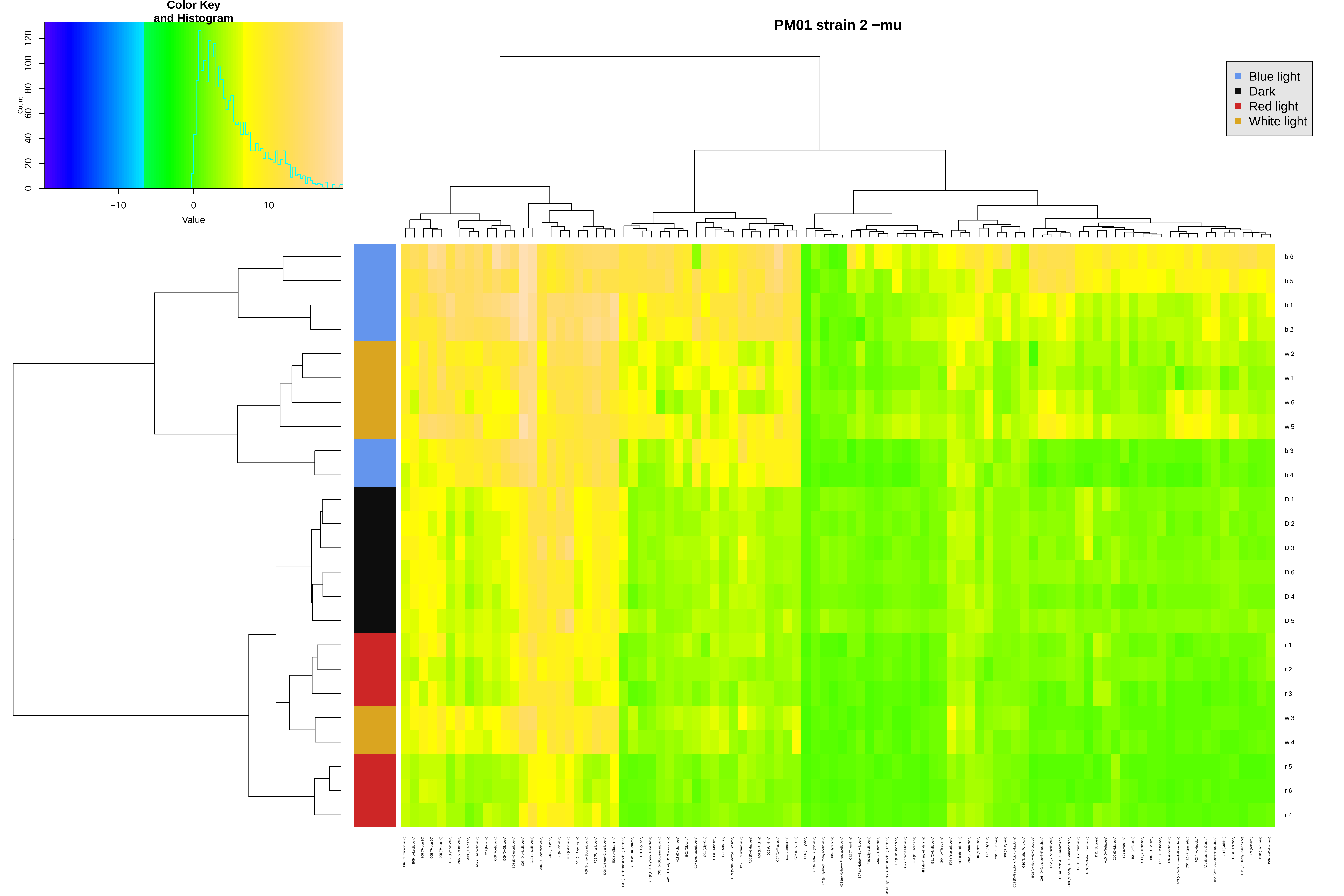
Color Key
and Histogram
Count
Value
−10
0
10
0
20
40
60
80
100
120
PM01 strain 2 −mu
Blue light
Dark
Red light
White light
b 6
b 5
b 1
b 2
w 2
w 1
w 6
w 5
b 3
b 4
D 1
D 2
D 3
D 6
D 4
D 5
r 1
r 2
r 3
w 3
w 4
r 5
r 6
r 4

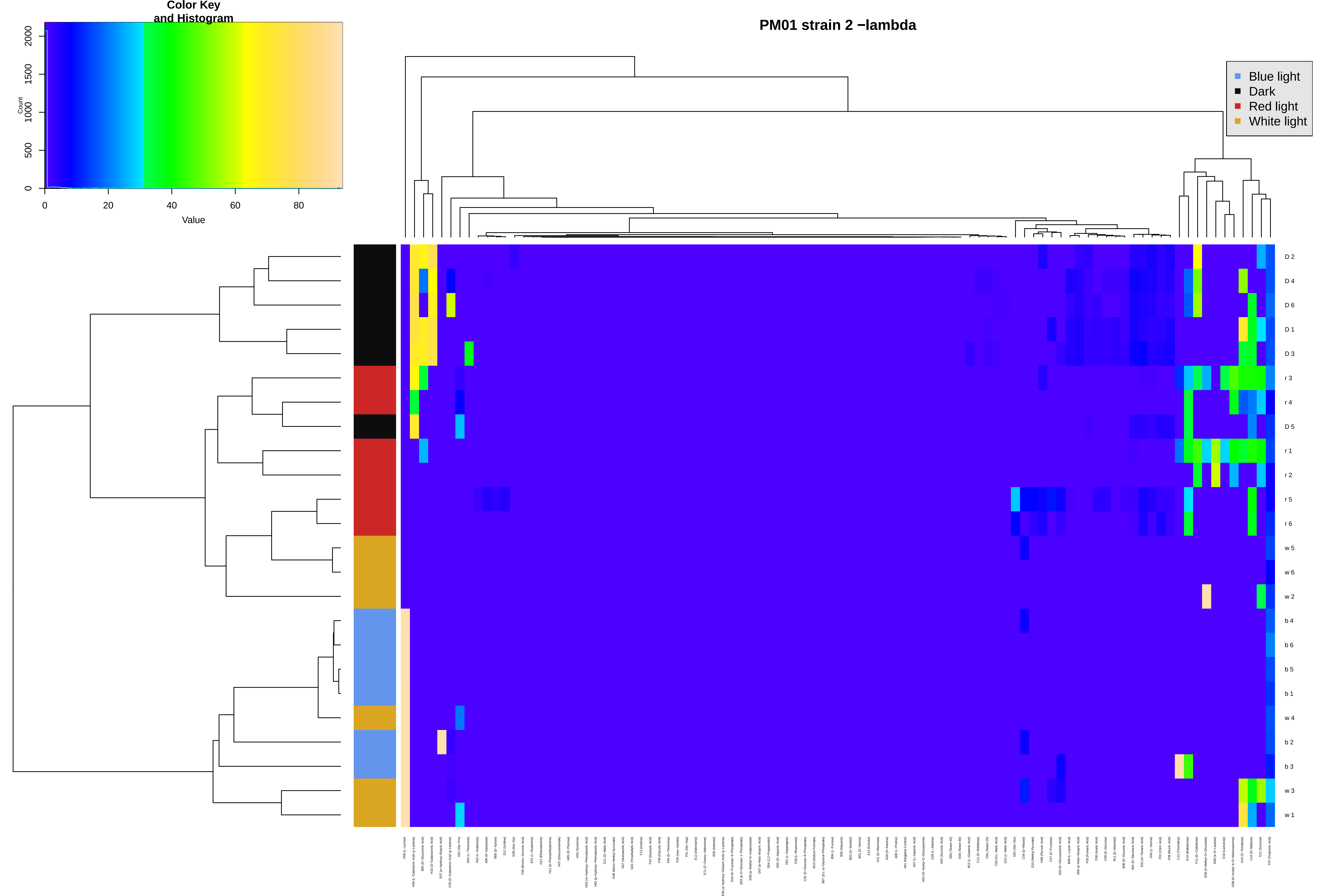
Color Key
and Histogram
Count
0
500
1000
1500
2000
0
20
40
60
80
Value
PM01 strain 2 −lambda
Blue light
Dark
Red light
White light
D 2
D 4
D 6
D 1
D 3
r 3
r 4
D 5
r 1
r 2
r 5
r 6
w 5
w 6
w 2
b 4
b 6
b 5
b 1
w 4
b 2
b 3
w 3
w 1

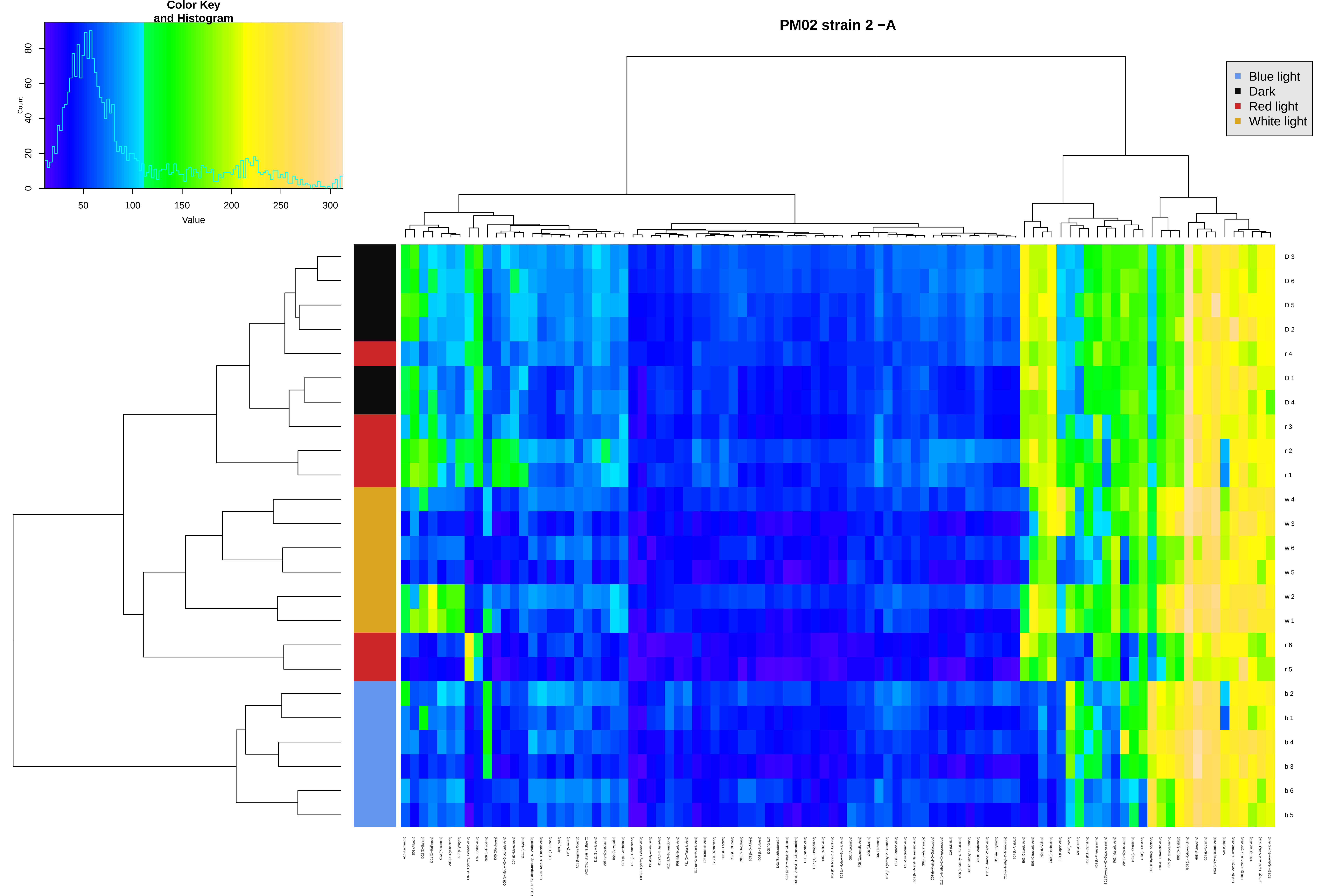
Color Key
and Histogram
Count
0
20
40
60
80
50
100
150
200
250
300
Value
PM02 strain 2 −A
Blue light
Dark
Red light
White light
D 3
D 6
D 5
D 2
r 4
D 1
D 4
r 3
r 2
r 1
w 4
w 3
w 6
w 5
w 2
w 1
r 6
r 5
b 2
b 1
b 4
b 3
b 6
b 5

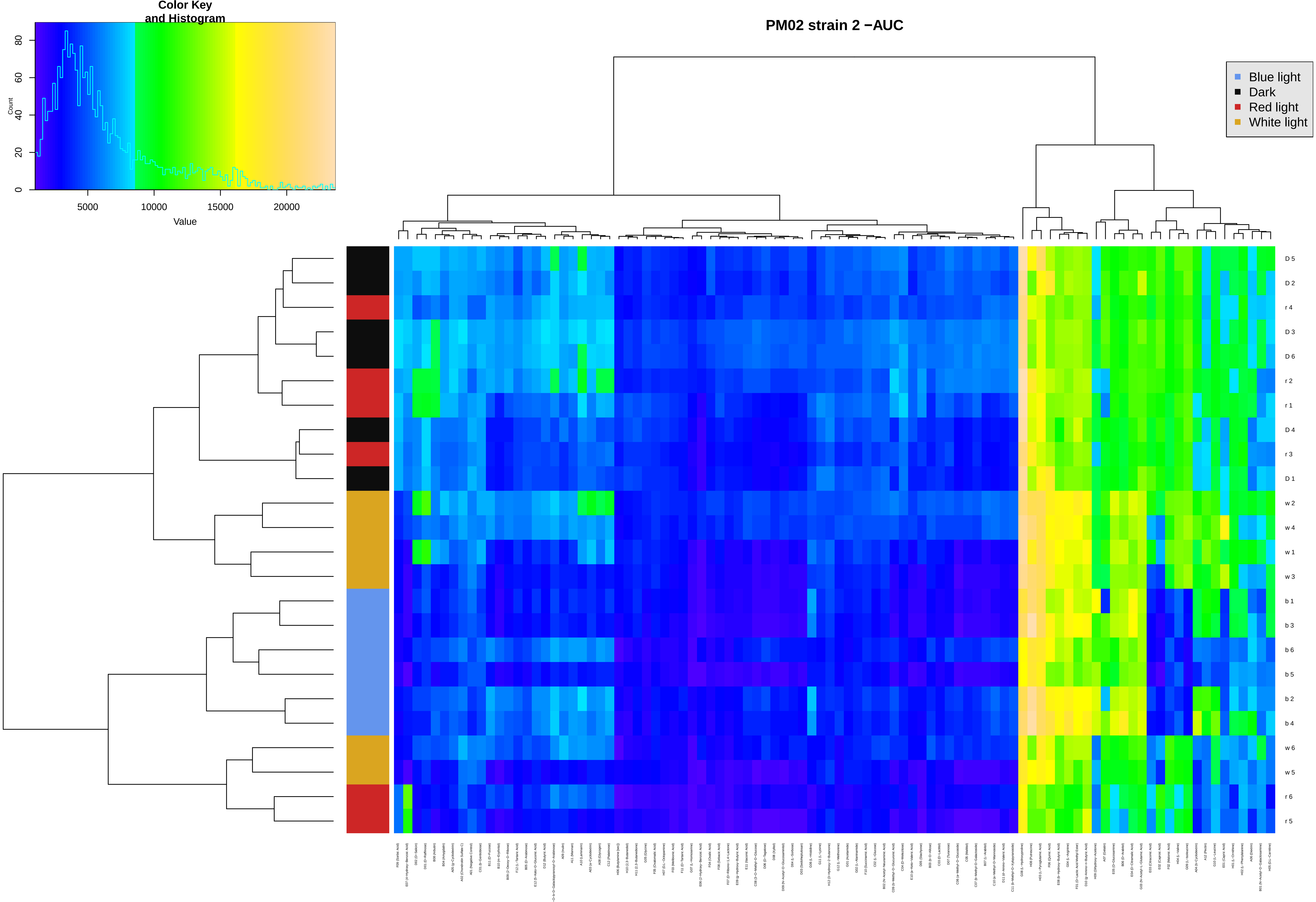
Color Key
and Histogram
Count
0
20
40
60
80
5000
10000
15000
20000
Value
PM02 strain 2 −AUC
Blue light
Dark
Red light
White light
D 5
D 2
r 4
D 3
D 6
r 2
r 1
D 4
r 3
D 1
w 2
w 4
w 1
w 3
b 1
b 3
b 6
b 5
b 2
b 4
w 6
w 5
r 6
r 5

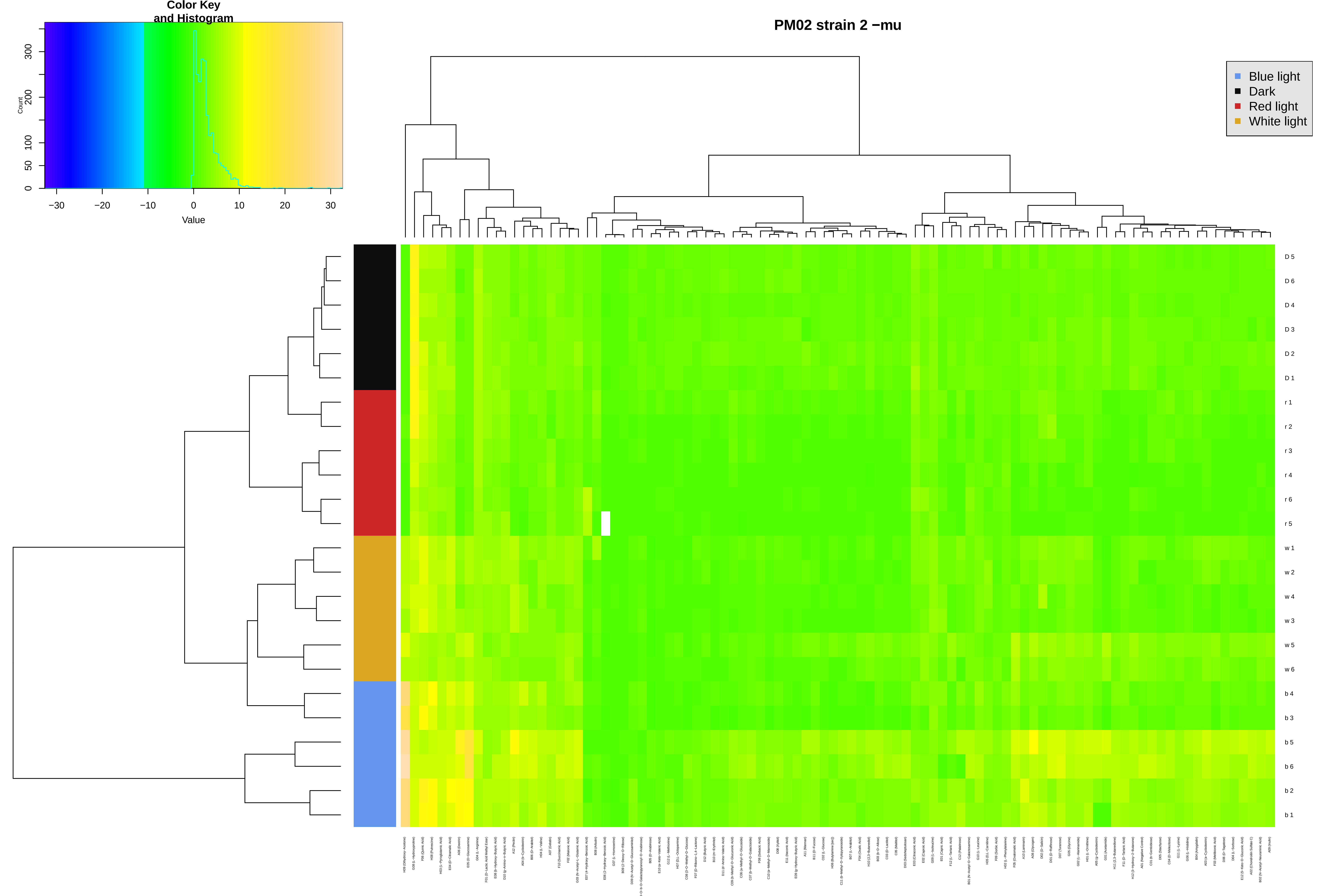
Color Key
and Histogram
Count
0
50
100
200
300
−30
−20
−10
0
10
20
30
Value
PM02 strain 2 −mu
Blue light
Dark
Red light
White light
D 5
D 6
D 4
D 3
D 2
D 1
r 1
r 2
r 3
r 4
r 6
r 5
w 1
w 2
w 4
w 3
w 5
w 6
b 4
b 3
b 5
b 6
b 2
b 1

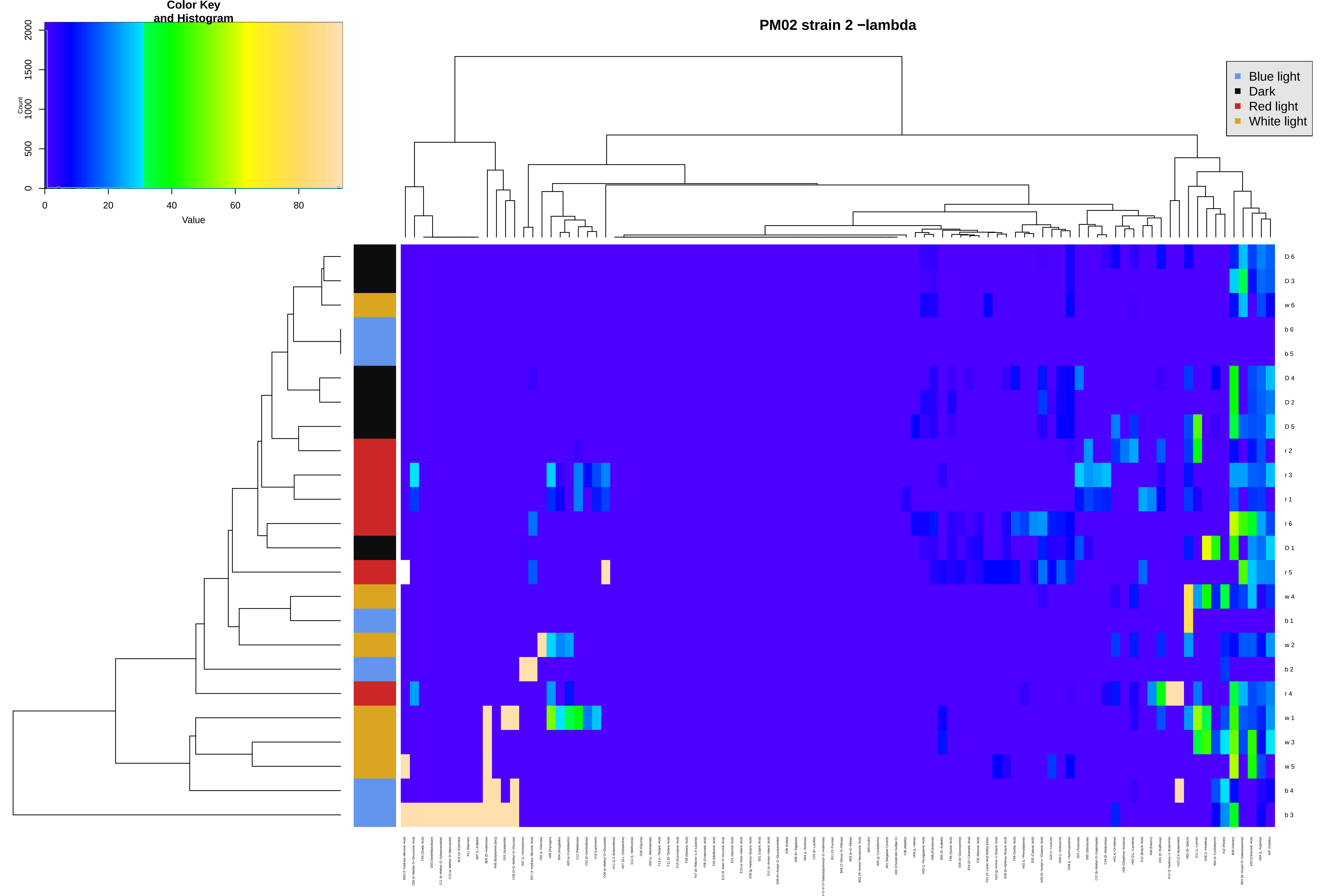

Color Key
and Histogram
Count
0
500
1000
1500
2000
0
20
40
60
80
Value
PM02 strain 2 −lambda
Blue light
Dark
Red light
White light
D 6
D 3
w 6
b 6
b 5
D 4
D 2
D 5
r 2
r 3
r 1
r 6
D 1
r 5
w 4
b 1
w 2
b 2
r 4
w 1
w 3
w 5
b 4
b 3

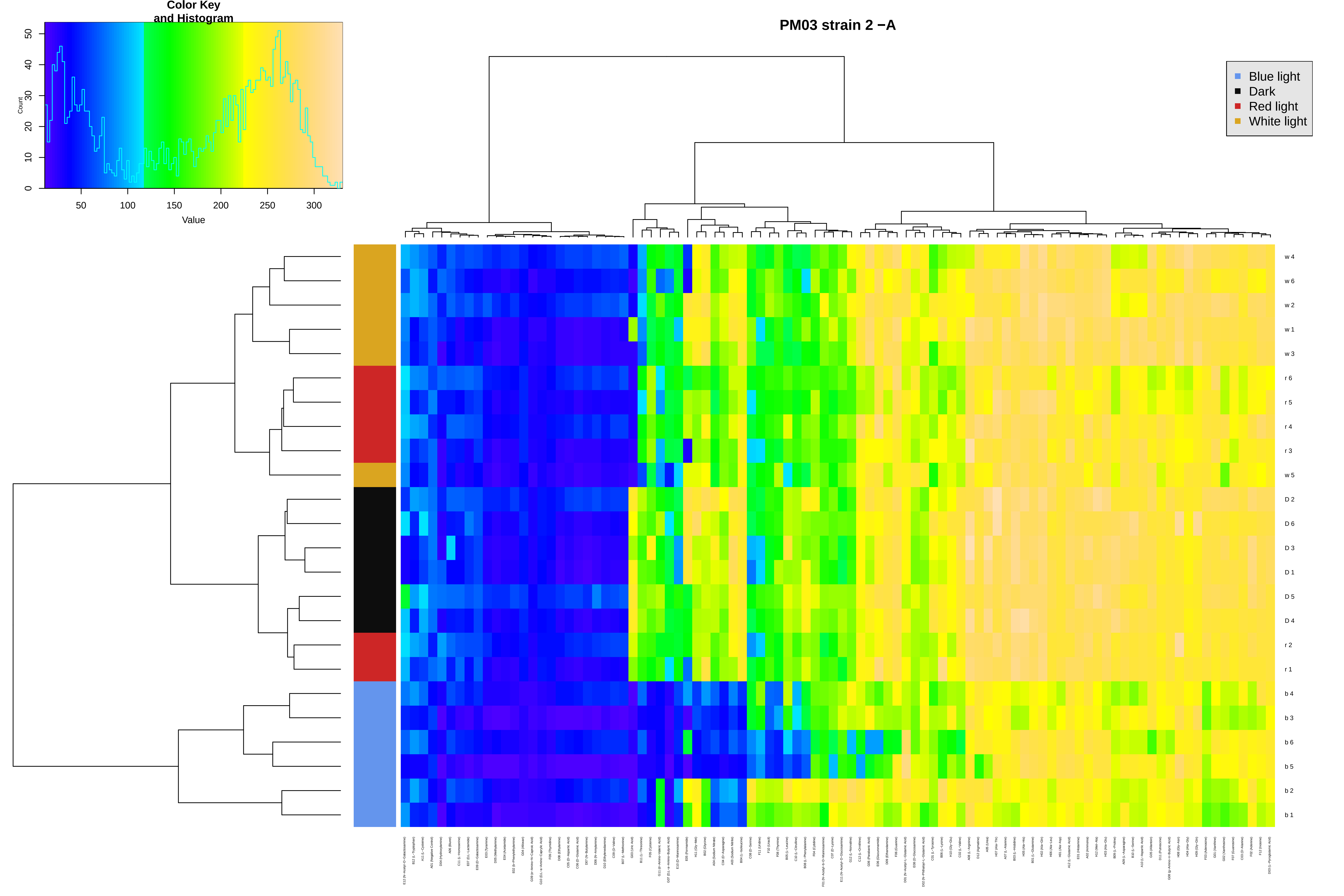
Color Key
and Histogram
Count
Value
0
10
20
30
40
50
50
100
150
200
250
300
PM03 strain 2 −A
Blue light
Dark
Red light
White light
w 4
w 6
w 2
w 1
w 3
r 6
r 5
r 4
r 3
w 5
D 2
D 6
D 3
D 1
D 5
D 4
r 2
r 1
b 4
b 3
b 6
b 5
b 2
b 1

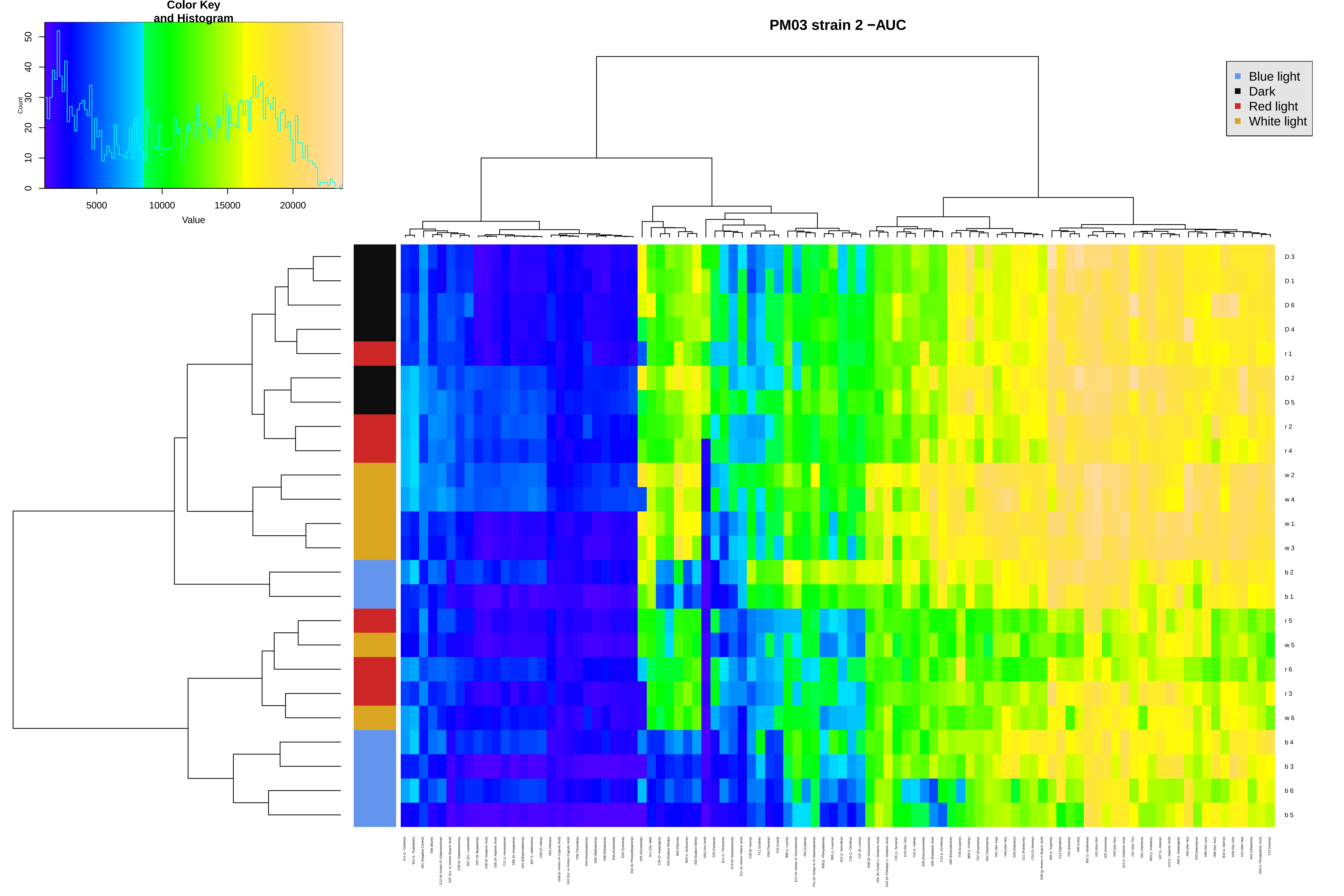
Color Key
and Histogram
Count
0
10
20
30
40
50
5000
10000
15000
20000
Value
PM03 strain 2 −AUC
Blue light
Dark
Red light
White light
D 3
D 1
D 6
D 4
r 1
D 2
D 5
r 2
r 4
w 2
w 4
w 1
w 3
b 2
b 1
r 5
w 5
r 6
r 3
w 6
b 4
b 3
b 6
b 5

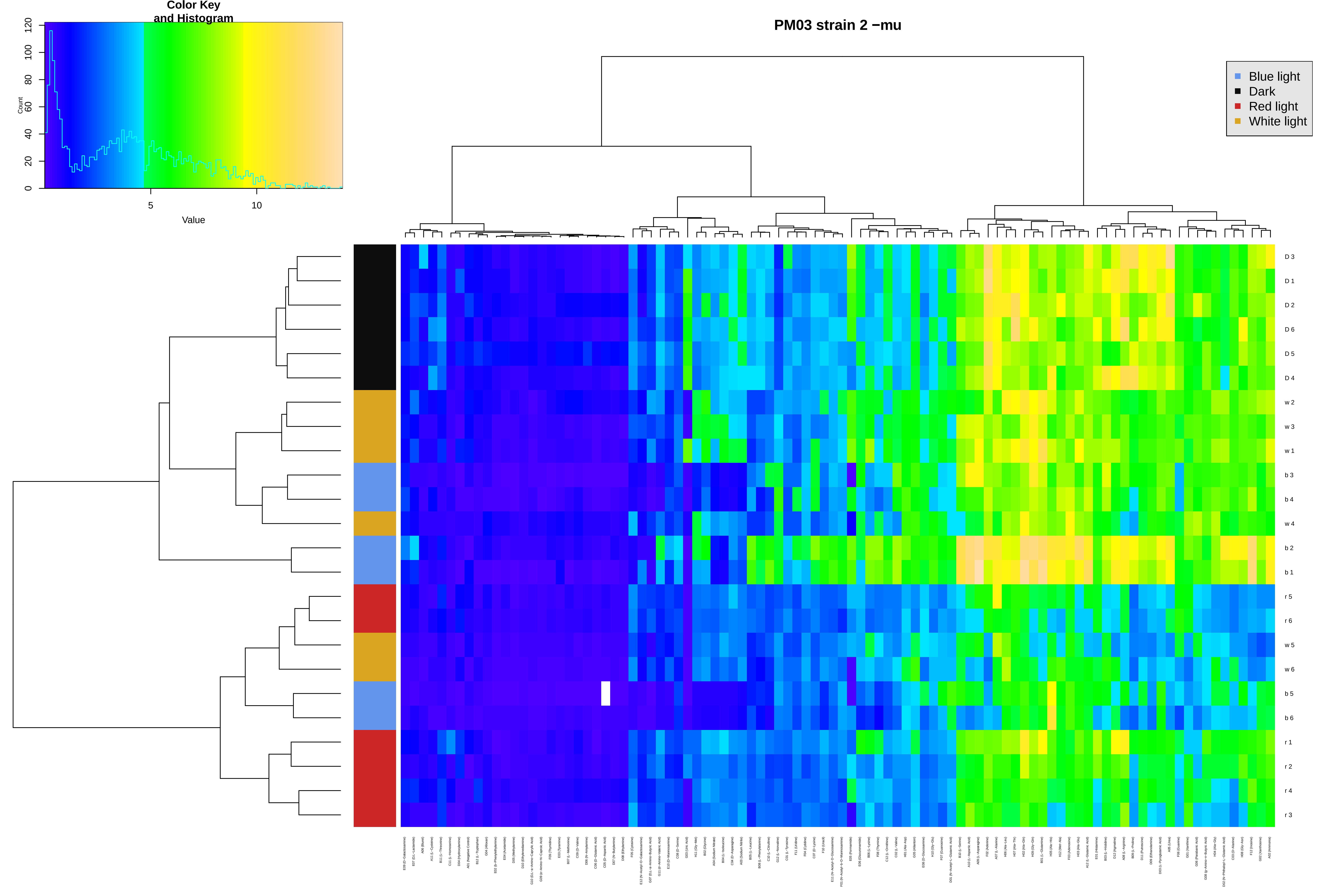
PM03 strain 2 −mu
Color Key
and Histogram
Count
Value
0
20
40
60
80
100
120
5
10
Blue light
Dark
Red light
White light
D 3
D 1
D 2
D 6
D 5
D 4
w 2
w 3
w 1
b 3
b 4
w 4
b 2
b 1
r 5
r 6
w 5
w 6
b 5
b 6
r 1
r 2
r 4
r 3

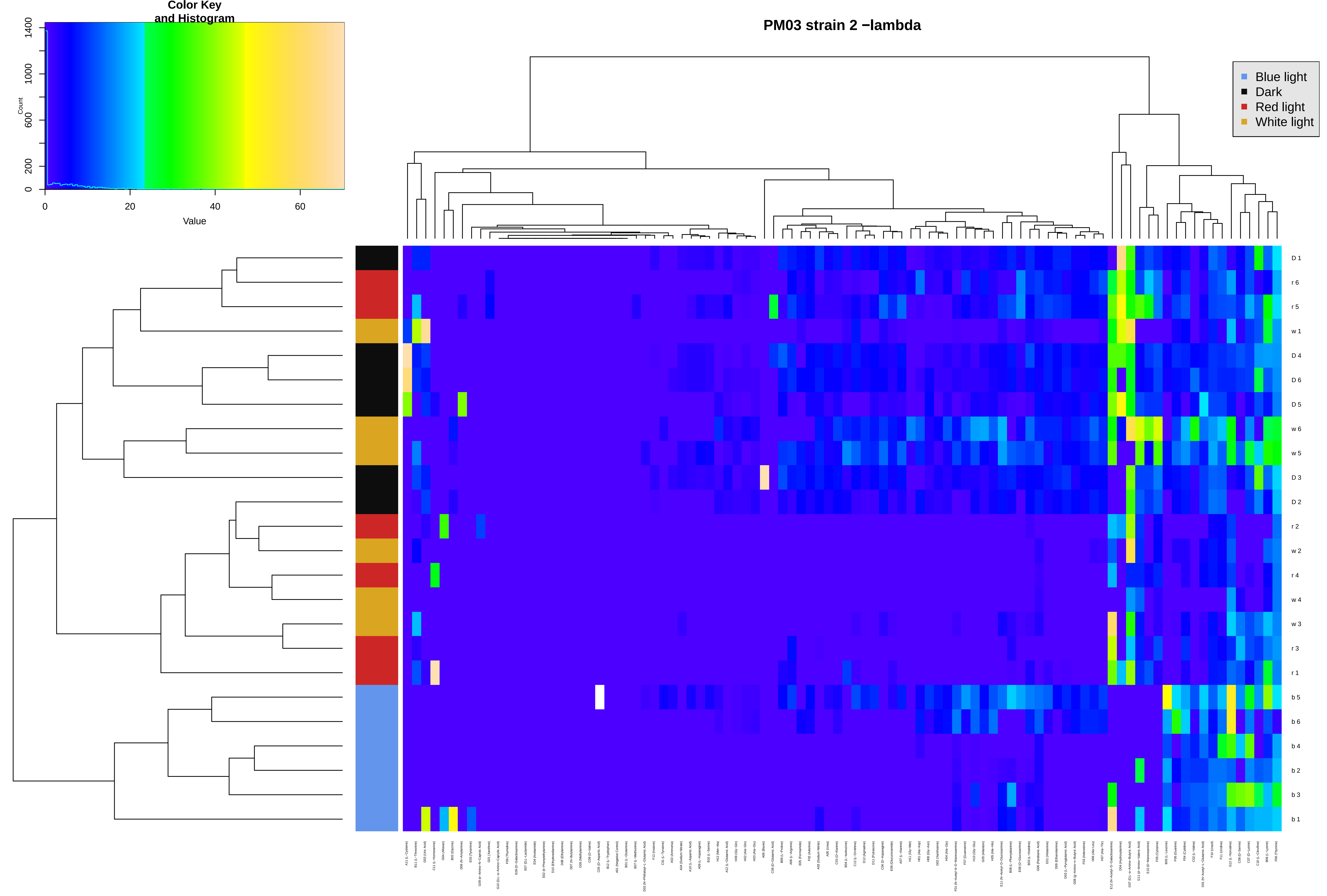
PM03 strain 2 −lambda
Color Key
and Histogram
Count
Value
0
200
600
1000
1400
0
20
40
60
Blue light
Dark
Red light
White light
D 1
r 6
r 5
w 1
D 4
D 6
D 5
w 6
w 5
D 3
D 2
r 2
w 2
r 4
w 4
w 3
r 3
r 1
b 5
b 6
b 4
b 2
b 3
b 1

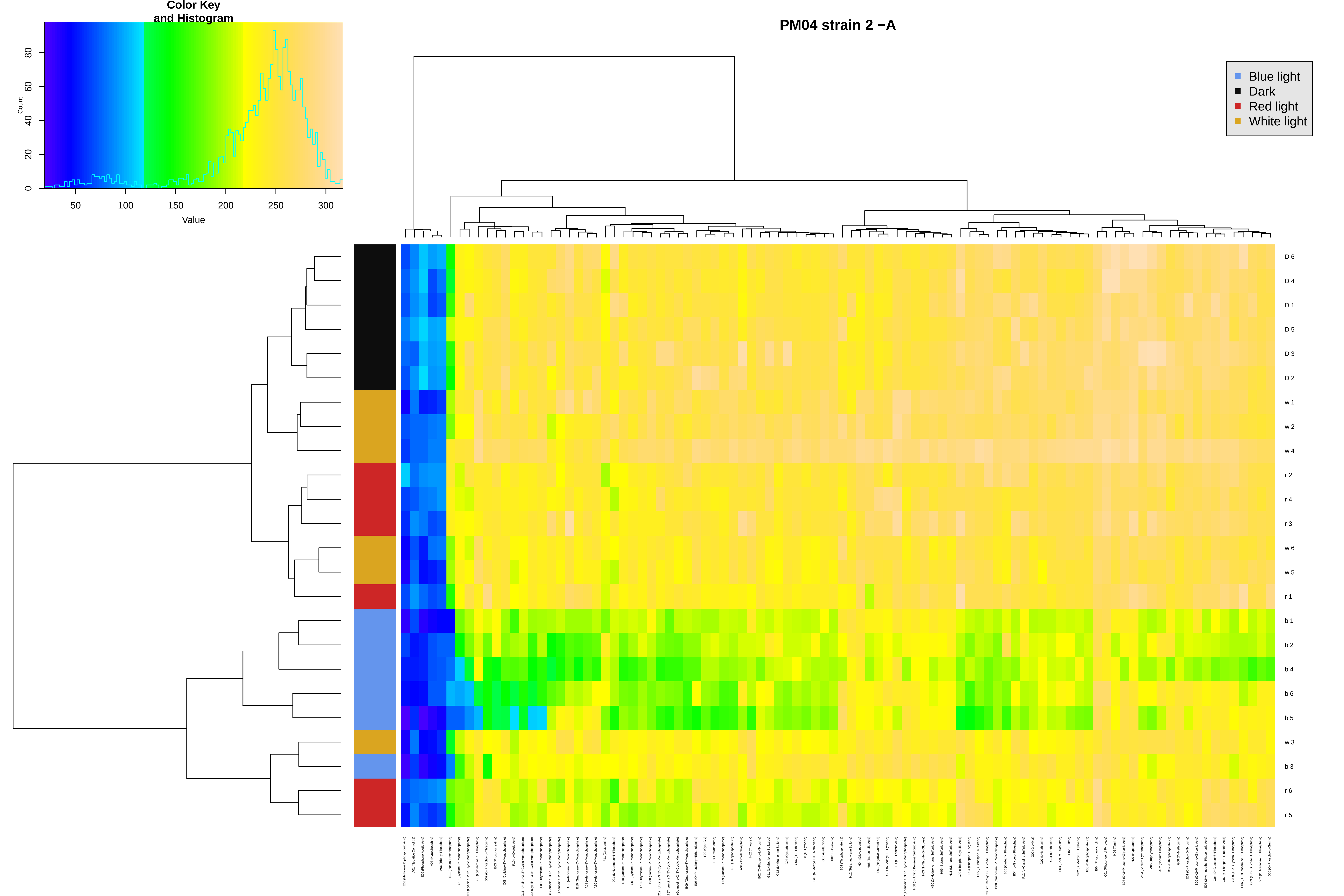
Color Key
and Histogram
Count
0
20
40
60
80
50
100
150
200
250
300
Value
PM04 strain 2 −A
Blue light
Dark
Red light
White light
D 6
D 4
D 1
D 5
D 3
D 2
w 1
w 2
w 4
r 2
r 4
r 3
w 6
w 5
r 1
b 1
b 2
b 4
b 6
b 5
w 3
b 3
r 6
r 5

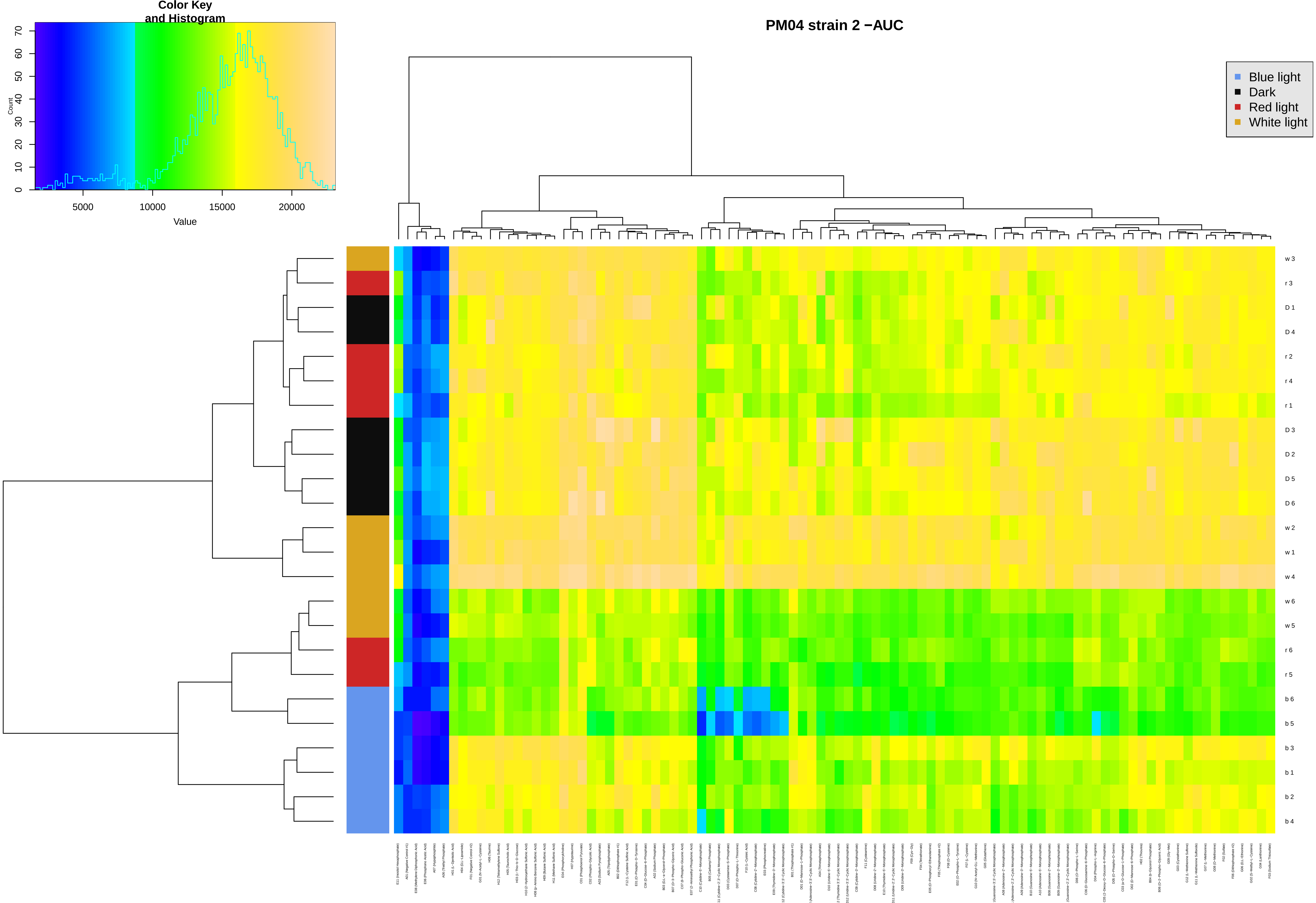

Color Key and Histogram
Count
0
10
20
30
40
50
60
70
5000
10000
15000
20000
Value
PM04 strain 2 −AUC
Blue light
Dark
Red light
White light
w 3
r 3
D 1
D 4
r 2
r 4
r 1
D 3
D 2
D 5
D 6
w 2
w 1
w 4
w 6
w 5
r 6
r 5
b 6
b 5
b 3
b 1
b 2
b 4

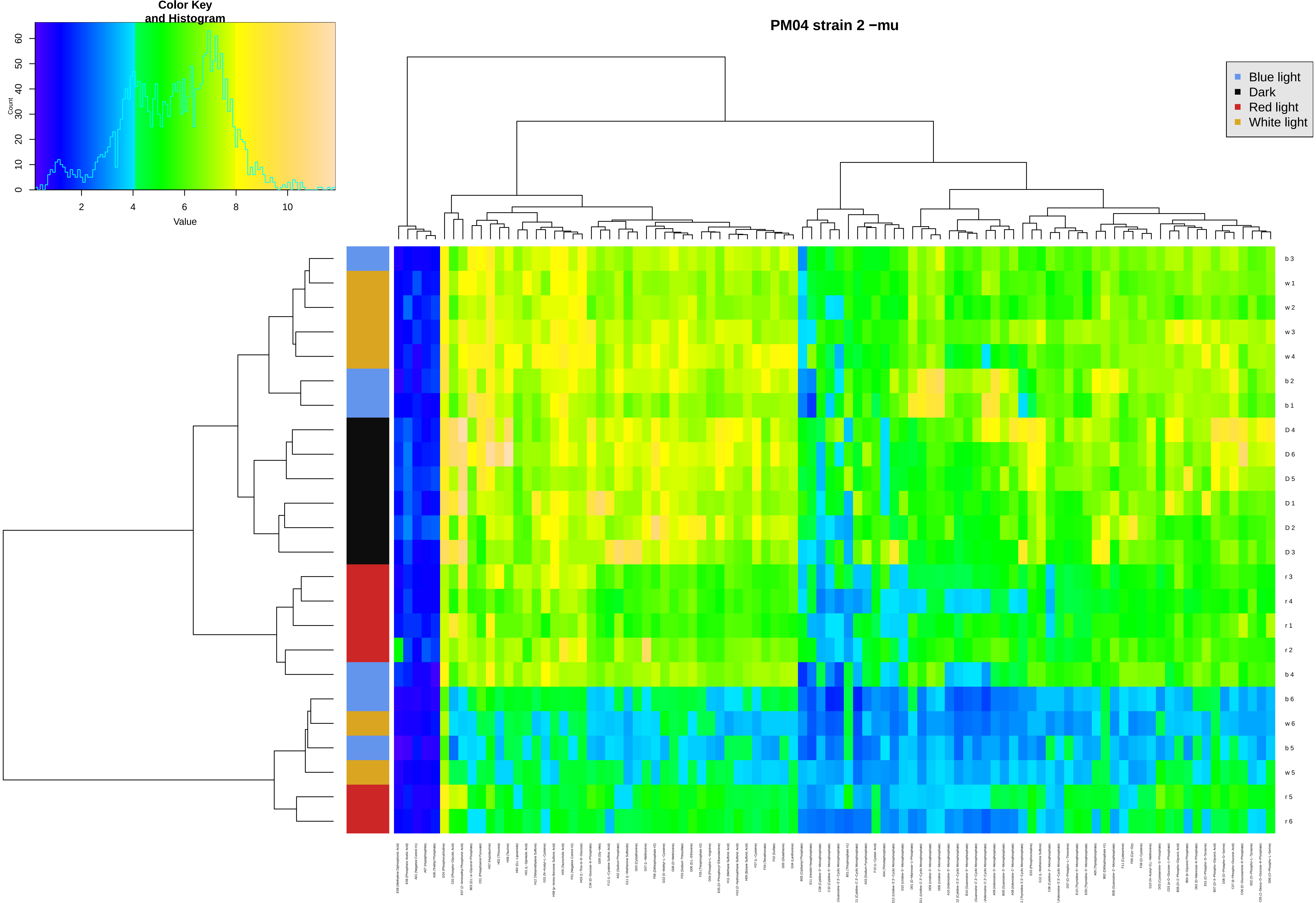
PM04 strain 2 −mu
Color Key
and Histogram
Count
Value
0
10
20
30
40
50
60
2
4
6
8
10
Blue light
Dark
Red light
White light
b 3
w 1
w 2
w 3
w 4
b 2
b 1
D 4
D 6
D 5
D 1
D 2
D 3
r 3
r 4
r 1
r 2
b 4
b 6
w 6
b 5
w 5
r 5
r 6

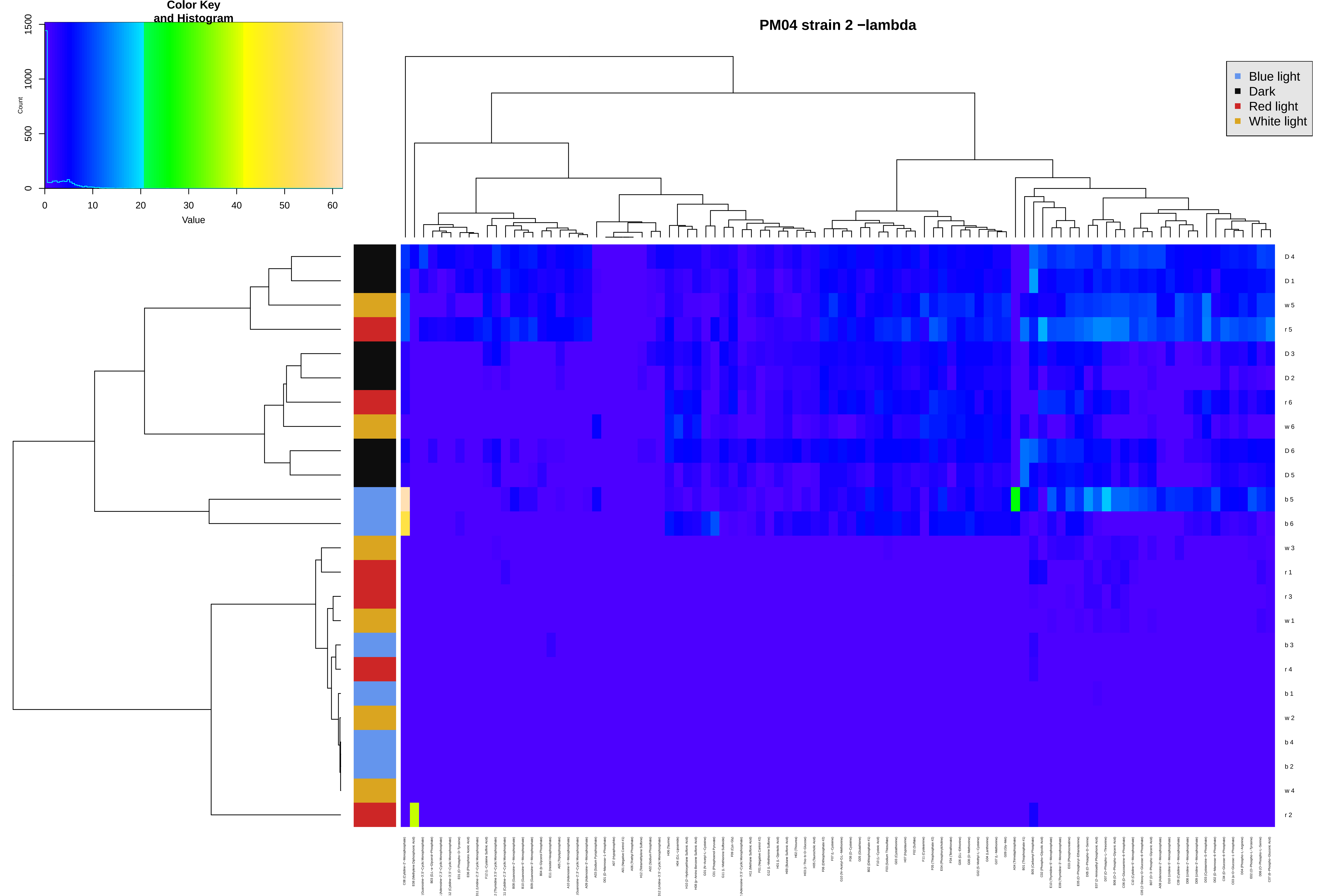

Color Key
and Histogram
Count
Value
PM04 strain 2 −lambda
Blue light
Dark
Red light
White light
D 4
D 1
w 5
r 5
D 3
D 2
r 6
w 6
D 6
D 5
b 5
b 6
w 3
r 1
r 3
w 1
b 3
r 4
b 1
w 2
b 4
b 2
w 4
r 2
