# Supplementary material for: Light spectrum modifies the utilization pattern of energy sources in Pseudomonas sp. DR 5-09
Source: PLoS One. 2017 Dec 21;12(12):e0189862. doi: 10.1371/journal.pone.0189862 (PMC5739431; doi:10.1371/journal.pone.0189862)

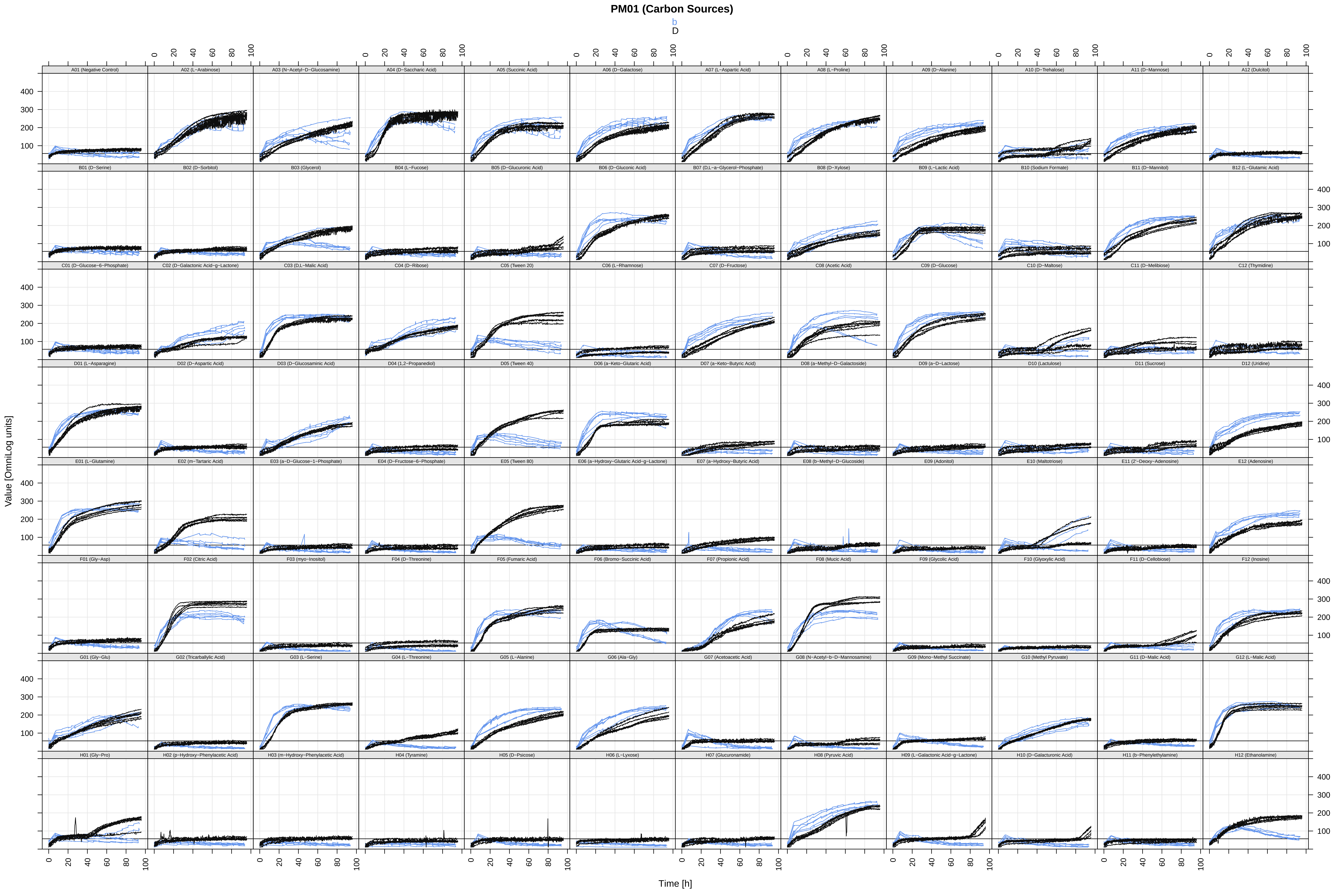

PM01 (Carbon Sources)
b
D
A01 (Negative Control)
A02 (L−Arabinose)
A03 (N−Acetyl−D−Glucosamine)
A04 (D−Saccharic Acid)
A05 (Succinic Acid)
A06 (D−Galactose)
A07 (L−Aspartic Acid)
A08 (L−Proline)
A09 (D−Alanine)
A10 (D−Trehalose)
A11 (D−Mannose)
A12 (Dulcitol)
B01 (D−Serine)
B02 (D−Sorbitol)
B03 (Glycerol)
B04 (L−Fucose)
B05 (D−Glucuronic Acid)
B06 (D−Gluconic Acid)
B07 (D,L−a−Glycerol−Phosphate)
B08 (D−Xylose)
B09 (L−Lactic Acid)
B10 (Sodium Formate)
B11 (D−Mannitol)
B12 (L−Glutamic Acid)
C01 (D−Glucose−6−Phosphate)
C02 (D−Galactonic Acid−g−Lactone)
C03 (D,L−Malic Acid)
C04 (D−Ribose)
C05 (Tween 20)
C06 (L−Rhamnose)
C07 (D−Fructose)
C08 (Acetic Acid)
C09 (D−Glucose)
C10 (D−Maltose)
C11 (D−Melibiose)
C12 (Thymidine)
D01 (L−Asparagine)
D02 (D−Aspartic Acid)
D03 (D−Glucosaminic Acid)
D04 (1,2−Propanediol)
D05 (Tween 40)
D06 (a−Keto−Glutaric Acid)
D07 (a−Keto−Butyric Acid)
D08 (a−Methyl−D−Galactoside)
D09 (a−D−Lactose)
D10 (Lactulose)
D11 (Sucrose)
D12 (Uridine)
E01 (L−Glutamine)
E02 (m−Tartaric Acid)
E03 (a−D−Glucose−1−Phosphate)
E04 (D−Fructose−6−Phosphate)
E05 (Tween 80)
E06 (a−Hydroxy−Glutaric Acid−g−Lactone)
E07 (a−Hydroxy−Butyric Acid)
E08 (b−Methyl−D−Glucoside)
E09 (Adonitol)
E10 (Maltotriose)
E11 (2'−Deoxy−Adenosine)
E12 (Adenosine)
F01 (Gly−Asp)
F02 (Citric Acid)
F03 (myo−Inositol)
F04 (D−Threonine)
F05 (Fumaric Acid)
F06 (Bromo−Succinic Acid)
F07 (Propionic Acid)
F08 (Mucic Acid)
F09 (Glycolic Acid)
F10 (Glyoxylic Acid)
F11 (D−Cellobiose)
F12 (Inosine)
G01 (Gly−Glu)
G02 (Tricarballylic Acid)
G03 (L−Serine)
G04 (L−Threonine)
G05 (L−Alanine)
G06 (Ala−Gly)
G07 (Acetoacetic Acid)
G08 (N−Acetyl−b−D−Mannosamine)
G09 (Mono−Methyl Succinate)
G10 (Methyl Pyruvate)
G11 (D−Malic Acid)
G12 (L−Malic Acid)
H01 (Gly−Pro)
H02 (p−Hydroxy−Phenylacetic Acid)
H03 (m−Hydroxy−Phenylacetic Acid)
H04 (Tyramine)
H05 (D−Psicose)
H06 (L−Lyxose)
H07 (Glucuronamide)
H08 (Pyruvic Acid)
H09 (L−Galactonic Acid−g−Lactone)
H10 (D−Galacturonic Acid)
H11 (b−Phenylethylamine)
H12 (Ethanolamine)
Value [OmniLog units]
Time [h]
0
20
40
60
80
100
200
300
400

PM02 (Carbon Sources)

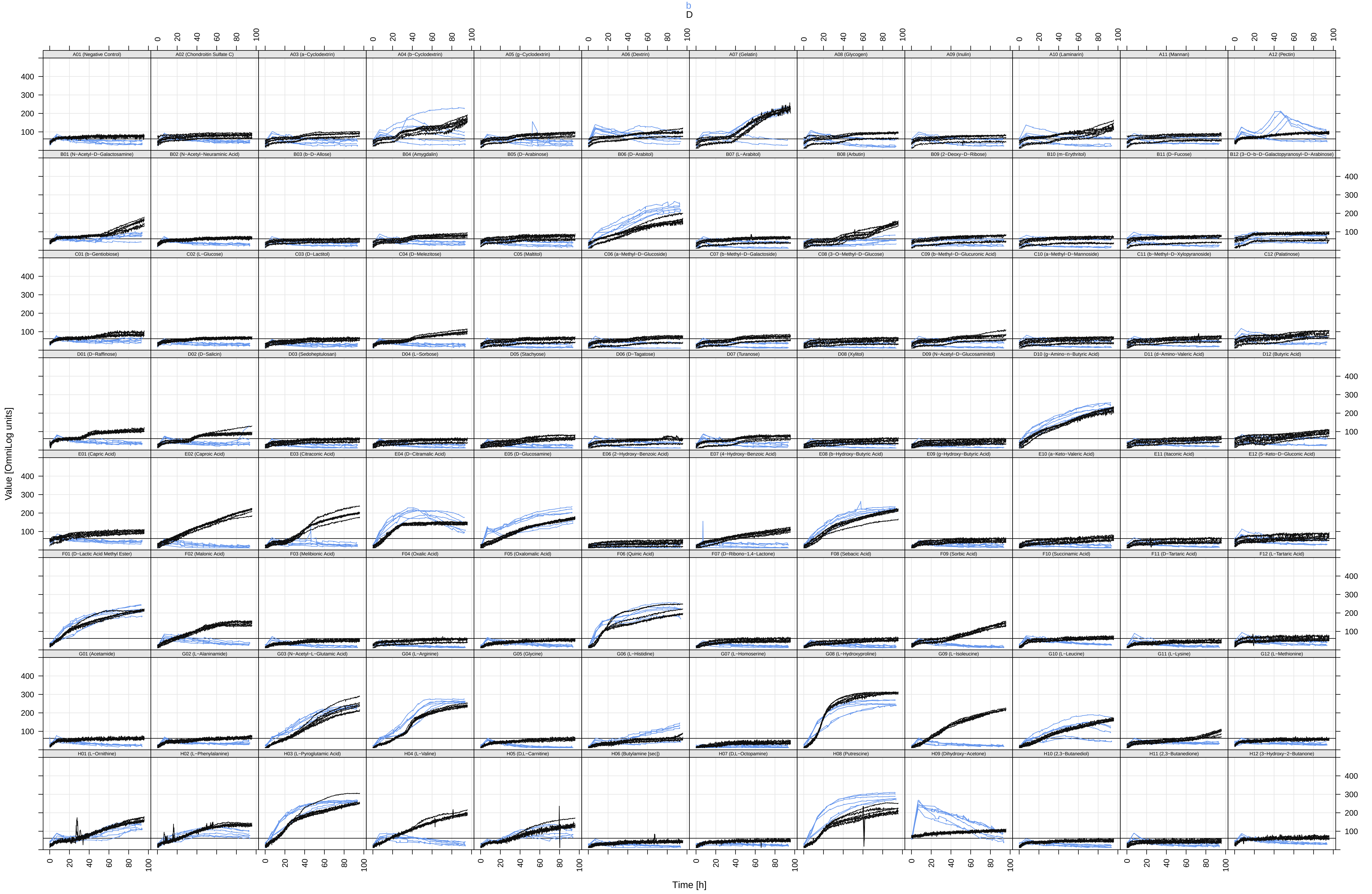

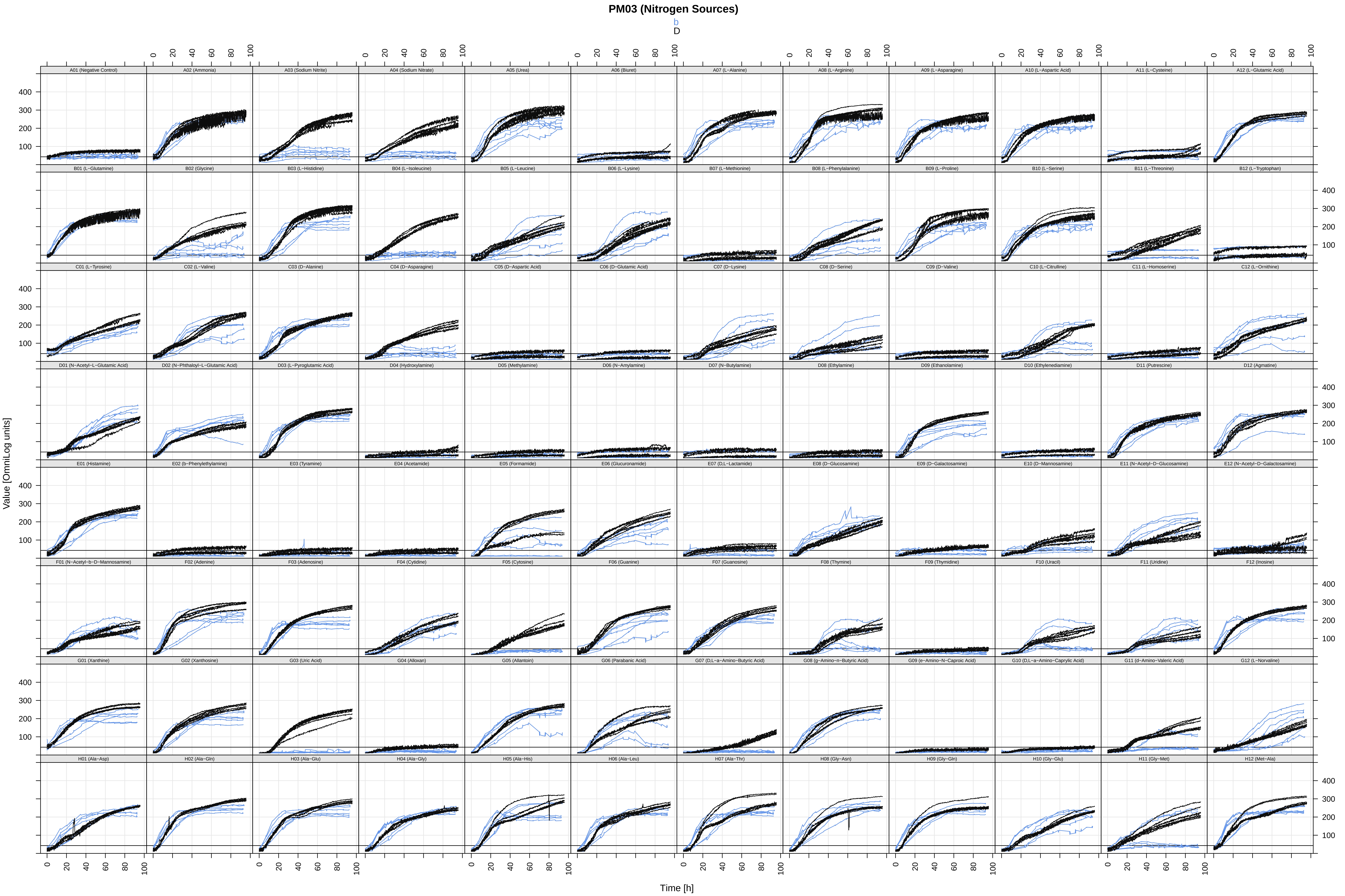
PM03 (Nitrogen Sources)
b
D
Value [OmniLog units]
Time [h]
A01 (Negative Control)
A02 (Ammonia)
A03 (Sodium Nitrite)
A04 (Sodium Nitrate)
A05 (Urea)
A06 (Biuret)
A07 (L−Alanine)
A08 (L−Arginine)
A09 (L−Asparagine)
A10 (L−Aspartic Acid)
A11 (L−Cysteine)
A12 (L−Glutamic Acid)
B01 (L−Glutamine)
B02 (Glycine)
B03 (L−Histidine)
B04 (L−Isoleucine)
B05 (L−Leucine)
B06 (L−Lysine)
B07 (L−Methionine)
B08 (L−Phenylalanine)
B09 (L−Proline)
B10 (L−Serine)
B11 (L−Threonine)
B12 (L−Tryptophan)
C01 (L−Tyrosine)
C02 (L−Valine)
C03 (D−Alanine)
C04 (D−Asparagine)
C05 (D−Aspartic Acid)
C06 (D−Glutamic Acid)
C07 (D−Lysine)
C08 (D−Serine)
C09 (D−Valine)
C10 (L−Citrulline)
C11 (L−Homoserine)
C12 (L−Ornithine)
D01 (N−Acetyl−L−Glutamic Acid)
D02 (N−Phthaloyl−L−Glutamic Acid)
D03 (L−Pyroglutamic Acid)
D04 (Hydroxylamine)
D05 (Methylamine)
D06 (N−Amylamine)
D07 (N−Butylamine)
D08 (Ethylamine)
D09 (Ethanolamine)
D10 (Ethylenediamine)
D11 (Putrescine)
D12 (Agmatine)
E01 (Histamine)
E02 (b−Phenylethylamine)
E03 (Tyramine)
E04 (Acetamide)
E05 (Formamide)
E06 (Glucuronamide)
E07 (D,L−Lactamide)
E08 (D−Glucosamine)
E09 (D−Galactosamine)
E10 (D−Mannosamine)
E11 (N−Acetyl−D−Glucosamine)
E12 (N−Acetyl−D−Galactosamine)
F01 (N−Acetyl−b−D−Mannosamine)
F02 (Adenine)
F03 (Adenosine)
F04 (Cytidine)
F05 (Cytosine)
F06 (Guanine)
F07 (Guanosine)
F08 (Thymine)
F09 (Thymidine)
F10 (Uracil)
F11 (Uridine)
F12 (Inosine)
G01 (Xanthine)
G02 (Xanthosine)
G03 (Uric Acid)
G04 (Alloxan)
G05 (Allantoin)
G06 (Parabanic Acid)
G07 (D,L−a−Amino−Butyric Acid)
G08 (g−Amino−n−Butyric Acid)
G09 (e−Amino−N−Caproic Acid)
G10 (D,L−a−Amino−Caprylic Acid)
G11 (d−Amino−Valeric Acid)
G12 (L−Norvaline)
H01 (Ala−Asp)
H02 (Ala−Gln)
H03 (Ala−Glu)
H04 (Ala−Gly)
H05 (Ala−His)
H06 (Ala−Leu)
H07 (Ala−Thr)
H08 (Gly−Asn)
H09 (Gly−Gln)
H10 (Gly−Glu)
H11 (Gly−Met)
H12 (Met−Ala)
0 20 40 60 80 100
100 200 300 400

PM04 (Phosphorus and Sulfur Sources)

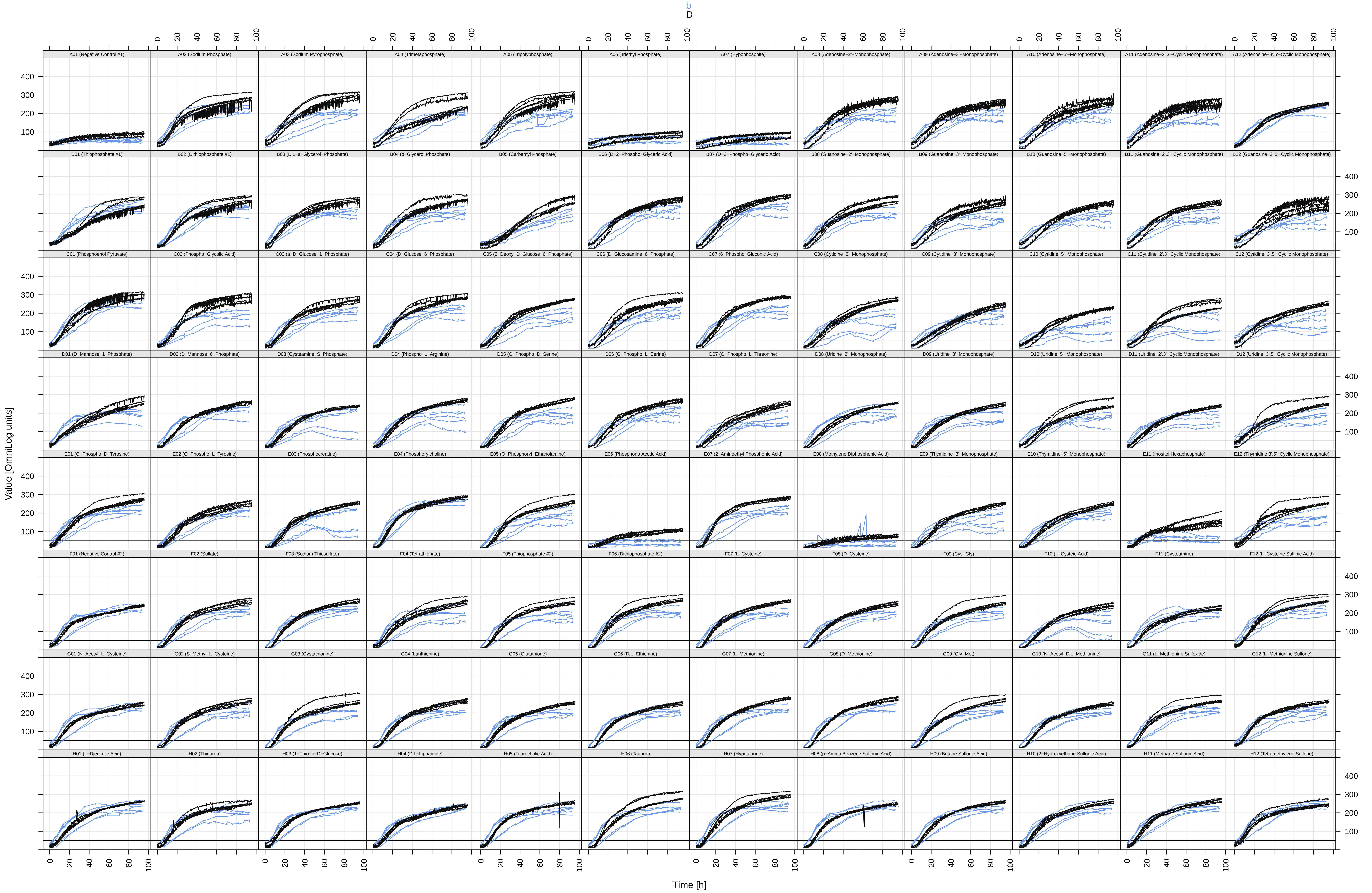

PM01 (Carbon Sources)

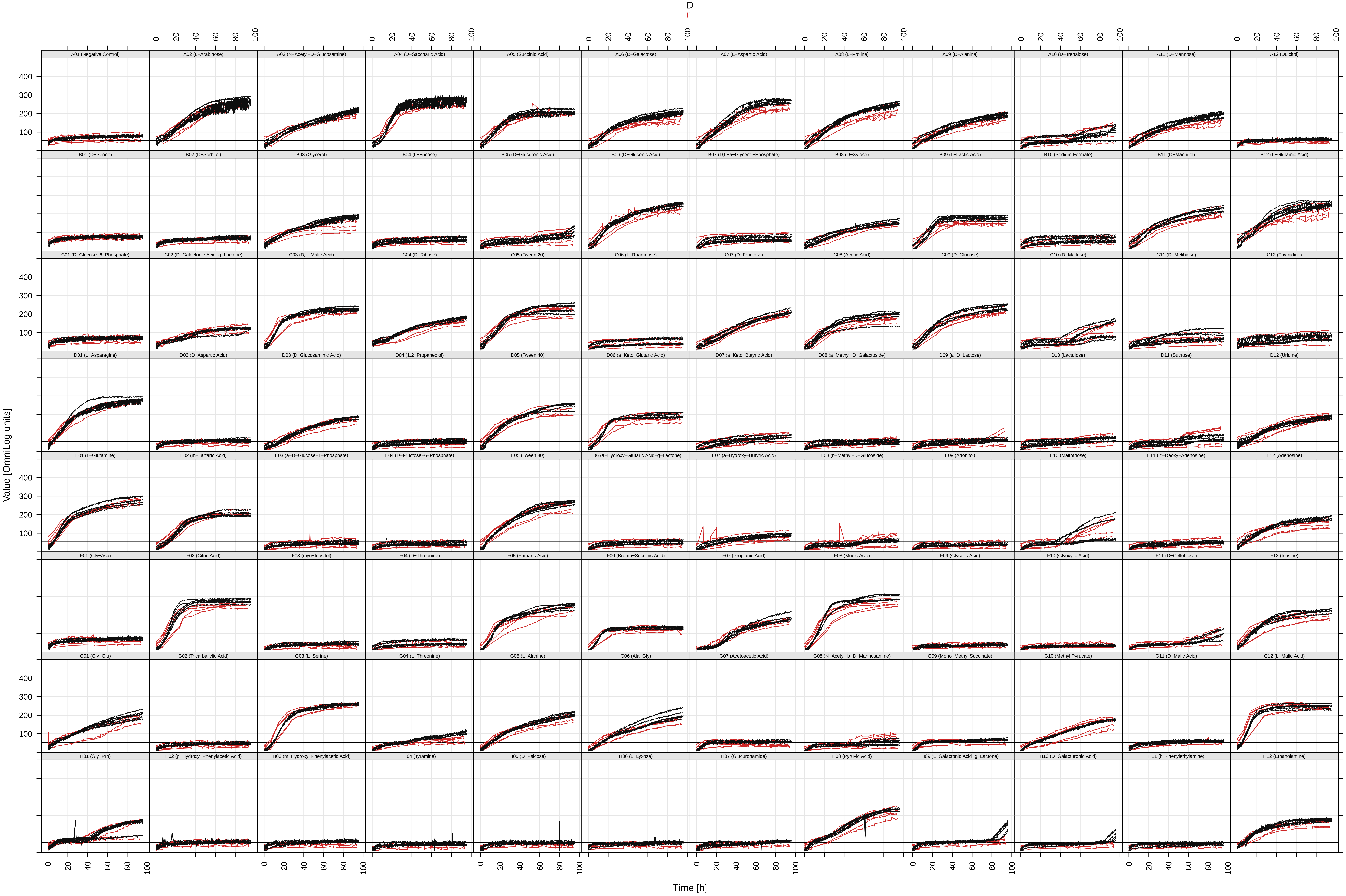

PM02 (Carbon Sources)

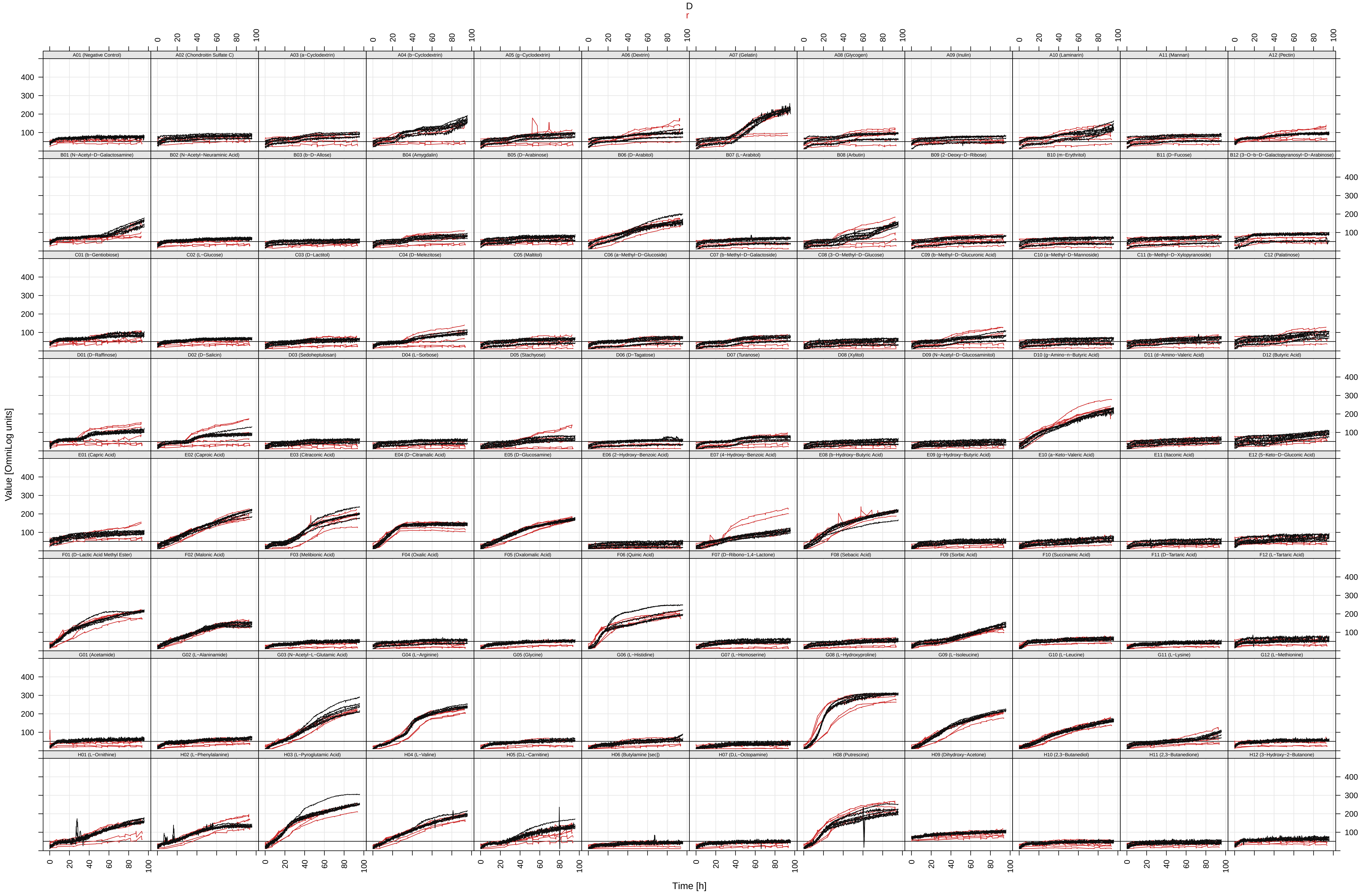

PM03 (Nitrogen Sources)

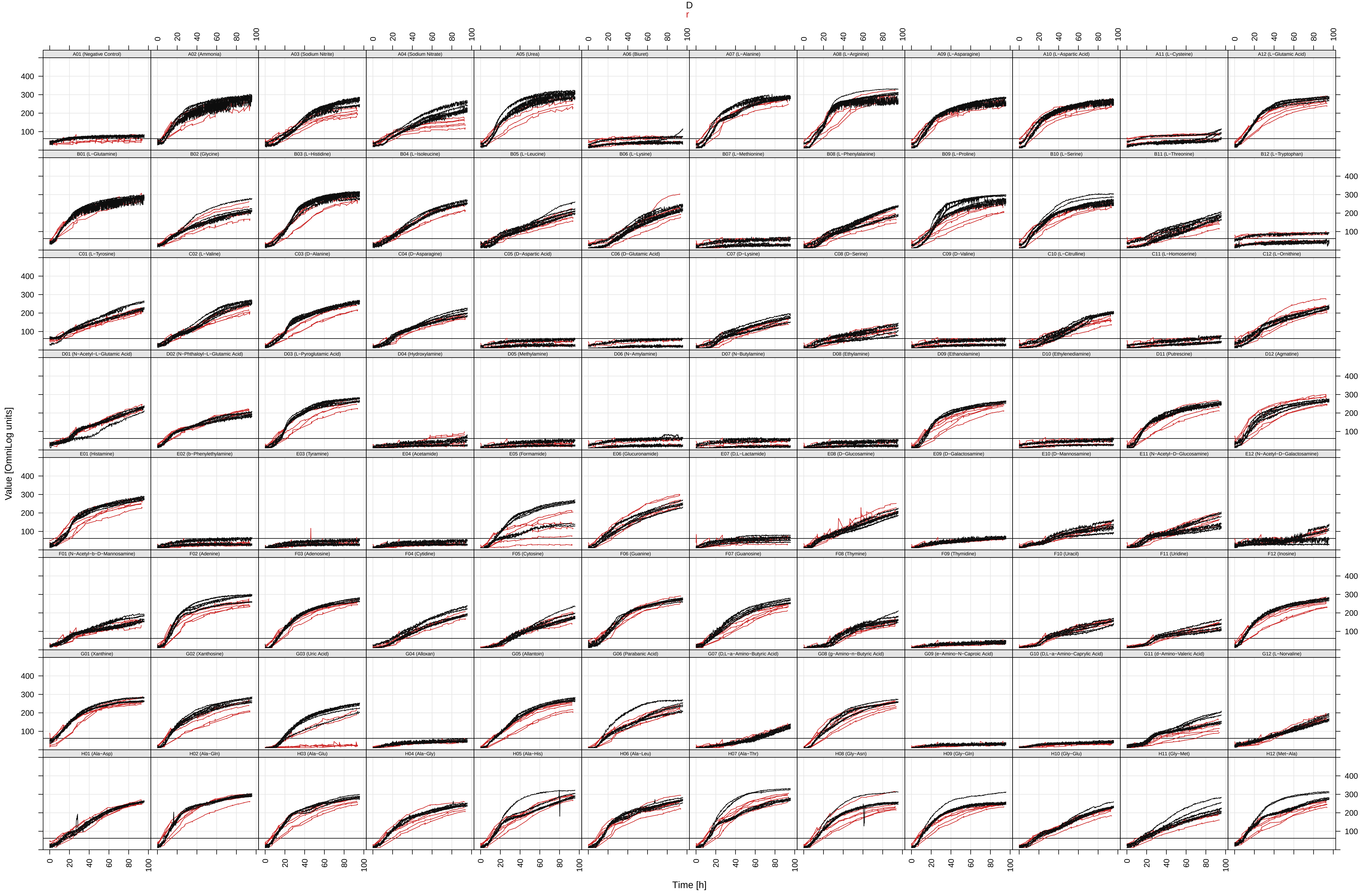

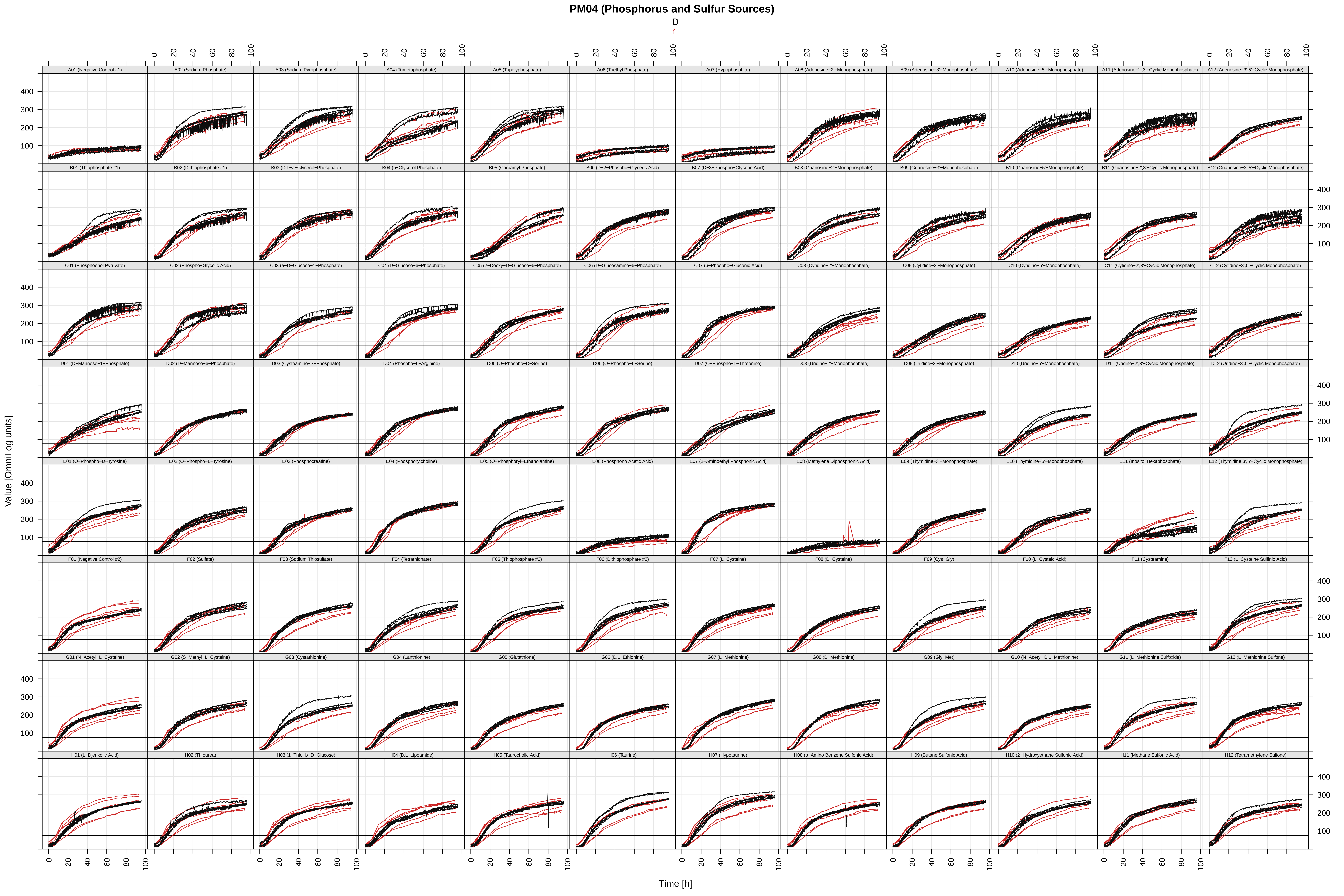

PM04 (Phosphorus and Sulfur Sources)
D
r
Value [OmniLog units]
Time [h]
A01 (Negative Control #1)
A02 (Sodium Phosphate)
A03 (Sodium Pyrophosphate)
A04 (Trimetaphosphate)
A05 (Tripolyphosphate)
A06 (Triethyl Phosphate)
A07 (Hypophosphite)
A08 (Adenosine−2'−Monophosphate)
A09 (Adenosine−3'−Monophosphate)
A10 (Adenosine−5'−Monophosphate)
A11 (Adenosine−2',3'−Cyclic Monophosphate)
A12 (Adenosine−3',5'−Cyclic Monophosphate)
B01 (Thiophosphate #1)
B02 (Dithiophosphate #1)
B03 (D,L−a−Glycerol−Phosphate)
B04 (b−Glycerol Phosphate)
B05 (Carbamyl Phosphate)
B06 (D−2−Phospho−Glyceric Acid)
B07 (D−3−Phospho−Glyceric Acid)
B08 (Guanosine−2'−Monophosphate)
B09 (Guanosine−3'−Monophosphate)
B10 (Guanosine−5'−Monophosphate)
B11 (Guanosine−2',3'−Cyclic Monophosphate)
B12 (Guanosine−3',5'−Cyclic Monophosphate)
C01 (Phosphoenol Pyruvate)
C02 (Phospho−Glycolic Acid)
C03 (a−D−Glucose−1−Phosphate)
C04 (D−Glucose−6−Phosphate)
C05 (2−Deoxy−D−Glucose−6−Phosphate)
C06 (D−Glucosamine−6−Phosphate)
C07 (6−Phospho−Gluconic Acid)
C08 (Cytidine−2'−Monophosphate)
C09 (Cytidine−3'−Monophosphate)
C10 (Cytidine−5'−Monophosphate)
C11 (Cytidine−2',3'−Cyclic Monophosphate)
C12 (Cytidine−3',5'−Cyclic Monophosphate)
D01 (D−Mannose−1−Phosphate)
D02 (D−Mannose−6−Phosphate)
D03 (Cysteamine−S−Phosphate)
D04 (Phospho−L−Arginine)
D05 (O−Phospho−D−Serine)
D06 (O−Phospho−L−Serine)
D07 (O−Phospho−L−Threonine)
D08 (Uridine−2'−Monophosphate)
D09 (Uridine−3'−Monophosphate)
D10 (Uridine−5'−Monophosphate)
D11 (Uridine−2',3'−Cyclic Monophosphate)
D12 (Uridine−3',5'−Cyclic Monophosphate)
E01 (O−Phospho−D−Tyrosine)
E02 (O−Phospho−L−Tyrosine)
E03 (Phosphocreatine)
E04 (Phosphorylcholine)
E05 (O−Phosphoryl−Ethanolamine)
E06 (Phosphono Acetic Acid)
E07 (2−Aminoethyl Phosphonic Acid)
E08 (Methylene Diphosphonic Acid)
E09 (Thymidine−3'−Monophosphate)
E10 (Thymidine−5'−Monophosphate)
E11 (Inositol Hexaphosphate)
E12 (Thymidine 3',5'−Cyclic Monophosphate)
F01 (Negative Control #2)
F02 (Sulfate)
F03 (Sodium Thiosulfate)
F04 (Tetrathionate)
F05 (Thiophosphate #2)
F06 (Dithiophosphate #2)
F07 (L−Cysteine)
F08 (D−Cysteine)
F09 (Cys−Gly)
F10 (L−Cysteic Acid)
F11 (Cysteamine)
F12 (L−Cysteine Sulfinic Acid)
G01 (N−Acetyl−L−Cysteine)
G02 (S−Methyl−L−Cysteine)
G03 (Cystathionine)
G04 (Lanthionine)
G05 (Glutathione)
G06 (D,L−Ethionine)
G07 (L−Methionine)
G08 (D−Methionine)
G09 (Gly−Met)
G10 (N−Acetyl−D,L−Methionine)
G11 (L−Methionine Sulfoxide)
G12 (L−Methionine Sulfone)
H01 (L−Djenkolic Acid)
H02 (Thiourea)
H03 (1−Thio−b−D−Glucose)
H04 (D,L−Lipoamide)
H05 (Taurocholic Acid)
H06 (Taurine)
H07 (Hypotaurine)
H08 (p−Amino Benzene Sulfonic Acid)
H09 (Butane Sulfonic Acid)
H10 (2−Hydroxyethane Sulfonic Acid)
H11 (Methane Sulfonic Acid)
H12 (Tetramethylene Sulfone)

PM01 (Carbon Sources)

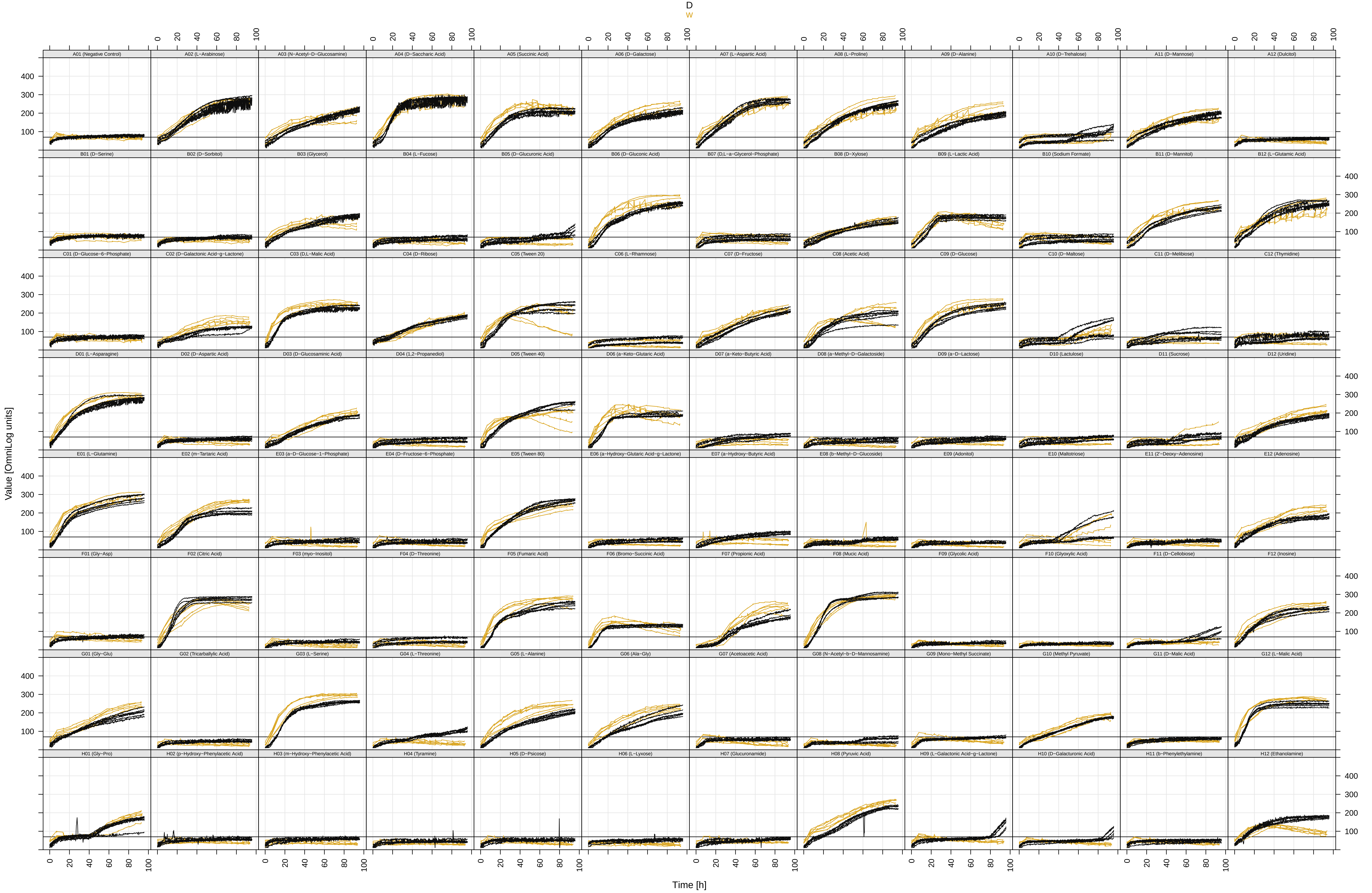

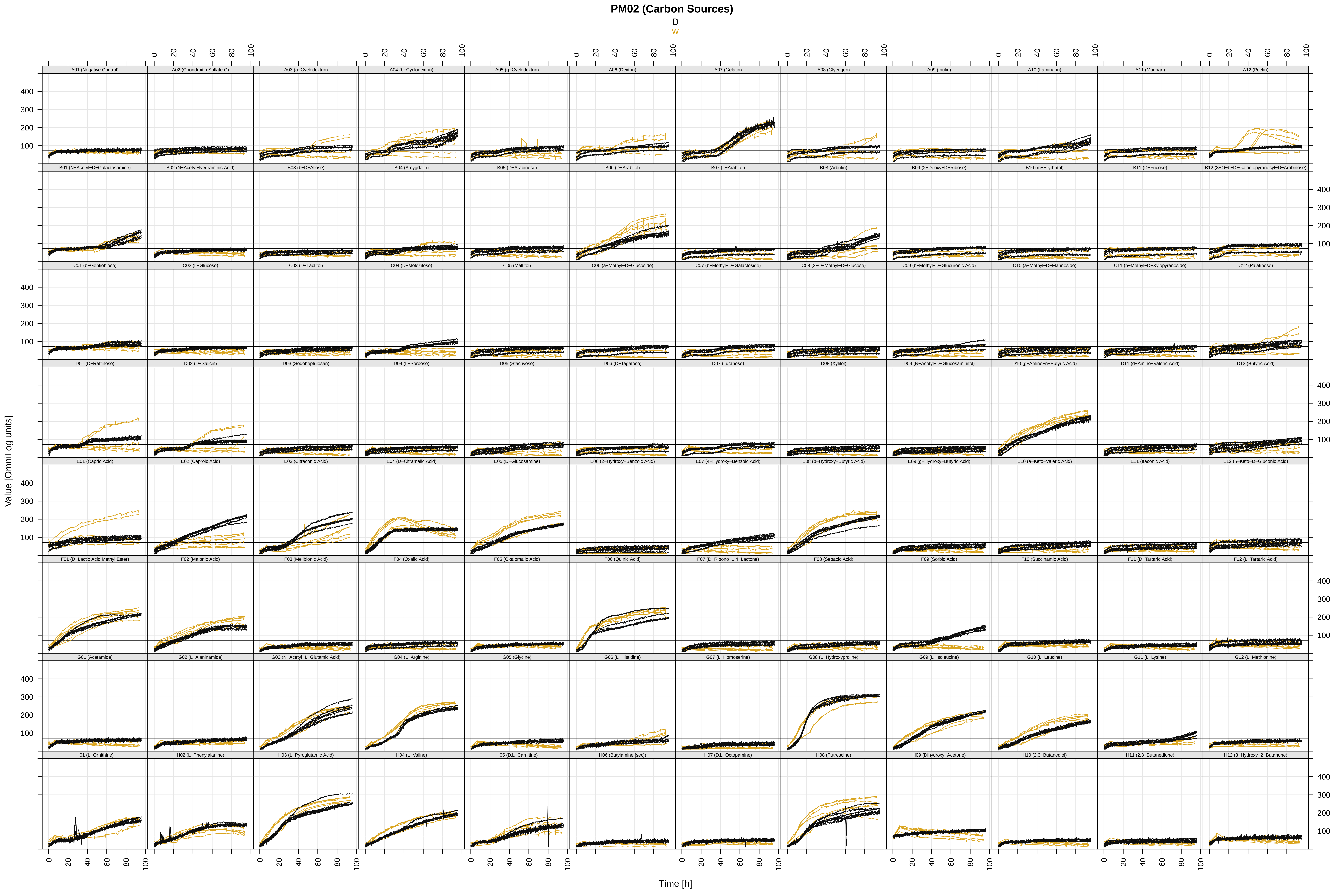
PM02 (Carbon Sources)
D
W
Value [OmniLog units]
Time [h]
A01 (Negative Control)
A02 (Chondroitin Sulfate C)
A03 (a−Cyclodextrin)
A04 (b−Cyclodextrin)
A05 (g−Cyclodextrin)
A06 (Dextrin)
A07 (Gelatin)
A08 (Glycogen)
A09 (Inulin)
A10 (Laminarin)
A11 (Mannan)
A12 (Pectin)
B01 (N−Acetyl−D−Galactosamine)
B02 (N−Acetyl−Neuraminic Acid)
B03 (b−D−Allose)
B04 (Amygdalin)
B05 (D−Arabinose)
B06 (D−Arabitol)
B07 (L−Arabitol)
B08 (Arbutin)
B09 (2−Deoxy−D−Ribose)
B10 (m−Erythritol)
B11 (D−Fucose)
B12 (3−O−b−D−Galactopyranosyl−D−Arabinose)
C01 (b−Gentiobiose)
C02 (L−Glucose)
C03 (D−Lactitol)
C04 (D−Melezitose)
C05 (Maltitol)
C06 (a−Methyl−D−Glucoside)
C07 (b−Methyl−D−Galactoside)
C08 (3−O−Methyl−D−Glucose)
C09 (b−Methyl−D−Glucuronic Acid)
C10 (a−Methyl−D−Mannoside)
C11 (b−Methyl−D−Xylopyranoside)
C12 (Palatinose)
D01 (D−Raffinose)
D02 (D−Salicin)
D03 (Sedoheptulosan)
D04 (L−Sorbose)
D05 (Stachyose)
D06 (D−Tagatose)
D07 (Turanose)
D08 (Xylitol)
D09 (N−Acetyl−D−Glucosaminitol)
D10 (g−Amino−n−Butyric Acid)
D11 (d−Amino−Valeric Acid)
D12 (Butyric Acid)
E01 (Capric Acid)
E02 (Caproic Acid)
E03 (Citraconic Acid)
E04 (D−Citramalic Acid)
E05 (D−Glucosamine)
E06 (2−Hydroxy−Benzoic Acid)
E07 (4−Hydroxy−Benzoic Acid)
E08 (b−Hydroxy−Butyric Acid)
E09 (g−Hydroxy−Butyric Acid)
E10 (a−Keto−Valeric Acid)
E11 (Itaconic Acid)
E12 (5−Keto−D−Gluconic Acid)
F01 (D−Lactic Acid Methyl Ester)
F02 (Malonic Acid)
F03 (Melibionic Acid)
F04 (Oxalic Acid)
F05 (Oxalomalic Acid)
F06 (Quinic Acid)
F07 (D−Ribono−1,4−Lactone)
F08 (Sebacic Acid)
F09 (Sorbic Acid)
F10 (Succinamic Acid)
F11 (D−Tartaric Acid)
F12 (L−Tartaric Acid)
G01 (Acetamide)
G02 (L−Alaninamide)
G03 (N−Acetyl−L−Glutamic Acid)
G04 (L−Arginine)
G05 (Glycine)
G06 (L−Histidine)
G07 (L−Homoserine)
G08 (L−Hydroxyproline)
G09 (L−Isoleucine)
G10 (L−Leucine)
G11 (L−Lysine)
G12 (L−Methionine)
H01 (L−Ornithine)
H02 (L−Phenylalanine)
H03 (L−Pyroglutamic Acid)
H04 (L−Valine)
H05 (D,L−Carnitine)
H06 (Butylamine [sec])
H07 (D,L−Octopamine)
H08 (Putrescine)
H09 (Dihydroxy−Acetone)
H10 (2,3−Butanediol)
H11 (2,3−Butanedione)
H12 (3−Hydroxy−2−Butanone)
0 20 40 60 80 100
100 200 300 400

PM03 (Nitrogen Sources)

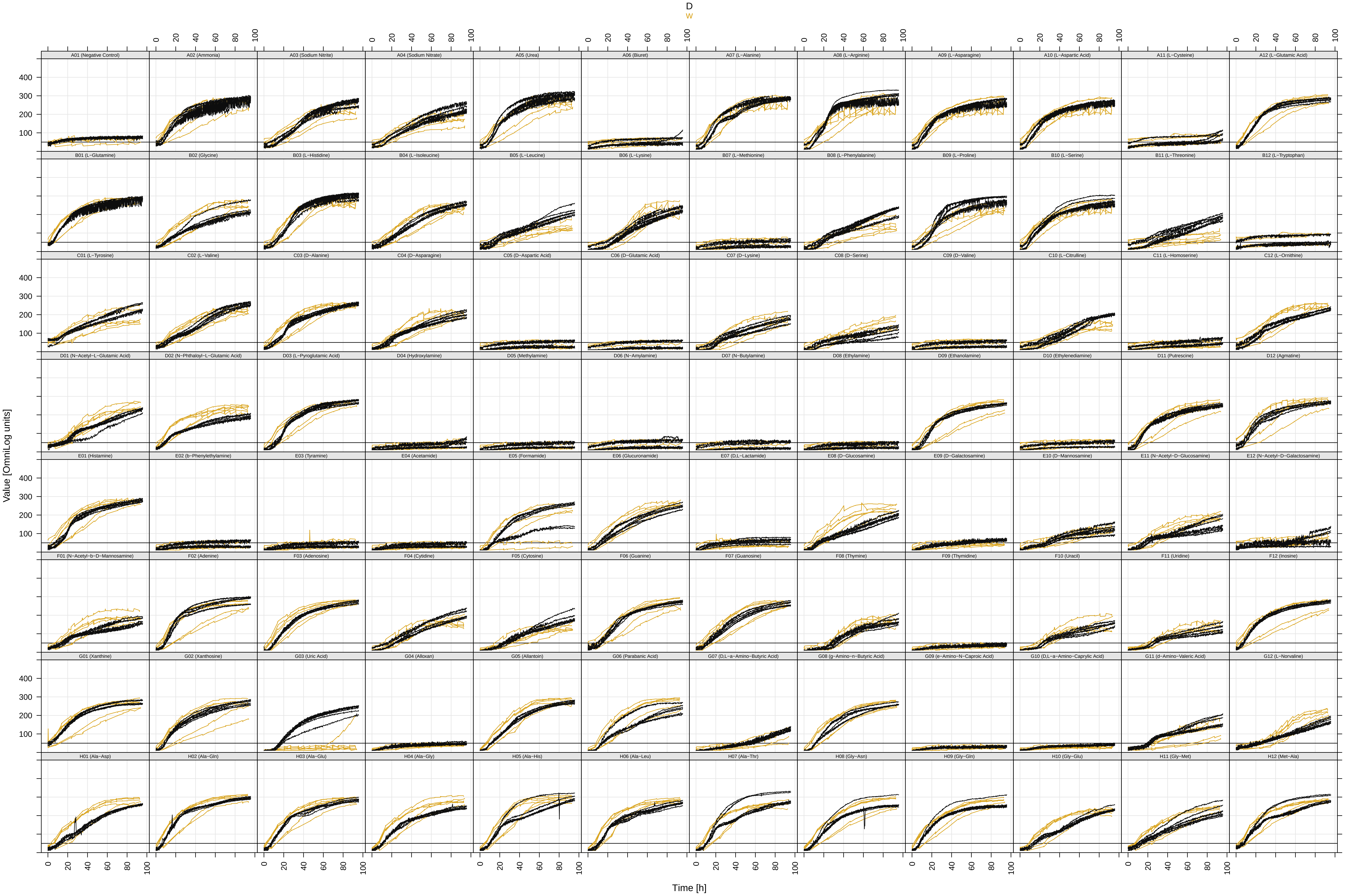

PM04 (Phosphorus and Sulfur Sources)

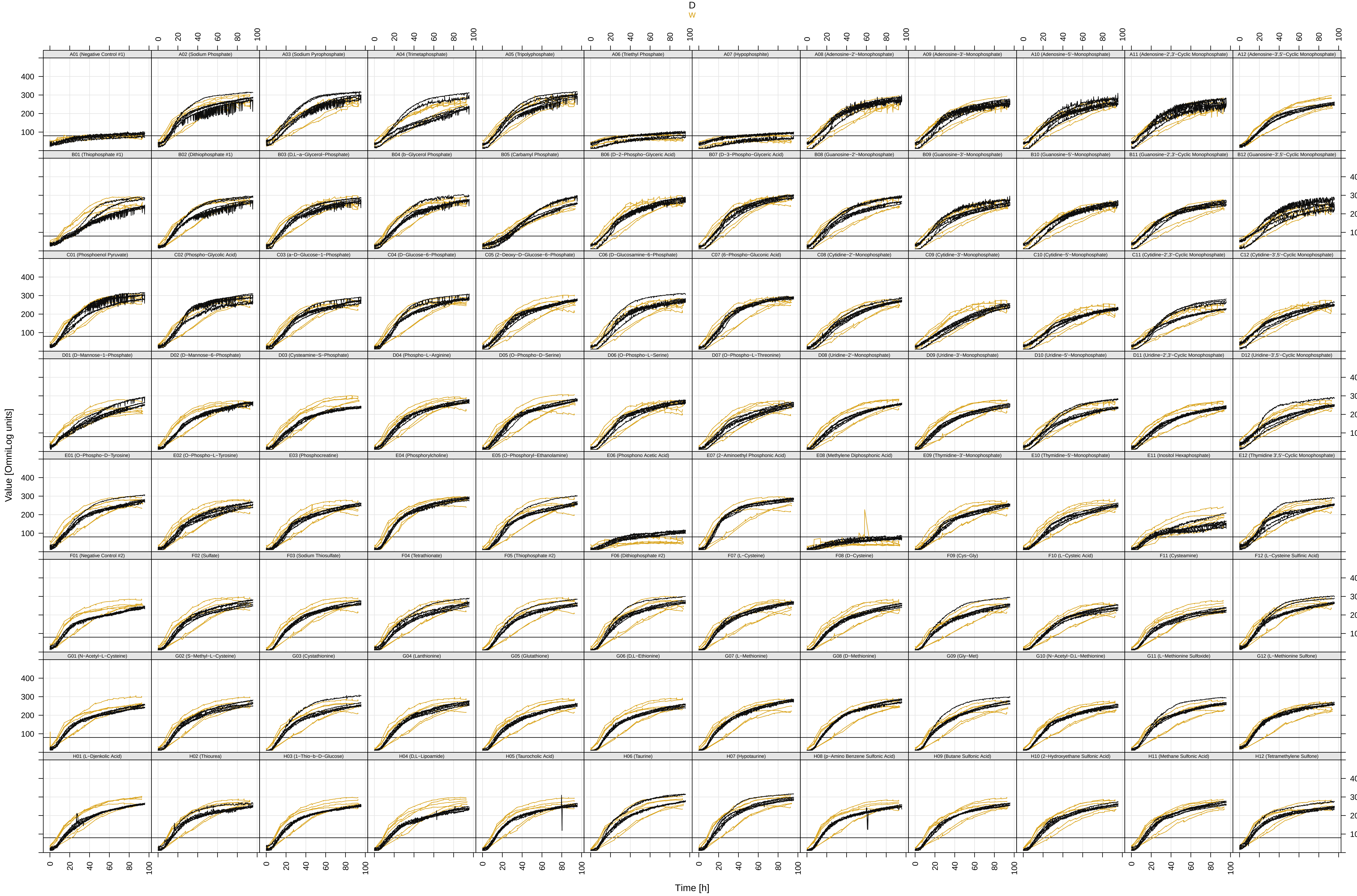

Supplement: S2 File — The x-axis displays incubation time (h) whereas the y-axis shows the substrates’ utilization in omnilog units. xy-plots depicting the raw kinetics of all replicates comparing each light condition to dark treatment displayed high reproducibility. Results on utilization of C sources are shown on page 1, 5, 9 (PM 1) and page 2, 6, 10 (PM 2), and of N as well as P and S sources on page 3, 7, 11 (PM 3) and page 4, 8, 12 (PM 4). (black line: dark incubation, blue line: blue LED, red line: red LED, yellow line: white LED). Substrate names are above each individual plot (see also S2 Table). (PDF) [file pone.0189862.s010.pdf]
