# Supplementary material for: Light spectrum modifies the utilization pattern of energy sources in Pseudomonas sp. DR 5-09
Source: PLoS One. 2017 Dec 21;12(12):e0189862. doi: 10.1371/journal.pone.0189862 (PMC5739431; doi:10.1371/journal.pone.0189862)
Supplement: S4 Fig — (PDF) [file pone.0189862.s006.pdf]

```
                            10        20        30        40        50        60        70        80        90       100
                   ....|....|....|....|....|....|....|....|....|....|....|....|....|....|....|....|....|....|....|....|
Pseudomonas sp.    MINAQLLQMVINASNDGIVIAEKEGEQDKILIYVNPAFERLTGYTSEEILYQDCRFLQSGDRDQEALPTIRDALNKEGSCREILRNYRKDGTPFWNELSL
P. fluorescens     ......M.L...................N...............HRD.................AS.EL..Q..KEG.A...V.................
P. moraviensis     ....S...L......E...V.....D..N...........M...S.............A.....PS.QL..ET.REG.......................
P. syringae        ....S......D................N.............................A.....A..AL..Q..DSG.......................
P. sp. DR 5-09     ....S..............V.....N..N..........K..................A.....AG.GV..E..KNG.......................
P. sp. DR 5-09     ....S..KL......E...V........N......S...M...S....................PG.AS..E..RNG.....V................V
P. Sp. DR 5-09     ....S..KL......E...V........N......S...M...S....................PG.AS..E..RNG.....V................V

                           110       120       130       140       150
                   ....|....|....|....|....|....|....|....|....|....|....|....
Pseudomonas sp.    STVKDPNDGQTYFVGVQKDVTTQVKAQQRVAQLESQLAEALAELSALKATNGANKTAN
P. fluorescens     ....NQA..H...........A........T...KE....RETIAR.E....S...T.
P. moraviensis     ....NAD......I.......VH...L.......A.V.ALQS..ATV...........
P. syringae        ....N.H..............V............A.....Q..IA...T...S.QS..
P. sp. DR 05-9     ....NAD..............V............A.V.ALE..IT.......E...T.
P. sp. DR 05-9     ....NAE..............V............A.VIALQ...AL.........S..*
P. sp. DR 05-9     ....NAE..............V............A.VIALQ...AL.........S..*
```
